# FDI-6 inhibits the expression and function of FOXM1 to sensitize BRCA-proficient triple-negative breast cancer cells to Olaparib by regulating cell cycle progression and DNA damage repair

**DOI:** 10.1038/s41419-021-04434-9

**Published:** 2021-12-08

**Authors:** Shu-Ping Wang, Shi-Qi Wu, Shi-Hui Huang, Yi-Xuan Tang, Liu-Qiong Meng, Feng Liu, Qi-Hua Zhu, Yun-Gen Xu

**Affiliations:** 1 State Key Laboratory of Natural Medicines and Jiangsu Key Laboratory of Drug Design and Optimization, China Pharmaceutical University, 211198 Nanjing, China; 2 Jiangsu Key Laboratory of Drug Design and Optimization, Department of Medicinal Chemistry, China Pharmaceutical University, Nanjing, 210009 China

**Keywords:** Targeted therapies, Mechanisms of disease, Mechanism of action, Pharmacodynamics, Oncogenes

## Abstract

Inducing homologous-recombination (HR) deficiency is an effective strategy to broaden the indications of PARP inhibitors in the treatment of triple-negative breast cancer (TNBC). Herein, we find that repression of the oncogenic transcription factor FOXM1 using FOXM1 shRNA or FOXM1 inhibitor FDI-6 can sensitize BRCA-proficient TNBC to PARP inhibitor Olaparib in vitro and in vivo. Mechanistic studies show that Olaparib causes adaptive resistance by arresting the cell cycle at S and G_2_/M phases for HR repair, increasing the expression of CDK6, CCND1, CDK1, CCNA1, CCNB1, and CDC25B to promote cell cycle progression, and inducing the overexpression of FOXM1, PARP1/2, BRCA1/2, and Rad51 to activate precise repair of damaged DNA. FDI-6 inhibits the expression of FOXM1, PARP1/2, and genes involved in cell cycle control and DNA damage repair to sensitize TNBC cells to Olaparib by blocking cell cycle progression and DNA damage repair. Simultaneously targeting FOXM1 and PARP1/2 is an innovative therapy for more patients with TNBC.

## Introduction

Poly(ADP-ribose) polymerases (PARPs) regulate various kinds of cell responses by transferring chains of ADP-ribose subunits to themselves or other target proteins [1]. Among the PARPs, PARP1/2 are important signaling molecules that involve in the repair of DNA single-strand break (SSB) through base excision repair (BER) pathway [2–5]. PARP1 is overexpressed in many cancers and correlated with poor prognosis [4–7]. DNA damage occurs under various conditions and can be divided into SSBs and double-strand breaks (DSBs) [8]. Numerous DNA damage repair pathways are triggered by different conditions [8]. However, only a few pathways could result in the precise repair of damaged DNA and maintain the stability of chromosomes [8, 9]. Homologous-recombination (HR) repair pathway is the most effective repair pathway that triggers precise repair of DSBs using a sister chromatid template for recombination [9, 10]. Multiple essential HR genes, including breast cancer susceptibility gene 1/2 (BRCA1/2) and recombinase Rad51, are recruited to damaged DNA to facilitate repair [9–12]. When the enzymatic activities of PARP1/2 are inhibited, DNA SSBs translate to DNA DSBs [13]. When HR repair pathway is deficient, DNA DSBs are unrepaired or misrepaired [13, 14]. Unrepaired DSBs induce cell death, and misrepaired DSBs promote unwanted chromosome rearrangements and genome instability [13]. Therefore, inhibition of PARP1 can effectively inhibit the growth of cancer cells with HR deficiency [14]. Several PARP inhibitors have been approved for the treatment cancers with HR deficiency [15]. Inhibiting PARP1 is a promising strategy for cancers with HR deficiency [15, 16].

Triple-negative breast cancer (TNBC), which accounted for 10–15% of breast cancers, is the most refractory subtype of breast cancer with a 5-year survival 8–16% [17]. Because lacking estrogen, progesterone receptor and human epidermal growth-factor receptor-2 (HER2) expression, estrogen therapy and HER2-targeted treatment do not work for TNBC [17, 18]. Chemotherapy remains the standard therapy for TNBC, despite its limited benefit [17–19]. Recently, advances with novel agents have been made for a specific subgroup with germline BRCA-mutated tumors [17]. In particular, the success of Olaparib and other PARP inhibitors in the treatment of TNBC with BRCA1/2 mutation brings hope to patients suffering from this refractory cancer [20]. However, the majority of TNBC still does not benefit from PARP1 inhibitors [20]. Meanwhile, PARP inhibitor-induced increasing of HR repair capacity, decreasing of replication stress, and diminishing of PARP1 trapping and other changes could induce the generation of drug resistance [21, 22]. Therefore, regulating HR capacity and/or cell cycle progression to sensitize more TNBC to PARP inhibitors is meaningful.

Recently, various new targets, reagents, and combination strategies have been explored to solve the limitation of PARP inhibitors, including the narrow clinical indications and adaptive resistance [20–22]. Inhibiting the enzymatic activity of kinases to induce HR deficiency is the mainly strategy to expand the indication of PARP inhibitors [20–22]. In addition to some kinases, transcription-factor forkhead box protein M1 (FOXM1) has been reported to involve in cell proliferation, cell cycle progression, DNA damage repair, apoptosis, and other biological processes [23, 24]. FOXM1 is characterized by an evolutionarily conserved “forkhead” DNA-binding domain and overexpressed in many tumors, including TNBC [23, 24]. Repression of FOXM1 has been reported to induce the death of cancer cells by inhibiting DNA damage repair and cell cycle progression [25, 26]. FOXM1 transcriptionally activates cyclin B1 (CCNB1), cell-division cycle 25 A (CDC25A), and cell-division cycle 25B (CDC25B) to regulate cell cycle [27]. BRCA2 and X-ray cross-completing group (XRCC1), the critical genes involved in HR repair, are essential downstream targets of FOXM1 [28]. FDI-6 has been reported to block FOXM1 binding to DNA and suppress the transcription of genes under FOXM1 control [29, 30]. Therefore, repressing FOXM1 to sensitize HR-proficient TNBC cells to Olaparib and other PARP inhibitors is a promising strategy. Herein, we selected the PARP inhibitor Olaparib and FOXM1 inhibitor FDI-6 to investigate their synergistic effect on the proliferation of BRCA-proficient TNBC cells in vitro and in vivo. Data in the Cancer Genome Atlas (TCGA) project showed that FOXM1 is generally overexpressed in tumor tissues but not in normal tissues. FDI-6 and Olaparib, at the ratio of 1:1 (FDI-6/Olaparib), synergistically inhibited the proliferation of BRCA-proficient TNBC cells in vitro and in vivo by blocking the progression of cell cycle and the precise repair of damaged DNA. The RNA sequencing results showed that Olaparib initiated adaptive resistance in MDA-MB-231 cells by inducing the recovery of HR repair capacity and acceleration of cell cycle progression. Unexpectedly, FDI-6 reversed Olaparib-induced adaptive resistance by inhibiting the expression of FOXM1 and PARP1/2 and regulating genes involved in cell cycle control and DNA damage repair. Simultaneously targeting PARP1/2 and FOXM1 is a promising strategy for more patients with TNBC.

## Materials and methods

### Reagents and antibodies

Anti-BRCA1 rabbit pAb (1:1000, No. 22362-1-AP) and anti-BRCA2 rabbit pAb (1:1000, No.19791-1-AP) were bought from Proteintech Group (Chicago, IL, USA). Anti-FOXM1 (D3F2B) rabbit mAb (1:1000, #20459), Anti-Rad51 (D4B10) rabbit mAb (1:1000, #8875), anti-β-Actin (8H10D10) mouse mAb (1:1000, #3700), anti-mouse igG-HRP-linked antibody (1:3000, #7076) and anti-rabbit igG-HRP-linked antibody (1:3000, #7074) were purchased from Cell Signaling Technology (Boston, MA, USA). Anti-CDC25A rabbit mAb (1:1000, ab156574), anti-CDC25B rabbit pAb (1:1000, ab156574), anti-CDK1 rabbit pAb (1:1000, ab201008), anti-CCNB1 rabbit pAb (1:1000, ab71977), anti-CCNA2 rabbit pAb (1:1000, ab181591), anti-CDK6 rabbit pAb (1:1000, ab124821), anti-CCND1 rabbit pAb (1:1000, ab16663), anti-CDK2 rabbit pAb (1:1000, ab32147), anti-CCNE2 rabbit pAb (1:1000, ab40890), anti-E2F2 rabbit pAb (1:1000, ab138515), anti-DCLK1 rabbit pAb (1:1000, ab109029), anti-MDC1 rabbit pAb (1:1000, ab241048), anti-XRCC1 rabbit pAb (1:1000, ab134056), anti-XRCC2 rabbit pAb (1:1000, ab124900), anti-PLK1 rabbit pAb (1:1000, ab189139), anti-PARP1 rabbit mAb (1:1000, ab191217), and anti-PARP2 rabbit mAb (1:1000, ab115620) were obtained from Abcam (Cambridge, England). Alexa Fluor 488 Anti-Human/mouse phospho-Histone H2AX (Ser139) mouse mAb (1:100, P16104) was bought from Affymetrix ebioscience (Santiago, CA, USA). FDI-6, Olaparib, bovine serum albumin (BSA), insulin, and glutathione were obtained from Sigma-Aldrich (St. Louis, MO, USA). Annexin V-FITC and PI apoptosis-detection kit, Annexin V-PE and 7-AAD apoptosis-detection kit, MTT cell proliferation assay kit, DAPI, comet assay kit, and crystal violet were bought from KeyGEN Biotech (Nanjing, Jiangsu, China). The stock solution of FDI-6 and Olaparib was prepared by transferring 10 mg to the DMSO at a concentration of 10 mM. Aliquots of the stock solutions were stored at −20 °C. All other chemicals used were analytical grade without purification.

### Cell culture

The BRCA-proficient TNBC cell lines MDA-MB-231 and MDA-MB-468 and the BRCA-deficient TNBC cell line HCC1937 were purchased from the Cell Resources Center of Shanghai Academy of Life Sciences (Shanghai, China). The high-mobility BRCA-proficient TNBC cell line MDA231-LM2 was obtained from the China Center for Type Culture Collection (Wuhan, Hubei, China). MDA-MB-231 cells, MDA-MB-468 cells, and MDA-231-LM2 cells were cultured in Leibovitz’s L-15 (KGM41300-500, KeyGEN Biotech, Nanjing, Jiangsu, China) with 10% fetal bovine serum (FBS). HCC1937 cells were cultured in RPMI-1640 (KGM31800N, KeyGEN Biotech, Nanjing, Jiangsu, China) with 10% FBS. All cells were incubated at 37 °C in a 5% CO_2_ atmosphere.

### shRNA lentivirus infection

Human recombinant FOXM1 shRNA lentivirus and the negative-control (NC) shRNA lentivirus were constructed by GeneChem Co., Ltd. (Shanghai, China). MDA-MB-231 cells were infected with FOXM1 shRNA, FOXM1 shRNA1, FOXM1 shRNA2, and NC shRNA lentiviruses using HitransG P promoting reagent according to the manufacturer’s instructions. Three days after MDA-MB-231 cell infection with shRNA lentiviruses, the expression of FOXM1 was measured by Q-PCR and Western blot. Puromycin was used to select for MDA-MB-231 cells stably expressing FOXM1 shRNA and NC shRNA. The sequences are listed in Supplementary Table 1.

### Drug-combination assay

The following combination ratios of FDI-6/Olaparib were selected: 1:0.25, 1:0.5, 1:1, 1:2, and 1:4. After TNBC cells were treated with FDI-6 and Olaparib at different combination ratios for 7 days, the viabilities of MDA-MB-231 cells, MDA231-LM2 cells, MDA-MB-468 cells and HCC1937 cells were measured by MTT assay. Untreated cells served as the control. The combination-index (CI) values were calculated by CompuSyn software using the equation CI = C_A,X_/IC_X,A_ + C_B,X_/IC_X,B_ [31]_._ Herein, C_A,X_ and C_B,X_ represent the concentrations of FDI-6 and Olaparib, respectively, that achieve an X% growth-inhibition ratio. IC_X, A_ and IC_X, B_ are the concentrations of single drugs (FDI-6 or Olaparib) that achieve the same growth-inhibition ratio. A CI value <1.0 represents synergy, CI = 1.0 represents additivity, and CI > 1.0 represents antagonism [31].

### Gene expression and correlation analysis

The differential expression of FOXM1, PARP1, and PARP2 between cancer tissues and adjacent normal tissues in TCGA project was analyzed by Gene Expression Profiling Interactive Analysis version 2 (GEPIA2). Box plots were performed to compare the differential expression of PARP1, PARP2, and FOXM1 between breast-invasive carcinoma tissues and adjacent normal tissues of the Genotype Tissue Expression (GTEx) database with the following settings: P-value cutoff = 0.01, log_2_FC cutoff = 1.0, and “match TCGA normal and GTEx data”. The correlation between PARP1 and FOXM1 in breast-invasive carcinoma from the TCGA project was also analyzed by GEPIA2.

### Colony-formation assay

All cells were seeded in 6-well plates at a concentration of 500 cells/well. For the silencing groups, MDA-MB-231 cells stably expressing FOXM1 shRNA and NC shRNA were cultured in medium containing 0.5 μM puromycin with 4.0 μM Olaparib for 14 days. For the drug-treated groups, TNBC cells were exposed to various concentrations of FDI-6 and/or Olaparib for 14 days. The medium was changed every 2–3 days to allow colony formation. After 14 days of treatment, the colonies were fixed with cold methanol for 10 min, stained with 0.1% crystal violet for 10 min, and imaged using an upright biological microscope (Olympus BX53, Tokyo, Japan). Experiments were repeated at least three times.

### Alkaline comet assay

An alkaline comet assay was performed using a comet assay kit (KeyGEN Biotech, Nanjing, Jiangsu, China) [31]. For the silencing groups, MDA-MB-231 cells stably expressing FOXM1 shRNA and NC shRNA were treated with 4.0 μM Olaparib for 7 days. For the drug-treated groups, MDA-MB-231 cells and MDA-MB-468 cells were treated with different concentrations of FDI-6, Olaparib, or their combination for 7 days. An alkaline comet assay was performed following the manufacturer’s protocol. In brief, cells (1 × 10^4^/ml) were mixed with low-melting-point agarose at a ratio of 1:10 (v/v), layered onto slides, lysed by lysis buffer at 4 °C for 2 h, and then unwound with alkaline-unwinding solution for another 30 min at room temperature. Following electrophoresis at 21 V for 30 min, the cells were stained with propidium iodide (PI) and observed with an inverted biological microscope (Olympus BX53, Tokyo, Japan). Five images were randomly captured per slide.

### Analysis of apoptosis and cell cycle

Cell apoptosis was analyzed by Annexin V-FITC and PI apoptosis-detection kits and Annexin V-PE and 7-amino-actinomycin D (7-AAD) apoptosis-detection kits (KeyGEN Biotech, Nanjing, Jiangsu, China). The cell cycle was analyzed by a PI cell cycle detection kit (KeyGEN Biotech, Nanjing, Jiangsu, China). For the silencing groups, MDA-MB-231 cells stably expressing FOXM1 shRNA and NC shRNA were treated with 4.0 μM Olaparib for 7 days. Cells were stained with Annexin V-PE and 7-AAD according to the manufacturer’s instructions to detect apoptosis. For the drug-treated groups, MDA-MB-231 cells and MDA-MB-468 cells were treated with different concentrations of FDI-6, Olaparib, or their combination for 7 days. After treatment, the cells were collected and stained with Annexin V-FITC and PI following the manufacturer’s protocol to analyze cell apoptosis. Cellular DNA was stained with PI following the manufacturer’s protocol. Cell apoptosis and the cell cycle were detected by BD FACSCelesta flow cytometry (New York, USA). Apoptosis data were analyzed by FlowJo 7.6, and the cell cycle data were analyzed by ModFit LT [31].

### Immunofluorescence assay

For the silencing groups, MDA-MB-231 cells stably expressing FOXM1 shRNA and NC shRNA were treated with 4.0 μM Olaparib for 7 days. For the drug-treated group, MDA-MB-231 cells and MDA-MB-468 cells were treated with different concentrations of FDI-6, Olaparib or their combination for 7 days. After treatment, cells were fixed with 4% formaldehyde, permeabilized with 0.2% (v/v) Triton X-100 in PBS, blocked with 1% (w/v) bovine serum albumin (BSA) in PBS for 1.0 h, and stained with anti-p-Histone 2AX (γH2AX) antibody labeled with Alexa Fluor 488. After staining, cellular DNA was counterstained with 4,6-diamidino-2-phenylindole (DAPI). Fluorescence signals were detected using a Carl Zeiss LSM700 laser confocal microscope (Jena, Germany). Five fields per sample were quantified.

### Animal care and treatment

All animal procedures were carried out in accordance with the institutional guidelines for the care and use of laboratory animals and approved by the committee on the ethics of Animal Experiments of China Pharmaceutic University. Every effort was made to ensure the comfort and safety of the animals. BALB/C nude mice (female, 5–6 weeks of age, weighting 18–20 g) and Swiss ICR mice (female/male, 5–6 weeks of age, weighting 18–20 g) were provided from the Animal House in Model Animal Institute of Nanjing University (Nanjing, Jiangsu, China). BALB/C nude mice were used to detect the in vivo antitumor activity of FDI-6, Olaparib, and their combination on the growth of MDA-MB-231 cells. Swiss ICR mice were used to detect the acute toxicity of FDI-6, Olaparib and their combination. For in vivo antitumor assay, MDA-MB-231 cells (5×10^6^) were injected into the right flank of each BALB/C nude mouse. After the average tumor volume (mm^3^) reached 100 mm^3^, the tumor-bearing mice were randomly divided into four groups (*n* = 5/group). FDI-6 (60 mg/kg), Olaparib (60 mg/kg), and the combination of FDI-6 (30 mg/kg) and Olaparib (30 mg/kg) were administered by intraperitoneal injection for 28 consecutive days. Tumor volume was recorded every two days. After 28 days of treatment, the mice were killed to detect the weight of tumors in each group. Tumor volume was measured with a Vernier caliper and calculated using the formula (ab^2^)/2, where a and b represent the length and width of the tumor, respectively. For acute-toxicity assay, Swiss ICR mice (female/male, 5–6 weeks of age, weighting 18–20 g) were divided into six groups (*n* = 10/group, female = 5, male = 5). Menstruum, FDI-6 (100 mg/kg, 200 mg/kg), Olaparib (200 mg/kg), and their combination (FDI-6 + Olaparib: 100 + 100 mg/kg, 200 + 200 mg/kg) were administered by intraperitoneal injection on the first day. After treatment, the weight and life status of ICR mice were recorded for seven consecutive days.

### RNA sequencing and data processing of DEGs

MDA-MB-231 cells were treated with Olaparib (8.0 μM), FDI-6 (8.0 μM), or the combination of FDI-6 (4.0 μM) and Olaparib (4.0 μM). After treatment for 7 days, cells were collected to obtain total RNA using TRIzol reagent (Vazyme, Nanjing, China) according to the manufacturer’s manual. A total of 500 ng of RNA was used to prepare libraries using the NEBNext Ultra RNA Library Perp Kit for Illumina. RNA quantity and quality were assessed on an Agilent 2100 Bioanalyzer. RNA library sequencing was performed on an Illumina HiSeqTM 2500/4000 by Gene Denovo Biotechnology Co., Ltd. (Guangzhou, Guangdong, China). Differentially expressed genes (DEGs) in the control group vs the Olaparib-treated group, the control group vs the FDI-6-treated group, and the control group *vs* the FDI-6/Olaparib cotreated group were identified based on a | log_2_FC | >1.0 and an adjusted P < 0.05. DEGs with a log_2_FC < 1.0 were considered downregulated genes, while DEGs with a log_2_FC > 1.0 were considered upregulated genes [32].

### Gene ontology and KEGG pathway enrichment analysis

The characteristic biological attributes of the DEGs were identified by gene ontology (GO) enrichment analysis. The functional attributes of the DEGs were identified by KEGG pathway enrichment analysis. The GO enrichment and KEGG pathway enrichment analyses were performed using Omicsmart, a real-time interactive online platform for data analysis (http://www.omicsmart.com) [32].

### PPI network construction and module analysis

Four DEGs were selected based on the GO and KEGG enrichment analyses. The Search Tool for the Retrieval of Interacting Genes (STRING) was used to evaluate and establish the protein–protein interaction (PPI) networks of PARP1, PARP2, FOXM1 and the four DEGs. The STRING application in Cytoscape was used to detect the potential correlations between these DEGs. The MCODE application in Cytoscape was utilized to examine the modules of the PPI network and identify the central genes among the DEGs (degree cutoff = 2, max. depth = 100, k-core = 2, and node-score cutoff = 0.2) [32].

### Q-PCR analysis

Total RNA in MDA-MB-231 cells stably expressing FOXM1 shRNA and NC shRNA, MDA-MB-231 cells, MDA-MB-468, cells and MDA-MB-231 xenografts was extracted with TRIzol reagent (Vazyme, Nanjing, Jiangsu, China) according to the manufacturer’s instructions [31]. cDNA was synthesized using a HiScript II one-step RT-PCR kit (P612-01, Vazyme, Nanjing, China) with 1.0 μg of total RNA in a 20 μl reaction system. The resulting cDNA was diluted 1:2 in nuclease-free water, and 1.0 μl was used per Q-PCR in triplicate. Q-PCR was carried out using ChamQ SYBR Q-PCR master mix (Q311-02, Vazyme, Nanjing, China) on a QuantStudio 3 real-time PCR-detection system (Life Tech, New York, USA) including a nontemplate negative control. GAPDH was used to normalize the level of mRNA expression. The sequences of the primers are listed in Supplementary Table 2.

### Western blot analysis

Total proteins in MDA-MB-231 cells stably expressing FOXM1 shRNA and NC shRNA, MDA-MB-231 cells, and MDA-MB-231 xenografts were extracted with RIPA cell-lysis buffer (Beyotime, Shanghai, China) with added protease/phosphatase-inhibitor cocktail. For nuclear-fraction assays, MDA-MB-231 cells were collected and lysed with cytoplasmic extraction buffer from the Nuclear/Cytoplasmic Protein Extraction Kit (KGP1100, KeyGEN Biotech, Nanjing, Jiangsu, China) and then centrifuged at 3000 rpm for 10 minutes. The supernatant was labeled as the cytoplasmic extract. The pellets were further lysed with nuclear-extraction buffer to obtain nuclear extracts. Protein concentration was determined by the BCA assay, and equal amounts of proteins were loaded for Western blot analysis. In brief, equal amounts of total proteins were loaded for SDS-PAGE and transferred onto a PVDF membrane (Millipore, Billerica, MA, USA). Membranes with protein were blocked with 5% (w/v) skim milk, incubated with primary antibody in Supplementary Materials, and then incubated with secondary antibodies (1:2000) for detection. β-Actin and Histone H3 were used to normalize the level of protein expression. Densitometric analysis was performed with ImageJ software.

### H&E staining

The lung, liver, heart, kidney, and spleen-tissue samples of xenograft model mice were fixed with 4% paraformaldehyde, dehydrated with ethanol, immersed in xylene, embedded in paraffin, and cut into 4.0-μm longitudinal sections. The paraffin-embedded sections were stained with hematoxylin and eosin (H&E) according to the manufacturer’s instructions (Beyotime, Shanghai, China). Each group of samples was observed with a DM6B-positive fluorescence microscope (Leica, Frankfurt, Germany). Five images were randomly captured per slide.

### Immunohistochemical staining

The tumors from xenograft-model mice were embedded in paraffin and cut into longitudinal sections. The paraffin-embedded sections were incubated with 0.3% hydrogen peroxide for 30 min to block endogenous peroxidase and then incubated with 1.0% bovine serum albumin (BSA) for blocking. After blocking, the paraffin-embedded sections were incubated with the primary antibody overnight at 4 °C, incubated with secondary antibody for another 1 h at room temperature and then counterstained for 1 min with hematoxylin. Each group was examined using a DM6B-positive fluorescence microscope (Leica, Frankfurt, Germany). Five images were randomly captured per slide. The percentage of stained dots was analyzed by ImageJ software.

### Statistical analysis

The data were analyzed using SPSS 19.0. The results are expressed as means ± SD. Differences between treatment regimens were analyzed by two-tailed Student’s *t*-test or one-way ANOVA. P < 0.05 was considered statistical significance.

## Results

### FOXM1 repression inhibits PARP1 expression and sensitizes TNBC cells to Olaparib

**Fig. 1:**
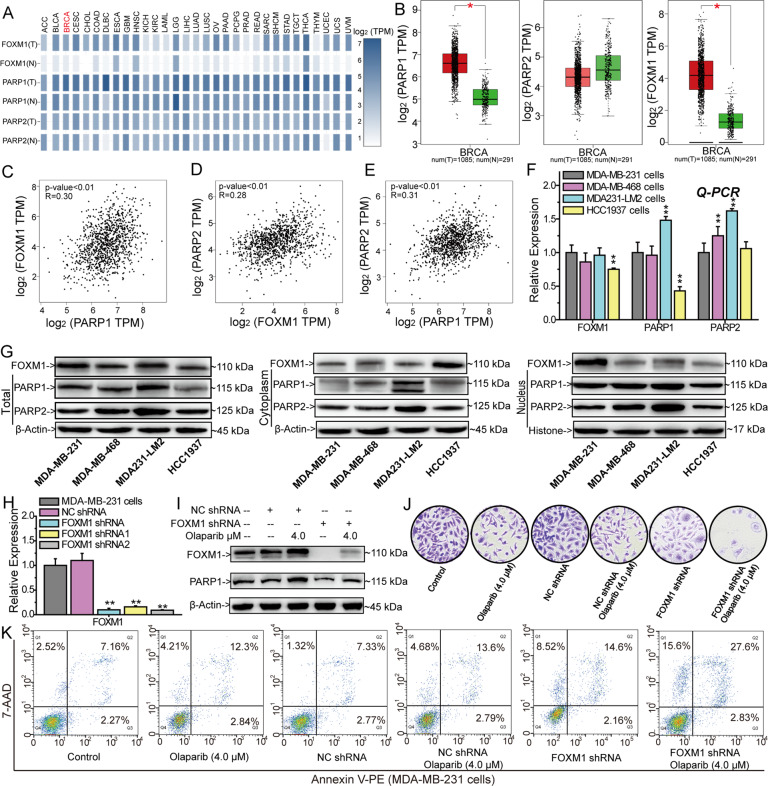
FOXM1 knockdown inhibits PARP1 expression and sensitizes TNBC cells to Olaparib. **A** Differential expression of FOXM1, PARP1, and PARP2 between cancer tissues and adjacent tissues in TCGA project. **B** Box plots for the expression of PARP1, PAPR2, and FOXM1 between breast-invasive carcinoma tissues and adjacent tissues in TCGA project. Expression correlation analysis of PARP1 *vs* FOXM1 (**C**), PARP2 *vs* FOXM1 (**D**) and PARP1 *vs* PARP2 (**E**) based on the TCGA project samples. Relative expression of FOXM1, PARP1, and PARP2 in TNBC cell lines analyzed by Q-PCR (**F**) and Western blots (**G**). **H** Silencing FOXM1 expression by FOXM1 shRNA. **I** FOXM1 shRNA inhibits PARP1 and FOXM1 expression. FOXM1 shRNA and Olaparib synergistically inhibited the formation of MDA-MB-231 cell colonies (**J**) and promoted the apoptosis of MDA-MB-231 cells (**K**). The results from three independent experiments were statistically analyzed using a *t-test*: ^*^P < 0.05, ^**^P < 0.01 compared with MDA-MB-231 cells.

Transcription factor FOXM1, involving in HR-mediated repair, is a promising target for solving the limitation of Olaparib and other PARP inhibitors [23, 24]. To evaluate whether FOXM1 is a valuable target, we analyzed the differential expression of FOXM1, PARP1, and PARP2 between cancer tissues and adjacent normal tissues in 31 cancers from the TCGA projects (Fig. 1A, B and Supplementary Fig. 1A, B). FOXM1 and PARP1 were generally overexpressed in various cancer tissues (Fig. 1A, B and Supplementary Fig. 1A, B). Compared with PARP1/2, the expression levels of FOXM1 in normal tissues were very low (Fig. 1A, B). Targeting FOXM1 would kill tumor cells in a selective way, which could avoid the toxicity to normal cells. Subsequently, we analyzed the correlation of FOXM1 *vs* PARP1, FOXM1 *vs* PARP2 and PARP1 *vs* PARP2 using the data from TCGA project (Fig. 1C–E). The results showed that FOXM1 expression was positively correlated with PARP1 (R = 0.3, P < 0.01) and PARP2 (R = 0.28, P < 0.01) expression, which suggested that FOXM1 and PARP1/2 could promote the expression of each other (Fig. 1C, D). The results of Q-PCR and Western blots showed that the expressions of FOXM1 and PARP1/2 were high in the four TNBC cells, and the nuclear accumulation of FOXM1 was the highest in MDA-MB-231 cells (Fig. 1F, G and Supplementary Fig. 2). Silencing FOXM1 repressed Olaparib-induced PARP1 expression, inhibited colony formation and promoted cell apoptosis (Fig. 1H–K and Supplementary Fig. 2). Moreover, silencing FOXM1 enhanced the effects of Olaparib on colony formation and cell apoptosis (Fig. 1J, K). Thus, FOXM1 knockdown increased the sensitivity of BRCA-proficient TNBC cells to Olaparib.

### FDI-6 and Olaparib synergistically inhibit the growth of TNBC cells in vitro and in vivo

**Fig. 2:**
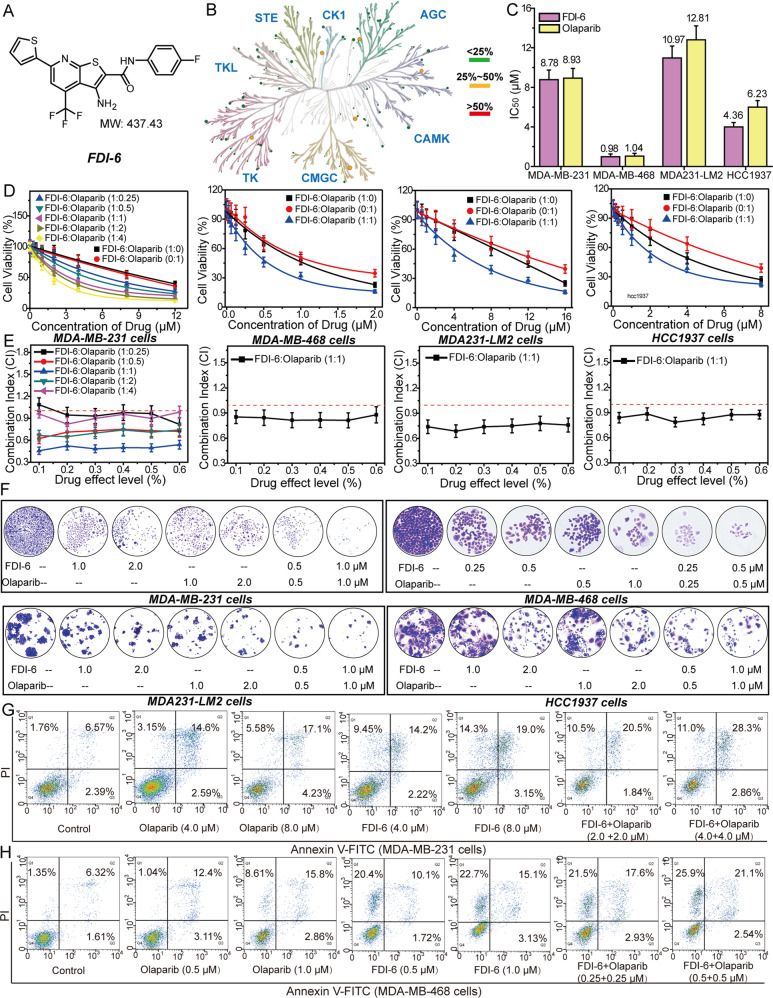
FDI-6 and Olaparib synergistically inhibit the growth of TNBC cells in vitro. **A** The structure of FDI-6. **B** The kinase-selectivity profile of FDI-6 using an RBC kinase panel screen against 361 kinases assayed at 1.0 μM in duplicate. **C** IC_50_ values for the effects of FDI-6 and Olaparib on the viability of TNBC cells. **D** The effects of combining FDI-6 and Olaparib on the proliferation of TNBC cells. **E** CI values for concurrent treatment with FDI-6 and Olaparib in TNBC cells. The combination ratios of FDI-6/Olaparib are 1:0.25, 1:0.5, 1:1, 1:2, and 1:4. **F** The effects of FDI-6 and/or Olaparib on the proliferation of TNBC cells measured by colony-forming assay. The effects of FDI-6 and/or Olaparib on the apoptosis of MDA-MB-231 cells (**G**) and MDA-MB-468 cells (**H**).

FDI-6 is a small-molecular inhibitor of FOXM1 selected by high-throughput screening [30]. To confirm whether it is suitable to be a tool molecular, we tested the selectivity of FDI-6 against 361 kinases at the concentration of 1.0 μM in the Reaction Biology Corporation kinase panel (Fig. 2A, B and Supplementary Table 3). The inhibition ratios of FDI-6 against most kinases were under 20%, which proved that FDI-6 is a selective inhibitor of FOXM1. After 7 days of treatment, the proliferation of the four TNBC cell lines was significantly inhibited by FDI-6 and Olaparib (Fig. 2C). The synergistic effects of FDI-6, Olaparib, and their combination at fixed dose ratios on the proliferation of the four TNBC cells were investigated (Fig. 2D, E). Combining FDI-6 with Olaparib not only inhibited the growth of BRCA-deficient cells but also significantly inhibited the growth of BRCA-proficient MDA-MB-231, MDA-MB-468 and MDA 231-LM2 cells. FDI-6 and Olaparib synergistically inhibited the growth of the four TNBC cell lines at the dose ratio of 1:1 (CI < 1.0). Therefore, we selected the dose ratio of 1:1 for further study. FDI-6 and Olaparib synergistically inhibited the formation of colonies and promoted the apoptosis of MDA-MB-231 cells and MDA-MB-468 cells (Fig. 2F–H and Supplementary Fig. 3A).

### FDI-6 inhibits FOXM1 and PARP1/2 expression to sensitize MDA-MB-231 xenografts to Olaparib

**Fig. 3:**
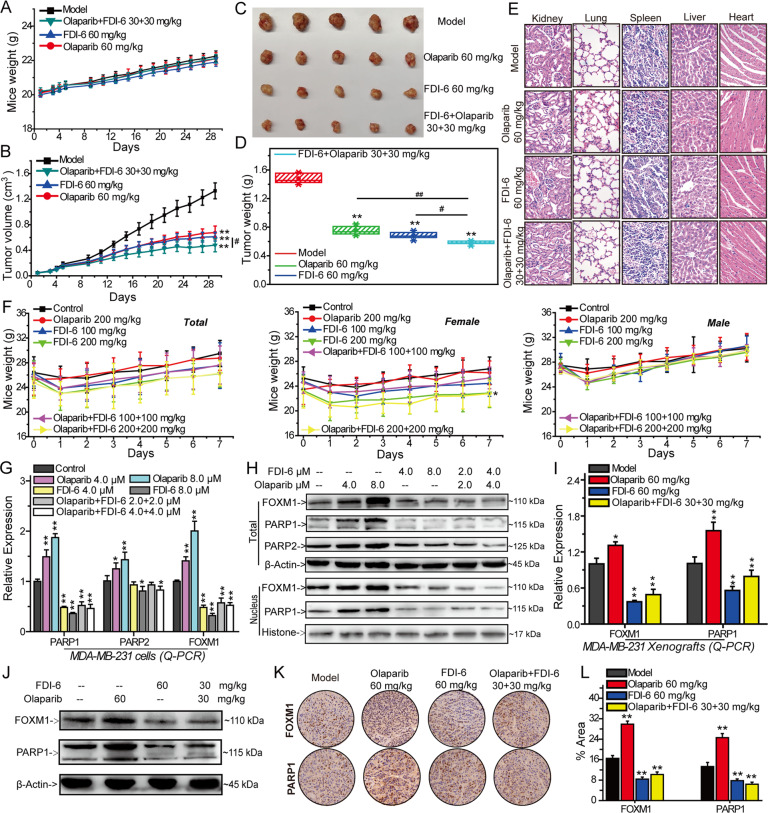
FDI-6 sensitizes MDA-MB-231 xenografts to Olaparib and reverses Olaparib-induced FOXM1 and PARP1/2 expression in vitro and in vivo. MDA-MB-231 cells were injected into nude mice and the mice were subsequently treated with FDI-6 and/or Olaparib at the indicated times. The mice weight (**A**), tumor volume (**B**), tumor nodules (**C**), and tumor weight (**D**) in each group. **E** Tissue damage was determined by H&E staining. **F** Acute-toxicity study of FDI-6, Olaparib and their combination in Swiss ICR mice. **G** Relative expression of PARP1, PARP2 and FOXM1 in MDA-MB-231 cells analyzed by Q-PCR. **H** The distribution of PARP1 and FOXM1 in the nucleus analyzed by Western blots. Q-PCR (**I**), Western bolts (**J**), and immunohistochemical (**K**) analyses of PARP1 and FOXM1 expression in vivo. **L** Percentage of immunohistochemically stained dots. The results from three independent experiments were statistically analyzed using one-way ANOVA: ^*^P < 0.05, ^**^P < 0.01 compared with the control; ^#^*P* < 0.05, ^##^*P* < 0.01 compared with the FDI-6/Olaparib combined group (FDI-6/Olaparib: 30 + 30 mg/kg).

MDA-MB-231 xenograft tumor model of nude mice was established to investigate the synergistic effect of FDI-6 and Olaparib on the growth of MDA-MB-231 cells in vivo. After 28 days of treatment, FDI-6 and Olaparib synergistically inhibited tumor size and tumor weight, but did not affect the body weight of mice at our tested dosages (Fig. 3A–D and Supplementary Fig. 3B). After analysis of H&E-stained organs, we found that FDI-6, Olaparib and their combination did not cause significant damage to the kidney, lung, spleen, liver or heart (Fig. 3E). The results of acute-toxicity experiment showed that FDI-6, Olaparib and their combination did not induce the death of mice and significantly affect their life status. FDI-6 (200 mg/kg) and the combination of FDI-6 (200 mg/kg) and Olaparib (200 mg/kg) induced the decreasing of female-mice weight but did not affect the weight of male mice (Fig. 3F). The results of Q-PCR and Western blots showed that Olaparib increased the expression of FOXM1 and PARP1/2 in vitro and in vivo (Fig. 3G–J, Supplementary Fig. 4A–C, Supplementary Fig. 5 and Supplementary Fig. 6). FDI-6 inhibited the expression of FOXM1 and PARP1 and impaired Olaparib-induced overexpression of FOXM1 and PARP1 in vitro and in vivo (Fig. 3G–J and Supplementary Fig. 4A–C). Further investigation of immunohistochemistry confirmed that FDI-6 could reverse Olaparib-induced accumulation of FOXM1 and PARP1 in vivo (Fig. 3K, L).

### FDI-6 and Olaparib regulate genes involved in cell cycle progression and DNA damage repair

**Fig. 4:**
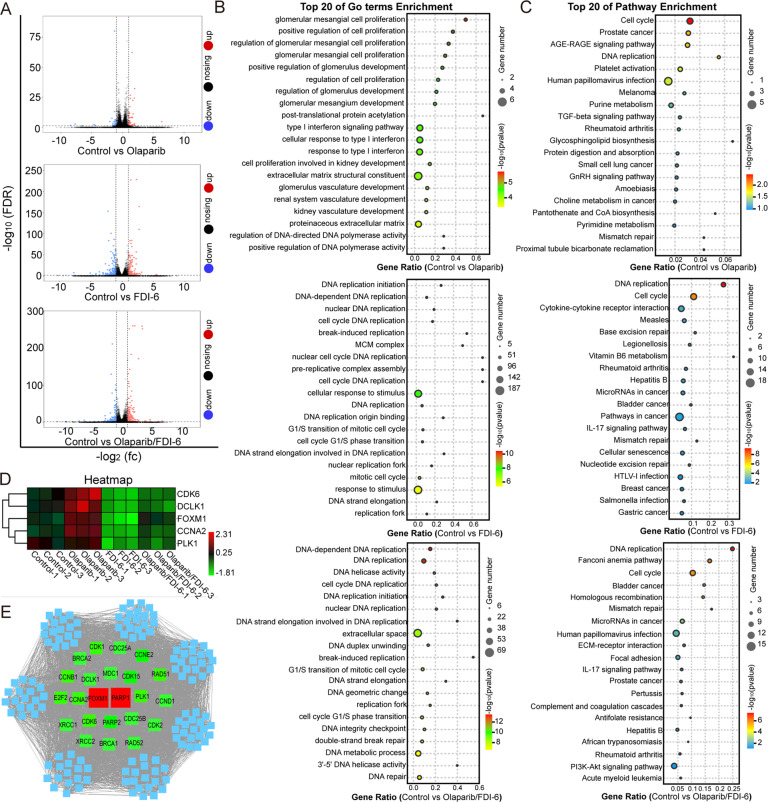
FDI-6 and Olaparib regulate cell cycle progression and DNA damage repair as indicated by the RNA sequencing analysis. **A** Volcano plot shows DEGs induced by FDI-6 and/or Olaparib. GO enrichment (**B**) and KEGG pathway enrichment (**C**) analysis of DEGs. **D** Heatmap plot of the five DEGs. **E** The PPI network was used to analyze the interactions among PARP1, PARP2, the five proteins, and other proteins expanded from the STRING database using Cytoscape 3.7.

RNA sequencing was used to analyze the effects of FDI-6, Olaparib and their combination on total gene expression in MDA-MB-231 cells (Fig. 4A–D). After 7 days of treatment, FDI-6 (8.0 μM) significantly upregulated 133 gene expressions and downregulated 181 gene expressions. Olaparib (8.0 μM) significantly promoted the expression of 51 genes, and inhibited the expression of 39 genes. Combining FDI-6 (4.0 μM) with Olaparib (4.0 μM) induced 202 upregulated DEGs and 200 downregulated DEGs (Fig. 4A, Supplementary Table 4, Supplementary Table 5 and Supplementary Table 6) After GO functional enrichment, we found that DEGs induced by Olaparib were mostly associated with cell proliferation, DEGs induced by FDI-6 were mostly associated with DNA replication, and DEGs induced by the combination of FDI-6 and Olaparib were mostly associated with DNA replication (Fig. 4B). The results of KEGG pathway enrichment showed that the main pathways associated with the DEGs were the cell cycle and DNA replication (Fig. 4C). By a pairwise comparison of control group vs Olaparib-treated group, control group *vs* FDI-6-treated group and control group *vs* the FDI-6/Olaparib cotreated group, we selected five DEGs to analyze their expression. Olaparib significantly promoted the expression of cyclin-dependent kinase 6 (CDK6), FOXM1, double-cortin-like kinase 1 (DCLK1), and cyclin A1 (CCNA1). FDI-6 inhibited the expression of CDK6, FOXM1, DCLK1, CCNA1, and polo-like kinase 1 (PLK1) (Fig. 4D). Subsequently, we constructed a PPI network using PARP1, PARP2, and the five genes as the centers by STRING. As shown in Fig. 4E, 23 genes were selected for further mechanistic investigation.

### FDI-6 inhibits Olaparib-induced promotion of cell cycle progression

**Fig. 5:**
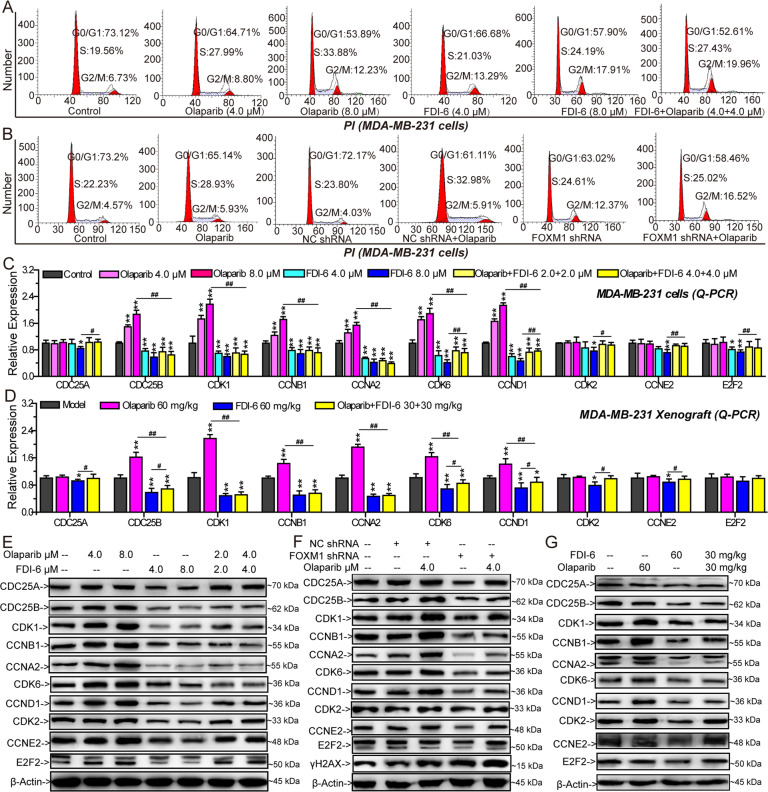
The effects of FDI-6 and/or Olaparib on cell cycle progression and genes involved in cell cycle control. **A** The effects of FDI-6 and/or Olaparib on cell cycle progression in MDA-MB-231 cells. **B** The effects of FOXM1 shRNA and/or Olaparib on cell cycle progression in MDA-MB-231 cells. Relative expression of genes involved in cell cycle control analyzed by Q-PCR in MDA-MB-231 cells (**C**) and MDA-MB-231 xenografts (**D**). **E** The effects of FDI-6 and/or Olaparib on the expression of proteins involved in cell cycle control analyzed by Western blots in MDA-MB-231 cells. **F** The effects of FOXM1 shRNA and/or Olaparib on the expression of proteins involved in cell cycle control analyzed by Western blots in MDA-MB-231 cells. **G** The effects of FDI-6 and/or Olaparib on the expression of proteins involved in cell cycle control analyzed by Western blots in vivo. The results from three independent experiments were statistically analyzed using one-way ANOVA: ^*^P < 0.05, ^**^P < 0.01 compared with the control; ^#^*P* < 0.05, ^##^*P* < 0.01 compared with the FDI-6/Olaparib-combined group (FDI-6/Olaparib: 4.0 + 4.0 μM, FDI-6/Olaparib: 30 + 30 mg/kg).

Olaparib promoted the G_1_/S transition and arrested the cell cycle at the S and G_2_/M phases in MDA-MB-231 cells and MDA-MB-468 cells. FDI-6 inhibited the G_2_/M transition and arrested the cell cycle at the G_2_/M phase. Combining FDI-6 with Olaparib inhibited the G_2_/M transition and arrested the cell cycle at the G_2_/M phase (Fig. 5A and Supplementary Fig. 7A). Similarly, silencing the expression of FOXM1 blocked the G_2_/M transition and arrested the cell cycle at the G_2_/M phase (Fig. 5B). Q-PCR and Western blots were used to analyze the expression of genes involved in cell cycle control based on the results of bioinformatic analysis in vitro and in vivo (Fig. 5C–G, Supplementary Fig. 7B, Supplementary Fig. 8A–C, Supplementary Fig. 9 and Supplementary Fig. 10). CDK6, cyclin D1 (CCND1), cyclin-dependent kinase 2 (CDK2), cyclin E2 (CCNE2), CDC25A and E2F transcription factor 2 (E2F2) regulate the G_1_/S transition, and cyclin-dependent kinase 1 (CDK1), CCNA1, CCNB1, and CDC25B regulate the G_2_/M transition [33]. Olaparib significantly promoted the expression of CDC25B, CDK1, CCNA1, CCNB1, CDK6 and CCND1 to accelerate mitosis. FDI-6 significantly inhibited the expression of the ten genes-involved in cell cycle control and impaired Olaparib-induced expression of the six genes in MDA-MB-468 cells, MDA-MB-231 cells, and MDA-MB-231 tumor xenografts (Fig. 5C–G, Supplementary Fig. 7B and Supplementary Fig. 8A–C).

### FDI-6 promotes Olaparib-induced DNA damage by inhibiting precise repair of damaged DNA in vitro and in vivo

**Fig. 6:**
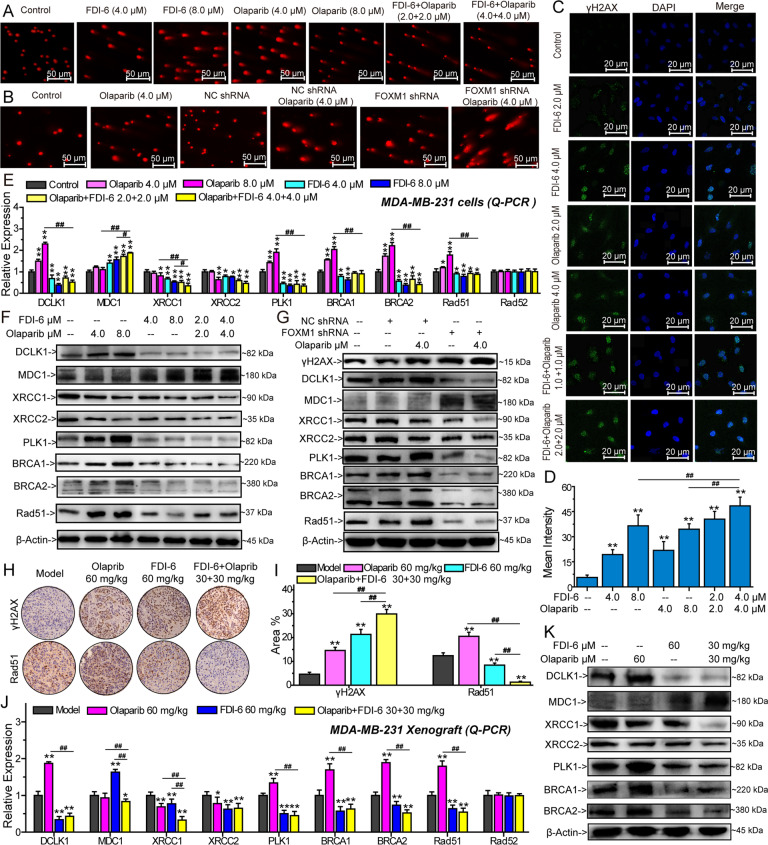
The effects of FDI-6 and/or Olaparib on DNA damage and genes involved in DNA repair. **A** The effects of FDI-6 and/or Olaparib on DNA damage detected by alkaline comet assay. **B** The effects of FOXM1 shRNA and/or Olaparib on DNA damage detected by alkaline comet assay. **C** The expression of γh2AX analyzed by immunofluorescence. Green represents the cells stained with anti-γh2AX antibody, blue represents the nuclei stained with DAPI. **D** Mean intensity of immunofluorescence. The effects of FDI-6 and/or Olaparib on the expression of DNA repair-related factors analyzed by Q-PCR (**E**) and (**F**) in MDA-MB-231 cells. **G** The effects of FOXM1 shRNA and/or Olaparib on the expression of DNA repair-related proteins analyzed by Western blots. **H** Effects of FDI-6 and/or Olaparib on the expression of γh2AX and Rad51 in vivo analyzed by immunohistochemistry. **I** Percentage of immunohistochemically stained dots. Effects of FDI-6 and/or Olaparib on the expression of factors involved in DNA repair analyzed by Q-PCR (**J**) and Western blots (**K**). The results from three independent experiments were statistically analyzed using one-way ANOVA: ^*^P < 0.05, ^**^P < 0.01 compared with the control; ^#^*P* < 0.05, ^##^*P* < 0.01 compared with the FDI-6/Olaparib-combined group (FDI-6/Olaparib: 4.0 + 4.0 μM, FDI-6/Olaparib: 30 + 30 mg/kg).

Damaged DNA has a tail in the comet assay that resembles a comet [34]. FDI-6, Olaparib, and their combination significantly increased the number and extent of tailed DNA. FOXM1 knockdown also promoted DNA damage, and the number and extent of tailed DNA were significantly increased (Fig. 6A, B and Supplementary Fig. 11A). The accumulation of γH2AX also reflects the extent of DNA damage [35]. The results of immunofluorescence showed that FDI-6, Olaparib, and their combination significantly increased nuclear γh2AX levels (Fig. 6C, D). Subsequently, we found that Olaparib significantly increased the expression of DCLK1, mediator of DNA damage checkpoint (MDC1), PLK1, BRCA1, BRCA2, and Rad51, and inhibited the expression of XRCC1 and X-ray cross-complementing group-2 protein (XRCC2) in vitro. FDI-6 and FOXM1 shRNA promoted MDC1 expression and inhibited the expression of DCLK1, PLK1, BRCA1, BRCA2, Rad51, XRCC1, and XRCC2 (Fig. 6E–G, Supplementary Fig. 11B, C, Supplementary Fig. 12A, Supplementary Fig. 13 and Supplementary Fig. 14). Further in vivo investigation showed that FDI-6, Olaparib, and their combination induced DNA damage in vivo, and the expression of γH2AX was significantly increased (Fig. 6H, I). The regulation of FDI-6, Olaparib and their combination on the expression of genes involved in DNA repair was similar to their efficacy in vitro (Fig. 6H–K, Supplementary Fig. 12B and Supplementary Fig. 14).

## Discussion

Currently, a few targeted therapies approved for the treatment of TNBC, the most challenging breast cancer subtype that has the poorest prognosis and median-survival rate after relapse [20]. Targeting PARP1/2 for synthetic lethality is an effective strategy for TNBCs with BRCA1/2 mutations [20]. However, the majority of TNBCs are BRCA1/2 wild type, and their prognosis remains poor, particularly after relapse [17–20]. Therefore, inducing HR deficiency is an effective strategy to broaden the indication of PARP inhibitors. Oncogenic transcription factor FOXM1 is overexpressed in breast cancer and plays important roles in DNA damage repair by regulating the expression of genes essential for DNA damage sensing, mediation, signaling, and repair, as well as for cell cycle and cell death control [23, 24]. Targeting FOXM1 in monotherapy or combination therapy may have promising therapeutic benefits [14, 28]. Herein, we inhibited the expression and function of FOXM1 to investigate its effects on the sensitivity of BRCA-proficient TNBC cells to Olaparib.

After bioinformatics analysis, we found that the expression levels of FOXM1 and PARP1 were higher in breast invasive carcinoma tissue than in adjacent tissue. FOXM1 expression was positively correlated with PARP1 expression in breast-invasive carcinoma. Olaparib induced the overexpression of FOXM1 and PARP1/2. This kind of change induces adaptive resistance of TNBC cells to Olaparib [21, 22]. Silencing FOXM1 inhibited the expression of PARP1/2 and impaired Olaparib-induced overexpression of PARP1/2 to sensitize BRCA-proficient TNBC cells to Olaparib. Meanwhile, FOXM1 inhibitor FDI-6 and Olaparib at the ratio of 1:1 synergistically inhibited the growth of BRCA-proficient TNBC cells in vitro and in vivo. Further mechanistic studies showed that FDI-6 also inhibited the expression of FOXM1 and PARP1/2 to sensitize BRCA-proficient TNBC cells to Olaparib. DCLK1 expression was significantly promoted by Olaparib. DCLK1 is a microtubule-associated oncoprotein that regulates cell apoptosis and DNA repair to promote tumorigenesis [36]. Therefore, the overexpression of DCLK1 contributes to the generation of adaptive resistance. Silencing FOXM1 expression or inhibiting FOXM1 function significantly inhibited the expression of DCLK1 to impair Olaparib-induced DCLK1 expression.

In response to DNA damage, cells trigger complex molecular reactions, such as detecting DNA damage, arresting cell cycle progression for DNA repair, and initiating DNA repair pathways [37]. Olaparib significantly increased the expression of CDK6, CCND1, CDK1, CCNA1, CCNB1, and CDC25B to accelerate cell cycle progression and arrested cell cycle at S and G_2_ phases. Olaparib induces the accumulation of DSBs, which are critical DNA lesions [9, 10]. HR-mediated repair triggers precise repair of DNA DSBs [9, 10, 20]. HR is activated only in the S and G_2_ phases after DNA replication [33]. Thus, the arresting of cell cycle at the S and G_2_ phases induced by Olaparib would activate HR repair, which is not conducive to killing cancer cells [37]. FOXM1 is a critical regulator of the G_1_/S and G_2_/M cell cycle transitions, as well as mitosis [27]. FDI-6 has been reported to inhibit the expression of downstream target genes of FOXM1, such as CDC25B and CCNB1 [30]. We found that FDI-6 and FOXM1 shRNA impaired Olaparib-induced expression of CDK1, CCNA1, CCNB1, and CDC25B to inhibit the mitosis of cancer cells. Meanwhile, the inhibition of FOXM1 repressed the expression of CDK6, CCND1, CDK2, CCNE2, E2F2, and CDC25A to arrest the cell cycle at G_2_ phase, which blocks the promotion of cancer-cell mitosis [27].

In addition to cell cycle regulation, FOXM1 was implicated in the DNA damage response. XRCC1 and BRCA2 are the direct transcriptional targets of FOXM1 [28]. We found that inhibition of FOXM1 promoted Olaparib-induced DNA damage. Further mechanistic studies showed that Olaparib promoted the expression of PLK1, MDC1, BRCA1/2 and Rad51 and inhibited the expression of XRCC1/2. BRCA1/2, and Rad51 are directly involved in the HR repair pathway [10–12]. PLK1 plays a critical role in cell cycle progression and the HR repair [38]. PLK1 directly phosphorylates Rad51 to increase its interaction with Nbs1 to activate HR repair [38]. MDC1 is a tumor suppressor that interacts with Rad51 to facilitate HR repair [39]. XRCC1 is an essential scaffold protein for BER that interacts with multiple enzymatic components of DNA SSB repair to accelerate the repair of DNA SSBs [40]. PARP1 is required for the recruitment of XRCC1 to the correct position on DNA SSBs [40]. XRCC2 functions in HR-mediated DNA DSBs by enhancing the activity of Rad51 [41]. Olaparib-induced expression of PLK1, BRCA1/2, and Rad51 could promote HR repair. Inhibition of FOXM1 reverses Olaparib-induced promotion of HR repair and promotes Olaparib-induced XRCC1 inhibition to impair BER of SSBs.

**Fig. 7:**
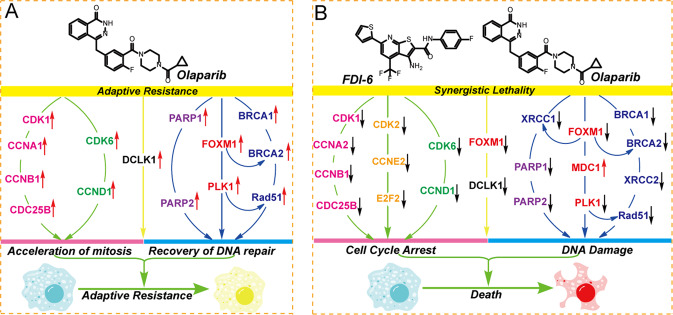
Mechanism for the synergistic effects of FDI-6 and Olaparib on the treatment of TNBC cells in vitro and in vivo. **A** Long-term treatment of Olaparib induces adaptive response of TNBC cells by promoting cancer cell mitosis and DNA repair. **B** FOXM1 repression inhibits the expression of genes involved in cell cycle progression and DNA damage repair to sensitize TNBC cells to Olaparib.

In summary, we clarified the mechanism for the synergistic effect of FDI-6 and Olaparib on the growth of BRCA-proficient TNBC cells in vitro and in vivo. After a long period of treatment, Olaparib increases the expression of DCLK1, PARP1/2, FOXM1, and other genes to induce the recovery of DNA repair and acceleration of cell cycle progression, which causes the generation of adaptive resistance (Fig. 7A). For cell cycle, Olaparib increases the expression of CDK6 and CCND1 to promote the G_1_/S transition and causes the overexpression of CDK1, CCNA1, CCNB1, and CDC25B to accelerate cell mitosis. For DNA repair, Olaparib promotes the expression of PARP1/2 to recover the repair of DNA SSBs and induces the expression of PLK1, XRCC2, BRCA1/2, and Rad51 to promote the HR repair of DNA DSBs (Fig. 7A). FDI-6 inhibits the function and expression of FOXM1 and sensitizes TNBC cells to Olaparib. FDI-6 and Olaparib synergistically inhibit the expression of CDK6, CCND1, CDK1, CCNA2, CCNB1 and CDC25B to block cell cycle progression. Meanwhile, FDI-6 and Olaparib synergistically inhibit DNA SSB repair by inhibiting the expression of XRCC1 and PARP1/2. They also synergistically inhibit HR repair by decreasing the expression of DCLK1, PLK1, XRCC1, BRCA1/2, and Rad51 and increasing the expression of MDC1 (Fig. 7B). We believe that targeting FOXM1 and PARP1 in combination is a promising strategy for the treatment of TNBCs.

## Supplementary information

Supplementary information

Supplementary Figure Legend

Supplementary Figure 1

Supplementary Figure 2

Supplementary Figure 3

Supplementary Figure 4

Supplementary Figure 5

Supplementary Figure 6

Supplementary Figure 7

Supplementary Figure 8

Supplementary Figure 9

Supplementary Figure 10

Supplementary Figure 11

Supplementary Figure 12

Supplementary Figure 13

Supplementary Figure 14

Supplementary Table 1

Supplementary Table 2

Supplementary Table 3

Supplementary Table 4

Supplementary Table 5

Supplementary Table 6

Author contribution

checklist

## Data Availability

The datasets generated and/or analyzed during the current study are available from the corresponding author on reasonable request.
