# Supplementary material for: FDI-6 inhibits the expression and function of FOXM1 to sensitize BRCA-proficient triple-negative breast cancer cells to Olaparib by regulating cell cycle progression and DNA damage repair
Source: Cell Death Dis. 2021 Dec 8;12(12):1138. doi: 10.1038/s41419-021-04434-9 (PMC8654856; doi:10.1038/s41419-021-04434-9)
Supplement: Supplementary file 1 — Supplementary information [file 41419_2021_4434_MOESM1_ESM.doc]

**Supplemental data**

for

FDI-6 inhibits the expression and function of FOXM1 to sensitize BRCA-proficient triple-negative breast cancer cells to Olaparib by regulating cell cycle progression and DNA damage repair

**Running title:** FOXM1 inhibition sensitizes TNBC to Olaparib

Shu-Ping Wang^1,§,*^, Shi-Qi Wu^1,§^, Shi-Hui Huang^1^, Yi-Xuan Tang^1^, Liu-Qiong Meng^1^, Feng Liu^1^, Qi-Hua Zhu^2^, Yun-Gen Xu^1,2,*^

^1^State Key Laboratory of Natural Medicines and Jiangsu Key Laboratory of Drug Design and Optimization, China Pharmaceutical University, 211198 Nanjing, China.

^2^Jiangsu Key Laboratory of Drug Design and Optimization, Department of Medicinal Chemistry, China Pharmaceutical University, Nanjing 210009, China.

^§^The authors contributed equally: Shu-Ping Wang, Shi-Qi Wu.

Corresponding author: Shu-Ping Wang & Yun-Gen Xu

E-mail: [wangsp16@126.com](mailto:wangsp16@126.com) (SP. Wang), [xyg@cpu.edu.cn](mailto:xyg@cpu.edu.cn) (YG. Xu)

1. **Supplemental Tables**

**Supplemental Table 1. Sequences for shRNA.**

| Name | Sequence |
| --- | --- |
| NC shRNA | CCGGCATTCTCCGAACGTGTCACGTCTCGAGACGTGACACGTTCGGAGAATTTTTG |
| FOXM1 shRNA1 | CCGGCAGCTGGGATCAAGATTATTACTCGAGTAATAATCTTGATCCCAGCTGTTTTTG |
| FOXM1 shRNA2 | CCGGGCGGCCACCCTACTCTTACATCTCGAGATGTAAGAGTAGGGTGGCCGCTTTTTG |
| FOXM1 shRNA3 | CCGGCAGGCTGCACTATCAACAATACTCGAGTATTGTTGATAGTGCAGCCTGTTTTTG |

**Supplemental Table 2.** Primer sequences for genes in Q-PCR.

| **Name** | **Sense (5’-3’)** | **Antisense (5’-3’)** |
| --- | --- | --- |
| PARP1 | ACACAATGCGTATGACTTGGA | CCGTGCCACAGCAATCTTCG |
| PARP2 | ACTATGCCACCAATACTCAGG | CTGTACCCGAAGATCTAGCTG |
| FOXM1 | ACCCAAACCAGCTATGATGCC | TCTCCCGTTTCTGCTCGCAAA |
| DCLK1 | GCAAAGAGCACATGATCCAGA | AGTTCAGTTGGCACATCCATC |
| MDC1 | TTGCAGCTTCCAGACAACAGGT | TTGGTCTCCTGGTATTGCCCTA |
| XRCC1 | CTCAAGGCAGACACTTACCGAA | AGCACCTCCACGAAAGCTGA |
| XRCC2 | CCTTTTGATTTTGGATAGCCT | GCGATAGTCATTTACAAGCTTC |
| PLK1 | GTACCTGCACCGAAACCGAGT | CCTCTCCCCGTCATATTCGACT |
| BRCA1 | TACTAGGCATAGCACCGTTG | ATGCCTTTGCCAATATTACCTG |
| BRCA2 | TGAAATTAAACGGAAGTTTGC | GAATAAAAGCCCCTAAACCC |
| Rad51 | CCCATTTCACGGTTAGAGCA | CTTTGGCTTCACTAATTCCCT |
| Rad52 | TTGCCACCAGAAACCACAAGC | ATTCCCAGTTTCCTGTTGTGC |
| CDK15 | ATAAATGGACAACTAGTGGCTT | ATTGGCATGTTTCAAACCCTT |
| CDC25A | CCTCCGAGTCAACAGATTCAGG | CTTCAGAGCTGGACTACATCCC |
| CDC25B | ACGCACCTATCCCTGTCTCG | TTCAAACGTCTGCTCCGCCAT |
| CDK1 | ATTTGGAGTATAGGCACCAT | GCCACACTTCATTATTGGGA |
| CCNB1 | TGAGAGCCATCCTAATTGACT | AATTATTCTGCATGAACCGAT |
| CCNA2 | CTGAAATAAGGCACAGACCCAA | ATACAGGGTCTCTGCTCGAA |
| CDK6 | ACTTTCTTCATTCACACCGAGT | AGTTTTATTTGTCCGCTGCT |
| CCND1 | CCATGAACTACCTGGACCGCTT | CTTAGAGGCCACGAACATGCAA |
| CDK2 | GCTTTCTGCCATTCTCATCGG | TGGCTAGTCCAAAGTCTGC |
| CCNE2 | TCCAAGAGTTTGCTTACGTCA | TTTAGGAGCATCTTTAAGAGC |
| E2F2 | CGGCGCATCTATGACATCACCA | CAAACATTCCCCTGCCTACCC |
| GAPDH | GAAACTGTGGCGTGATGGC | CACCACTGACACGTTGGCAG |

**Supplemental Table 3.** The inhibition ratios of FDI-6 against 361 kinases.

| Kinase | % Enzyme Activity | | IC50(M) Control Cmpd | Control Cmpd ID |
| --- | --- | --- | --- | --- |
|  | FDI-6 (1.0 μM) | |  |  |
|  | Data 1 | Data 2 |  |  |
| ABL1 | 105.01 | 102.85 | 7.56E-08 | STAUROSPORINE |
| ABL2/ARG | 113.72 | 112.99 | 2.98E-08 | STAUROSPORINE |
| ACK1 | 107.11 | 106.77 | 6.22E-08 | STAUROSPORINE |
| AKT1 | 98.68 | 92.74 | 2.35E-09 | STAUROSPORINE |
| AKT2 | 102.88 | 100.98 | 1.75E-08 | STAUROSPORINE |
| AKT3 | 114.11 | 106.61 | 3.25E-09 | STAUROSPORINE |
| ALK | 90.18 | 85.13 | 2.36E-09 | STAUROSPORINE |
| ALK1/ACVRL1 | 111.32 | 110.32 | 2.56E-08 | LDN193189 |
| ALK2/ACVR1 | 113.86 | 113.86 | 3.12E-08 | LDN193189 |
| ALK3/BMPR1A | 117.76 | 115.02 | 4.85E-08 | LDN193189 |
| ALK4/ACVR1B | 92.32 | 90.85 | 2.17E-07 | LDN193189 |
| ALK5/TGFBR1 | 108.13 | 106.94 | 5.19E-07 | LDN193189 |
| ALK6/BMPR1B | 100.57 | 100.36 | 1.02E-08 | LDN193189 |
| ARAF | 109.77 | 105.87 | 2.40E-08 | GW5074 |
| ARK5/NUAK1 | 90.82 | 89.44 | 6.61E-10 | STAUROSPORINE |
| ASK1/MAP3K5 | 95.06 | 94.96 | 1.53E-08 | STAUROSPORINE |
| AURORA A | 115.24 | 114.10 | 2.06E-09 | STAUROSPORINE |
| AURORA B | 103.28 | 100.47 | 2.31E-08 | STAUROSPORINE |
| AURORA C | 115.06 | 114.13 | 6.16E-09 | STAUROSPORINE |
| AXL | 102.52 | 101.95 | 4.02E-09 | STAUROSPORINE |
| BLK | 89.95 | 88.55 | 1.28E-09 | STAUROSPORINE |
| BMPR2 | 100.60 | 94.16 | 3.60E-07 | STAUROSPORINE |
| BMX/ETK | 91.00 | 90.82 | 5.21E-09 | STAUROSPORINE |
| BRAF | 83.23 | 80.32 | 4.41E-09 | GW5074 |
| BRK | 84.39 | 83.51 | 3.86E-07 | STAUROSPORINE |
| BRSK1 | 90.26 | 90.05 | 5.86E-10 | STAUROSPORINE |
| BRSK2 | 96.27 | 95.35 | 1.03E-09 | STAUROSPORINE |
| BTK | 95.02 | 89.70 | 1.57E-08 | STAUROSPORINE |
| C-KIT | 87.36 | 86.72 | 7.41E-10 | STAUROSPORINE |
| C-MER | 91.58 | 90.06 | 1.35E-08 | STAUROSPORINE |
| C-MET | 70.86 | 70.81 | 1.26E-07 | STAUROSPORINE |
| C-SRC | 92.88 | 91.68 | 1.83E-09 | STAUROSPORINE |
| CAMK1A | 105.50 | 104.70 | 3.30E-09 | STAUROSPORINE |
| CAMK1b | 102.13 | 101.96 | 4.70E-09 | STAUROSPORINE |
| CAMK1d | 105.15 | 103.58 | 6.56E-10 | STAUROSPORINE |
| CAMK1G | 102.12 | 100.64 | 5.75E-09 | STAUROSPORINE |
| CAMK2a | 98.37 | 94.57 | 7.97E-11 | STAUROSPORINE |
| CAMK2B | 109.96 | 108.55 | 7.98E-11 | STAUROSPORINE |
| CAMK2D | 92.10 | 89.10 | 5.54E-11 | STAUROSPORINE |
| CAMK2G | 84.35 | 81.41 | 1.86E-10 | STAUROSPORINE |
| CAMK4 | 77.24 | 76.97 | 9.44E-08 | STAUROSPORINE |
| CAMKK1 | 103.90 | 102.05 | 7.92E-08 | STAUROSPORINE |
| CAMKK2 | 72.06 | 65.75 | 2.97E-08 | STAUROSPORINE |
| CDC7/DBF4 | 88.56 | 87.34 | 4.72E-08 | STAUROSPORINE |
| CDK1/CYCLIN A | 115.15 | 111.88 | 6.02E-09 | STAUROSPORINE |
| CDK1/CYCLIN B | 92.79 | 91.32 | 2.46E-09 | STAUROSPORINE |
| CDK1/CYCLIN E | 87.05 | 86.79 | 3.39E-09 | STAUROSPORINE |
| CDK14/CYCLIN Y | 91.44 | 90.64 | 1.38E-07 | STAUROSPORINE |
| CDK16/CYCLIN Y | 95.12 | 93.40 | 2.22E-08 | STAUROSPORINE |
| CDK17/CYCLIN Y | 104.14 | 103.27 | 1.68E-08 | STAUROSPORINE |
| CDK18/CYCLIN Y | 88.97 | 84.92 | 1.77E-08 | STAUROSPORINE |
| CDK19/CYCLIN C | 97.34 | 95.76 | 2.78E-07 | STAUROSPORINE |
| CDK2/CYCLIN A | 122.08 | 121.92 | 7.96E-10 | STAUROSPORINE |
| CDK2/CYCLIN A1 | 104.31 | 104.22 | 1.69E-09 | STAUROSPORINE |
| CDK2/CYCLIN E | 98.80 | 97.73 | 2.24E-09 | STAUROSPORINE |
| CDK2/CYCLIN E2 | 94.27 | 92.28 | 2.66E-09 | STAUROSPORINE |
| CDK2/CYCLIN O | 98.12 | 95.43 | 1.35E-09 | STAUROSPORINE |
| CDK3/CYCLIN E | 95.00 | 94.49 | 2.56E-09 | STAUROSPORINE |
| CDK3/CYCLIN E2 | 103.86 | 100.38 | 2.83E-09 | STAUROSPORINE |
| CDK4/CYCLIN D1 | 97.54 | 94.90 | 1.55E-08 | STAUROSPORINE |
| CDK4/CYCLIN D3 | 81.13 | 80.11 | 1.63E-08 | STAUROSPORINE |
| CDK5/P25 | 100.01 | 97.18 | 1.63E-09 | STAUROSPORINE |
| CDK5/P35 | 102.69 | 102.25 | 2.19E-09 | STAUROSPORINE |
| CDK6/CYCLIN D1 | 98.95 | 96.46 | 4.05E-09 | STAUROSPORINE |
| CDK6/CYCLIN D3 | 87.26 | 85.95 | 2.41E-08 | STAUROSPORINE |
| CDK7/CYCLIN H | 106.10 | 102.43 | 6.15E-08 | STAUROSPORINE |
| CDK8/CYCLIN C | 108.26 | 107.11 | 2.07E-10 | STAUROSPORINE |
| CDK9/CYCLIN K | 95.01 | 89.61 | 2.09E-08 | STAUROSPORINE |
| CDK9/cyclin T1 | 86.24 | 84.88 | 4.72E-09 | STAUROSPORINE |
| CDK9/CYCLIN T2 | 104.22 | 102.18 | 3.94E-09 | STAUROSPORINE |
| CHK1 | 95.78 | 93.23 | 6.24E-10 | STAUROSPORINE |
| CHK2 | 98.03 | 97.12 | 6.55E-09 | STAUROSPORINE |
| CK1A1 | 98.56 | 96.07 | 4.41E-06 | STAUROSPORINE |
| CK1A1L | 83.19 | 82.14 | 2.28E-06 | STAUROSPORINE |
| CK1D | 82.28 | 80.11 | 2.17E-07 | D4476 |
| CK1EPSILON | 89.98 | 89.86 | 2.82E-07 | D4476 |
| CK1G2 | 105.55 | 104.71 | 3.78E-06 | STAUROSPORINE |
| CK1G3 | 98.45 | 97.99 | 1.90E-06 | STAUROSPORINE |
| CK2A | 82.23 | 82.02 | 1.95E-07 | GW5074 |
| CK2A2 | 125.45 | 123.06 | 1.22E-06 | STAUROSPORINE |
| CLK1 | 102.10 | 100.22 | 1.03E-08 | STAUROSPORINE |
| CLK2 | 97.17 | 96.80 | 4.58E-09 | STAUROSPORINE |
| CLK4 | 108.59 | 105.58 | 7.51E-08 | STAUROSPORINE |
| COT1/MAP3K8 | 77.53 | 75.94 | 8.70E-06 | RO-31-8220 |
| CSK | 93.31 | 92.44 | 1.44E-08 | STAUROSPORINE |
| CTK/MATK | 90.42 | 86.92 | 1.45E-07 | STAUROSPORINE |
| DAPK1 | 104.13 | 103.46 | 1.59E-08 | STAUROSPORINE |
| DAPK2 | 102.55 | 100.65 | 1.12E-08 | STAUROSPORINE |
| DCAMKL1 | 93.59 | 92.91 | 1.19E-07 | STAUROSPORINE |
| DCAMKL2 | 90.25 | 87.49 | 9.80E-09 | STAUROSPORINE |
| DDR1 | 103.79 | 103.06 | 8.54E-09 | STAUROSPORINE |
| DDR2 | 85.61 | 84.84 | 4.49E-10 | STAUROSPORINE |
| DMPK | 87.35 | 86.45 | 2.41E-08 | STAUROSPORINE |
| DMPK2 | 105.65 | 103.30 | 5.10E-10 | STAUROSPORINE |
| DRAK1/STK17A | 91.09 | 87.26 | 4.18E-08 | STAUROSPORINE |
| DYRK1/DYRK1A | 77.94 | 72.45 | 3.50E-09 | STAUROSPORINE |
| DYRK1B | 95.99 | 95.62 | 2.00E-09 | STAUROSPORINE |
| DYRK2 | 108.45 | 108.35 | 2.59E-07 | STAUROSPORINE |
| DYRK3 | 96.40 | 96.10 | 5.29E-08 | STAUROSPORINE |
| DYRK4 | 90.74 | 88.99 | 3.16E-06 | GW5074 |
| EGFR | 82.47 | 80.76 | 1.39E-07 | STAUROSPORINE |
| EPHA1 | 73.82 | 73.43 | 7.88E-08 | STAUROSPORINE |
| EPHA2 | 112.68 | 106.34 | 6.55E-08 | STAUROSPORINE |
| EPHA3 | 95.14 | 94.95 | 2.75E-08 | STAUROSPORINE |
| EPHA4 | 119.77 | 116.59 | 3.59E-08 | STAUROSPORINE |
| EPHA5 | 100.54 | 97.99 | 6.01E-08 | STAUROSPORINE |
| EPHA6 | 94.77 | 94.30 | 2.79E-08 | STAUROSPORINE |
| EPHA7 | 87.64 | 85.33 | 5.46E-08 | STAUROSPORINE |
| EPHA8 | 106.95 | 104.50 | 1.31E-07 | STAUROSPORINE |
| EPHB1 | 110.12 | 110.07 | 9.15E-08 | STAUROSPORINE |
| EPHB2 | 101.45 | 100.93 | 1.64E-07 | STAUROSPORINE |
| EPHB3 | 93.35 | 93.30 | 1.43E-06 | STAUROSPORINE |
| EPHB4 | 89.38 | 87.48 | 1.99E-07 | STAUROSPORINE |
| ERBB2/HER2 | 107.00 | 105.60 | 1.40E-07 | STAUROSPORINE |
| ERBB4/HER4 | 89.24 | 88.22 | 3.47E-07 | STAUROSPORINE |
| ERK1 | 85.60 | 84.62 | 5.16E-09 | SCH772984 |
| ERK2/MAPK1 | 93.88 | 91.75 | 1.35E-09 | SCH772984 |
| ERK5/MAPK7 | 92.66 | 92.58 | 2.23E-05 | STAUROSPORINE |
| ERK7/MAPK15 | 103.27 | 103.10 | 1.18E-08 | STAUROSPORINE |
| ERN1/IRE1 | 72.70 | 70.11 | 7.45E-08 | STAUROSPORINE |
| ERN2/IRE2 | 111.90 | 109.30 | 6.05E-08 | STAUROSPORINE |
| FAK/PTK2 | 93.27 | 92.77 | 9.99E-09 | STAUROSPORINE |
| FER | 86.52 | 86.49 | 3.20E-10 | STAUROSPORINE |
| FES/FPS | 117.74 | 115.59 | 6.49E-10 | STAUROSPORINE |
| FGFR1 | 94.87 | 92.77 | 4.40E-09 | STAUROSPORINE |
| FGFR2 | 96.57 | 94.73 | 2.57E-09 | STAUROSPORINE |
| FGFR3 | 78.00 | 77.48 | 2.19E-08 | STAUROSPORINE |
| FGFR4 | 107.37 | 96.63 | 5.29E-08 | STAUROSPORINE |
| FGR | 121.76 | 121.59 | 6.38E-10 | STAUROSPORINE |
| FLT1/VEGFR1 | 84.80 | 84.48 | 8.69E-09 | STAUROSPORINE |
| FLT3 | 93.23 | 88.71 | 5.65E-09 | STAUROSPORINE |
| FLT4/VEGFR3 | 93.52 | 93.34 | 2.70E-09 | STAUROSPORINE |
| FMS | 114.63 | 111.56 | 1.17E-09 | STAUROSPORINE |
| FRK/PTK5 | 84.88 | 80.61 | 2.79E-08 | STAUROSPORINE |
| FYN | 94.83 | 93.50 | 8.22E-10 | STAUROSPORINE |
| GCK/MAP4K2 | 101.22 | 98.45 | 4.62E-10 | STAUROSPORINE |
| GLK/MAP4K3 | 109.29 | 108.90 | 1.79E-10 | STAUROSPORINE |
| GRK1 | 85.41 | 82.39 | 5.46E-08 | STAUROSPORINE |
| GRK2 | 96.66 | 95.01 | 1.16E-06 | STAUROSPORINE |
| GRK3 | 93.08 | 91.08 | 6.61E-07 | STAUROSPORINE |
| GRK4 | 117.24 | 116.32 | 1.50E-07 | STAUROSPORINE |
| GRK5 | 107.65 | 103.88 | 6.70E-08 | STAUROSPORINE |
| GRK6 | 118.62 | 117.74 | 6.04E-08 | STAUROSPORINE |
| GRK7 | 74.81 | 74.11 | 3.72E-09 | STAUROSPORINE |
| GSK3a | 99.67 | 99.20 | 5.05E-09 | STAUROSPORINE |
| GSK3b | 94.70 | 93.83 | 5.03E-09 | STAUROSPORINE |
| HASPIN | 104.63 | 102.37 | 2.28E-08 | STAUROSPORINE |
| HCK | 103.71 | 103.38 | 1.77E-09 | STAUROSPORINE |
| HGK/MAP4K4 | 70.74 | 62.24 | 1.02E-09 | STAUROSPORINE |
| HIPK1 | 102.94 | 101.94 | 1.98E-06 | RO-31-8220 |
| HIPK2 | 107.57 | 103.38 | 3.48E-07 | STAUROSPORINE |
| HIPK3 | 96.05 | 91.65 | 1.92E-06 | STAUROSPORINE |
| HIPK4 | 101.48 | 100.64 | 6.00E-07 | STAUROSPORINE |
| HPK1/MAP4K1 | 87.35 | 85.33 | 6.58E-08 | RO-31-8220 |
| IGF1R | 82.06 | 80.72 | 7.64E-08 | STAUROSPORINE |
| IKKA/CHUK | 85.54 | 84.35 | 1.14E-07 | STAUROSPORINE |
| IKKB/IKBKB | 79.96 | 79.13 | 3.17E-07 | STAUROSPORINE |
| IKKE/IKBKE | 97.40 | 96.55 | 4.22E-10 | STAUROSPORINE |
| IR | 103.70 | 102.24 | 2.74E-08 | STAUROSPORINE |
| IRAK1 | 83.24 | 81.10 | 6.35E-08 | STAUROSPORINE |
| IRAK4 | 85.03 | 84.79 | 2.43E-09 | STAUROSPORINE |
| IRR/INSRR | 98.36 | 98.17 | 1.70E-08 | STAUROSPORINE |
| ITK | 117.39 | 117.35 | 2.15E-08 | STAUROSPORINE |
| JAK1 | 94.25 | 93.70 | 8.48E-10 | STAUROSPORINE |
| JAK2 | 92.76 | 92.29 | 2.96E-10 | STAUROSPORINE |
| JAK3 | 100.76 | 100.30 | 1.55E-10 | STAUROSPORINE |
| JNK1 | 92.84 | 91.12 | 8.13E-07 | STAUROSPORINE |
| JNK2 | 114.51 | 110.72 | 2.92E-06 | STAUROSPORINE |
| JNK3 | 70.10 | 66.15 | 4.77E-08 | JNKI VIII |
| KDR/VEGFR2 | 95.23 | 94.97 | 1.99E-08 | STAUROSPORINE |
| KHS/MAP4K5 | 79.35 | 78.47 | 2.75E-10 | STAUROSPORINE |
| KSR1 | 97.48 | 97.15 | 1.32E-05 | STAUROSPORINE |
| KSR2 | 82.54 | 80.64 | 3.19E-06 | STAUROSPORINE |
| LATS1 | 111.49 | 109.80 | 2.26E-08 | STAUROSPORINE |
| LATS2 | 95.17 | 90.60 | 6.73E-09 | STAUROSPORINE |
| LCK | 107.97 | 102.23 | 2.68E-09 | STAUROSPORINE |
| LCK2/ICK | 97.48 | 97.05 | 2.47E-08 | STAUROSPORINE |
| LIMK1 | 96.74 | 96.50 | 1.31E-09 | STAUROSPORINE |
| LIMK2 | 100.69 | 98.16 | 8.01E-08 | STAUROSPORINE |
| LKB1 | 88.43 | 87.29 | 5.82E-08 | STAUROSPORINE |
| LOK/STK10 | 104.05 | 102.81 | 1.04E-07 | RO-31-8220 |
| LRRK2 | 92.78 | 92.27 | 1.29E-08 | STAUROSPORINE |
| LYN | 114.37 | 112.85 | 1.66E-09 | STAUROSPORINE |
| LYN B | 133.34 | 132.30 | 5.64E-09 | STAUROSPORINE |
| MAK | 94.60 | 90.06 | 2.76E-08 | STAUROSPORINE |
| MAPKAPK2 | 75.11 | 74.45 | 1.50E-07 | STAUROSPORINE |
| MAPKAPK3 | 102.15 | 101.95 | 2.84E-06 | STAUROSPORINE |
| MARK1 | 97.50 | 95.69 | 1.09E-10 | STAUROSPORINE |
| MARK2/PAR-1Ba | 96.67 | 96.36 | 1.20E-10 | STAUROSPORINE |
| MARK3 | 99.87 | 97.42 | 1.24E-10 | STAUROSPORINE |
| MARK4 | 84.56 | 84.38 | 4.30E-10 | STAUROSPORINE |
| MAST3 | 102.52 | 101.12 | 1.08E-06 | STAUROSPORINE |
| MASTL | 96.00 | 91.87 | 3.38E-08 | STAUROSPORINE |
| MEK1 | 102.83 | 100.91 | 3.70E-08 | STAUROSPORINE |
| MEK2 | 107.09 | 107.09 | 7.30E-08 | STAUROSPORINE |
| MEK3 | 111.96 | 111.73 | 3.73E-08 | STAUROSPORINE |
| MEK5 | 97.67 | 97.07 | 6.35E-08 | STAUROSPORINE |
| MEKK1 | 114.31 | 113.53 | 8.28E-07 | STAUROSPORINE |
| MEKK2 | 93.49 | 93.01 | 7.89E-08 | STAUROSPORINE |
| MEKK3 | 83.89 | 82.10 | 2.92E-08 | STAUROSPORINE |
| MEKK6 | 102.69 | 102.48 | 5.58E-07 | STAUROSPORINE |
| MELK | 94.30 | 93.00 | 4.22E-10 | STAUROSPORINE |
| MINK/MINK1 | 93.65 | 90.56 | 2.69E-10 | STAUROSPORINE |
| MKK4 | 98.40 | 95.10 | 3.06E-06 | STAUROSPORINE |
| MKK6 | 107.78 | 103.29 | 2.01E-08 | STAUROSPORINE |
| MKK7 | 99.14 | 97.69 | 3.12E-06 | STAUROSPORINE |
| MLCK/MYLK | 104.45 | 103.34 | 3.26E-08 | STAUROSPORINE |
| MLCK2/MYLK2 | 88.30 | 85.29 | 1.64E-08 | STAUROSPORINE |
| MLK1/MAP3K9 | 89.37 | 84.44 | 5.16E-10 | STAUROSPORINE |
| MLK2/MAP3K10 | 113.82 | 105.81 | 3.50E-09 | STAUROSPORINE |
| MLK3/MAP3K11 | 106.73 | 103.96 | 2.08E-09 | STAUROSPORINE |
| MLK4 | 86.85 | 85.86 | 1.92E-06 | STAUROSPORINE |
| MNK1 | 100.40 | 99.90 | 1.12E-07 | STAUROSPORINE |
| MNK2 | 95.13 | 90.39 | 1.90E-08 | STAUROSPORINE |
| MRCKa/CDC42BPA | 104.98 | 104.54 | 7.57E-09 | STAUROSPORINE |
| MRCKB/CDC42BPB | 100.93 | 98.82 | 3.85E-09 | STAUROSPORINE |
| MSK2/RPS6KA4 | 103.80 | 103.59 | 1.76E-09 | STAUROSPORINE |
| MSSK1/STK23 | 77.52 | 74.50 | 1.53E-06 | STAUROSPORINE |
| MST1/STK4 | 110.31 | 106.66 | 1.53E-09 | STAUROSPORINE |
| MST2/STK3 | 98.91 | 98.61 | 4.04E-09 | STAUROSPORINE |
| MST3/STK24 | 95.36 | 94.77 | 7.52E-09 | STAUROSPORINE |
| MST4 | 94.00 | 92.74 | 7.66E-09 | STAUROSPORINE |
| MUSK | 95.04 | 91.17 | 4.68E-09 | STAUROSPORINE |
| MYLK3 | 100.44 | 97.99 | 8.23E-08 | STAUROSPORINE |
| MYLK4 | 78.58 | 77.86 | 3.32E-08 | STAUROSPORINE |
| MYO3A | 88.57 | 86.07 | 2.32E-08 | STAUROSPORINE |
| MYO3b | 100.37 | 97.71 | 8.37E-09 | STAUROSPORINE |
| NEK1 | 104.55 | 102.63 | 2.11E-08 | STAUROSPORINE |
| NEK11 | 104.73 | 103.70 | 1.40E-06 | STAUROSPORINE |
| NEK2 | 104.22 | 103.62 | 2.12E-07 | STAUROSPORINE |
| NEK3 | 104.20 | 92.43 | 1.01E-07 | JNK-IN-7 |
| NEK4 | 121.09 | 120.79 | 1.41E-07 | STAUROSPORINE |
| NEK5 | 83.41 | 83.26 | 2.77E-08 | STAUROSPORINE |
| NEK6 | 86.81 | 85.59 | 1.27E-05 | PKR INHIBITOR |
| NEK7 | 90.76 | 90.51 | 9.71E-06 | PKR INHIBITOR |
| NEK9 | 85.46 | 84.27 | 1.01E-07 | STAUROSPORINE |
| NIM1 | 86.29 | 85.46 | 1.58E-07 | STAUROSPORINE |
| NLK | 92.56 | 90.90 | 5.12E-08 | STAUROSPORINE |
| OSR1/OXSR1 | 96.15 | 95.79 | 7.63E-08 | STAUROSPORINE |
| P38A/MAPK14 | 86.54 | 83.10 | 1.22E-08 | SB202190 |
| P38B/MAPK11 | 90.28 | 88.83 | 2.14E-08 | SB202190 |
| P38D/MAPK13 | 115.90 | 111.11 | 4.62E-07 | STAUROSPORINE |
| P38G | 102.64 | 100.67 | 3.51E-07 | STAUROSPORINE |
| p70S6Kb/RPS6KB2 | 104.56 | 101.41 | 3.26E-09 | STAUROSPORINE |
| PAK1 | 99.84 | 98.41 | 1.49E-10 | STAUROSPORINE |
| PAK2 | 108.67 | 108.19 | 1.36E-09 | STAUROSPORINE |
| PAK3 | 103.80 | 102.45 | 1.68E-10 | STAUROSPORINE |
| PAK4 | 106.24 | 105.53 | 5.44E-09 | STAUROSPORINE |
| PAK5 | 90.13 | 89.74 | 3.68E-09 | STAUROSPORINE |
| PAK6 | 100.79 | 100.55 | 7.93E-09 | STAUROSPORINE |
| PBK/TOPK | 83.47 | 77.58 | 4.27E-08 | STAUROSPORINE |
| PDGFRA | 99.96 | 97.37 | 1.33E-09 | STAUROSPORINE |
| PDGFRB | 91.00 | 86.69 | 1.48E-09 | STAUROSPORINE |
| PDK1/PDPK1 | 101.46 | 98.38 | 1.26E-09 | STAUROSPORINE |
| PHKG1 | 102.15 | 100.30 | 1.63E-09 | STAUROSPORINE |
| PHKG2 | 102.92 | 100.85 | 1.03E-09 | STAUROSPORINE |
| PIM1 | 103.78 | 99.89 | 6.03E-09 | STAUROSPORINE |
| PIM2 | 89.81 | 89.16 | 2.81E-08 | STAUROSPORINE |
| PIM3 | 87.05 | 86.34 | 9.46E-11 | STAUROSPORINE |
| PKA | 78.38 | 76.99 | 2.37E-09 | STAUROSPORINE |
| PKACB | 92.27 | 92.06 | 1.46E-09 | STAUROSPORINE |
| PKACG | 87.03 | 83.12 | 3.29E-09 | STAUROSPORINE |
| PKCa | 113.49 | 112.06 | 3.34E-10 | STAUROSPORINE |
| PKCb1 | 100.45 | 98.96 | 2.34E-09 | STAUROSPORINE |
| PKCb2 | 100.28 | 98.57 | 1.62E-09 | STAUROSPORINE |
| PKCD | 96.95 | 96.45 | 1.79E-10 | STAUROSPORINE |
| PKCEPSILON | 96.50 | 94.15 | 3.28E-10 | STAUROSPORINE |
| PKCETA | 105.23 | 100.47 | 3.59E-10 | STAUROSPORINE |
| PKCG | 92.75 | 91.54 | 2.00E-10 | STAUROSPORINE |
| PKCIOTA | 89.10 | 88.41 | 1.05E-08 | STAUROSPORINE |
| PKCMU/PRKD1 | 101.25 | 98.08 | 1.95E-09 | STAUROSPORINE |
| PKCTHETA | 88.19 | 86.17 | 3.24E-09 | STAUROSPORINE |
| PKCZETA | 99.15 | 96.65 | 6.51E-08 | STAUROSPORINE |
| PKD2/PRKD2 | 87.48 | 85.09 | 1.55E-09 | STAUROSPORINE |
| PKG1A | 83.29 | 80.68 | 2.66E-09 | STAUROSPORINE |
| PKG1B | 85.10 | 81.94 | 1.32E-09 | STAUROSPORINE |
| PKG2/PRKG2 | 94.99 | 89.60 | 2.24E-09 | STAUROSPORINE |
| PKN1/PRK1 | 85.71 | 84.09 | 2.36E-10 | STAUROSPORINE |
| PKN2/PRK2 | 85.02 | 83.20 | 1.44E-09 | STAUROSPORINE |
| PKN3/PRK3 | 97.70 | 96.13 | 8.97E-09 | STAUROSPORINE |
| PLK1 | 93.25 | 92.68 | 2.05E-07 | STAUROSPORINE |
| PLK2 | 79.10 | 77.82 | 1.63E-07 | STAUROSPORINE |
| PLK3 | 108.00 | 107.66 | 6.49E-09 | BI2536 |
| PLK4/SAK | 106.58 | 106.28 | 1.64E-08 | STAUROSPORINE |
| PRKX | 97.81 | 94.52 | 1.82E-09 | STAUROSPORINE |
| PYK2 | 104.09 | 102.03 | 9.69E-09 | STAUROSPORINE |
| RAF1 | 99.21 | 97.55 | 5.71E-09 | GW5074 |
| RET | 95.07 | 93.49 | 2.87E-09 | STAUROSPORINE |
| RIPK4 | 88.40 | 86.65 | 4.51E-07 | STAUROSPORINE |
| ROCK1 | 103.66 | 102.22 | 7.95E-10 | STAUROSPORINE |
| ROCK2 | 110.77 | 108.10 | 2.09E-09 | STAUROSPORINE |
| RON/MST1R | 98.21 | 97.11 | 3.46E-07 | STAUROSPORINE |
| ROS/ROS1 | 100.30 | 98.93 | 1.59E-10 | STAUROSPORINE |
| RSK1 | 87.77 | 87.66 | 1.95E-10 | STAUROSPORINE |
| RSK2 | 98.53 | 98.43 | 1.77E-10 | STAUROSPORINE |
| RSK3 | 94.47 | 87.43 | 2.96E-10 | STAUROSPORINE |
| RSK4 | 84.94 | 84.16 | 1.09E-10 | STAUROSPORINE |
| SBK1 | 92.43 | 91.68 | 8.74E-08 | STAUROSPORINE |
| SGK1 | 101.37 | 99.78 | 9.25E-09 | STAUROSPORINE |
| SGK2 | 111.68 | 106.39 | 1.84E-08 | STAUROSPORINE |
| SGK3/SGKL | 113.58 | 110.75 | 1.44E-07 | STAUROSPORINE |
| SIK1 | 94.85 | 94.81 | 3.96E-09 | STAUROSPORINE |
| SIK2 | 107.80 | 107.46 | 1.20E-09 | STAUROSPORINE |
| SIK3 | 98.67 | 98.41 | 1.87E-09 | STAUROSPORINE |
| SLK/STK2 | 65.49 | 55.28 | 1.24E-08 | STAUROSPORINE |
| SNARK/NUAK2 | 92.77 | 90.19 | 1.50E-09 | STAUROSPORINE |
| SNRK | 108.26 | 104.21 | 3.64E-08 | STAUROSPORINE |
| SRMS | 88.95 | 87.67 | 9.82E-06 | STAUROSPORINE |
| SRPK1 | 100.20 | 98.81 | 6.11E-08 | STAUROSPORINE |
| SRPK2 | 98.20 | 97.79 | 4.99E-07 | STAUROSPORINE |
| SSTK/TSSK6 | 96.42 | 93.72 | 2.22E-07 | STAUROSPORINE |
| STK16 | 97.53 | 92.24 | 1.80E-07 | STAUROSPORINE |
| STK21/CIT | 114.27 | 112.22 | 2.47E-07 | STAUROSPORINE |
| STK22D/TSSK1 | 97.77 | 95.50 | 1.11E-10 | STAUROSPORINE |
| STK25/YSK1 | 99.53 | 98.93 | 2.36E-09 | STAUROSPORINE |
| STK32B/YANK2 | 119.43 | 115.97 | 4.10E-08 | STAUROSPORINE |
| STK32C/YANK3 | 80.85 | 80.34 | 1.29E-07 | STAUROSPORINE |
| STK33 | 92.02 | 85.75 | 2.09E-08 | STAUROSPORINE |
| STK38/NDR1 | 89.32 | 88.25 | 6.56E-10 | STAUROSPORINE |
| STK38L/NDR2 | 101.43 | 99.30 | 1.12E-09 | STAUROSPORINE |
| STK39/STLK3 | 105.49 | 102.46 | 1.62E-08 | STAUROSPORINE |
| SYK | 107.11 | 101.51 | 5.98E-10 | STAUROSPORINE |
| TAK1 | 108.31 | 106.08 | 1.17E-07 | STAUROSPORINE |
| TAOK1 | 96.43 | 96.01 | 7.69E-10 | STAUROSPORINE |
| TAOK2/TAO1 | 91.47 | 90.25 | 5.94E-09 | STAUROSPORINE |
| TAOK3/JIK | 81.89 | 79.89 | 6.05E-09 | STAUROSPORINE |
| TBK1 | 106.73 | 99.41 | 9.66E-10 | STAUROSPORINE |
| TEC | 85.78 | 84.58 | 6.10E-08 | STAUROSPORINE |
| TESK1 | 91.54 | 91.51 | 1.83E-07 | STAUROSPORINE |
| TESK2 | 90.02 | 87.95 | 1.32E-05 | STAUROSPORINE |
| TGFBR2 | 90.07 | 87.81 | 1.14E-07 | LDN193189 |
| TIE2/TEK | 94.63 | 94.44 | 7.68E-08 | STAUROSPORINE |
| TLK1 | 101.33 | 99.95 | 4.75E-08 | STAUROSPORINE |
| TLK2 | 93.06 | 91.90 | 4.02E-09 | STAUROSPORINE |
| TNIK | 98.30 | 97.39 | 3.51E-10 | STAUROSPORINE |
| TNK1 | 77.76 | 76.28 | 4.96E-09 | STAUROSPORINE |
| TRKA | 99.14 | 98.41 | 3.05E-09 | STAUROSPORINE |
| TRKB | 91.34 | 88.49 | 4.47E-10 | STAUROSPORINE |
| TRKC | 99.81 | 99.68 | 2.08E-10 | STAUROSPORINE |
| TSSK2 | 93.60 | 93.45 | 6.52E-09 | STAUROSPORINE |
| TSSK3/STK22C | 93.97 | 92.92 | 5.98E-09 | STAUROSPORINE |
| TTBK1 | 109.75 | 105.17 | 1.63E-05 | SB202190 |
| TTBK2 | 96.30 | 95.07 | 4.19E-06 | SB202190 |
| TXK | 92.34 | 92.10 | 3.90E-08 | STAUROSPORINE |
| TYK1/LTK | 103.77 | 99.57 | 4.81E-08 | STAUROSPORINE |
| TYK2 | 82.12 | 80.86 | 4.02E-10 | STAUROSPORINE |
| TYRO3/SKY | 113.76 | 110.75 | 3.35E-09 | STAUROSPORINE |
| ULK1 | 87.32 | 83.81 | 6.32E-09 | STAUROSPORINE |
| ULK2 | 103.59 | 102.95 | 2.96E-09 | STAUROSPORINE |
| ULK3 | 90.10 | 89.65 | 3.75E-09 | STAUROSPORINE |
| VRK1 | 82.45 | 81.77 | 4.59E-07 | RO-31-8220 |
| VRK2 | 88.26 | 87.20 | 1.18E-05 | RO-31-8220 |
| WEE1 | 73.14 | 72.76 | 6.16E-08 | WEE-1 INHIBITOR |
| WNK1 | 93.38 | 92.23 | 1.98E-05 | STAUROSPORINE |
| WNK2 | 79.44 | 77.35 | 6.20E-06 | STAUROSPORINE |
| WNK3 | 100.27 | 99.93 | 1.43E-06 | WEE-1 INHIBITOR |
| YES/YES1 | 103.25 | 100.41 | 9.14E-10 | STAUROSPORINE |
| YSK4/MAP3K19 | 98.99 | 92.24 | 1.08E-08 | STAUROSPORINE |
| ZAK/MLTK | 101.85 | 101.12 | 1.41E-06 | GW5074 |
| ZAP70 | 100.24 | 99.07 | 1.36E-08 | STAUROSPORINE |
| ZIPK/DAPK3 | 101.36 | 100.39 | 6.28E-09 | STAUROSPORINE |

**Supplemental Table 4. DEGs in control vs FDI-6 treated group analyzed by RNA sequencing.**

| ID | Symbol | log_2_(fc) | P Value | FDR |
| --- | --- | --- | --- | --- |
| ENSG00000205923 | CEMP1 | 5.5046204 | 7.15E-05 | 0.000530735 |
| ENSG00000132207 | SLX1A | 5.4093909 | 0.000666232 | 0.003724187 |
| ENSG00000138395 | CDK15 | 3.1154772 | 1.38E-10 | 3.11E-09 |
| ENSG00000153404 | PLEKHG4B | 2.9385995 | 2.17E-05 | 0.000183443 |
| ENSG00000138622 | HCN4 | 2.7327161 | 1.69E-07 | 2.31E-06 |
| ENSG00000114812 | VIPR1 | 2.7202068 | 6.69E-169 | 2.92E-165 |
| ENSG00000140465 | CYP1A1 | 2.7147406 | 9.13E-115 | 2.39E-111 |
| ENSG00000184937 | WT1 | 2.6421064 | 0.004971273 | 0.020466638 |
| ENSG00000186628 | FSD2 | 2.4594316 | 0.009372494 | 0.034731019 |
| ENSG00000060566 | CREB3L3 | 2.428237 | 9.31E-07 | 1.08E-05 |
| ENSG00000103888 | CEMIP | 2.2983413 | 1.47E-07 | 2.02E-06 |
| ENSG00000138061 | CYP1B1 | 2.2829056 | 0 | 0 |
| ENSG00000197632 | SERPINB2 | 2.2672984 | 3.66E-60 | 2.28E-57 |
| ENSG00000189057 | FAM111B | 2.2576071 | 4.19E-31 | 5.90E-29 |
| ENSG00000121858 | TNFSF10 | 2.2307016 | 5.41E-44 | 1.51E-41 |
| ENSG00000268975 | MIA-RAB4B | 2.2209992 | 9.41E-05 | 0.00067697 |
| ENSG00000165495 | PKNOX2 | 2.1926451 | 0.013010983 | 0.045667505 |
| ENSG00000280893 | AC009133.6 | 2.103951 | 9.24E-06 | 8.65E-05 |
| ENSG00000092470 | WDR76 | 2.0990294 | 5.37E-19 | 3.17E-17 |
| ENSG00000148735 | PLEKHS1 | 2.0285692 | 0.0087332 | 0.032864343 |
| ENSG00000175322 | ZNF519 | 2.0204641 | 6.38E-06 | 6.19E-05 |
| ENSG00000143858 | SYT2 | 1.9919627 | 5.38E-09 | 9.52E-08 |
| ENSG00000118513 | MYB | 1.9886847 | 0.00397823 | 0.017065198 |
| ENSG00000085840 | ORC1 | 1.9686959 | 3.94E-14 | 1.38E-12 |
| ENSG00000198125 | MB | 1.9391748 | 0.003628047 | 0.015853935 |
| ENSG00000007968 | E2F2 | 1.9130167 | 3.03E-06 | 3.15E-05 |
| ENSG00000196611 | MMP1 | 1.8774116 | 5.00E-47 | 1.60E-44 |
| ENSG00000159307 | SCUBE1 | 1.8744691 | 0.001938357 | 0.009281992 |
| ENSG00000128408 | RIBC2 | 1.8008999 | 0.007645008 | 0.029342845 |
| ENSG00000187554 | TLR5 | 1.7454272 | 0.008238164 | 0.031280175 |
| ENSG00000100302 | RASD2 | 1.714132 | 3.43E-07 | 4.41E-06 |
| ENSG00000144354 | CDCA7 | 1.6619403 | 5.16E-25 | 5.32E-23 |
| ENSG00000162062 | TEDC2 | 1.6380087 | 0.002217765 | 0.010438945 |
| ENSG00000166106 | ADAMTS15 | 1.6343914 | 4.16E-38 | 8.38E-36 |
| ENSG00000185920 | PTCH1 | 1.6163796 | 1.35E-51 | 5.37E-49 |
| ENSG00000100297 | MCM5 | 1.6127744 | 4.16E-42 | 1.07E-39 |
| ENSG00000143603 | KCNN3 | 1.6088092 | 2.37E-12 | 6.65E-11 |
| ENSG00000198056 | PRIM1 | 1.5818262 | 3.45E-11 | 8.31E-10 |
| ENSG00000175305 | CCNE2 | 1.5754523 | 5.00E-27 | 5.54E-25 |
| ENSG00000164920 | OSR2 | 1.5687066 | 0.003604265 | 0.015771068 |
| ENSG00000169129 | AFAP1L2 | 1.5677552 | 1.25E-34 | 2.15E-32 |
| ENSG00000100628 | ASB2 | 1.556226 | 4.84E-73 | 4.53E-70 |
| ENSG00000114491 | UMPS | 1.5462054 | 0.000104794 | 0.00074499 |
| ENSG00000104738 | MCM4 | 1.5349083 | 2.52E-88 | 4.12E-85 |
| ENSG00000124171 | PARD6B | 1.5011848 | 2.06E-51 | 7.94E-49 |
| ENSG00000239732 | TLR9 | 1.4882865 | 1.45E-07 | 2.00E-06 |
| ENSG00000115687 | PASK | 1.4707469 | 1.32E-08 | 2.21E-07 |
| ENSG00000162654 | GBP4 | 1.4666425 | 5.98E-08 | 9.01E-07 |
| ENSG00000133056 | PIK3C2B | 1.4565141 | 2.45E-13 | 7.87E-12 |
| ENSG00000180616 | SSTR2 | 1.447459 | 0.000116134 | 0.000818885 |
| ENSG00000171408 | PDE7B | 1.4267705 | 1.64E-10 | 3.63E-09 |
| ENSG00000250644 | AC068580.4 | 1.4203889 | 1.04E-80 | 1.23E-77 |
| ENSG00000101412 | E2F1 | 1.4142383 | 1.05E-30 | 1.44E-28 |
| ENSG00000265763 | ZNF488 | 1.4095624 | 0.000193736 | 0.001289475 |
| ENSG00000104081 | BMF | 1.4010691 | 1.72E-39 | 3.81E-37 |
| ENSG00000147614 | ATP6V0D2 | 1.3953772 | 1.10E-09 | 2.15E-08 |
| ENSG00000109971 | HSPA8 | 1.3903778 | 2.16E-232 | 1.41E-228 |
| ENSG00000164379 | FOXQ1 | 1.3752913 | 1.00E-05 | 9.29E-05 |
| ENSG00000146678 | IGFBP1 | 1.3604565 | 1.74E-33 | 2.85E-31 |
| ENSG00000119714 | GPR68 | 1.3501051 | 9.43E-35 | 1.65E-32 |
| ENSG00000100479 | POLE2 | 1.3468981 | 1.09E-11 | 2.81E-10 |
| ENSG00000180884 | ZNF792 | 1.3440538 | 3.30E-12 | 9.09E-11 |
| ENSG00000131153 | GINS2 | 1.3331992 | 2.52E-06 | 2.68E-05 |
| ENSG00000241360 | PDXP | 1.3223362 | 5.44E-12 | 1.46E-10 |
| ENSG00000276043 | UHRF1 | 1.2958629 | 1.32E-46 | 4.12E-44 |
| ENSG00000089723 | OTUB2 | 1.289543 | 7.11E-40 | 1.61E-37 |
| ENSG00000184992 | BRI3BP | 1.2894196 | 2.35E-25 | 2.48E-23 |
| ENSG00000077942 | FBLN1 | 1.2797174 | 2.09E-07 | 2.80E-06 |
| ENSG00000167513 | CDT1 | 1.2771627 | 3.32E-13 | 1.05E-11 |
| ENSG00000136982 | DSCC1 | 1.2620322 | 2.07E-10 | 4.52E-09 |
| ENSG00000092853 | CLSPN | 1.2617209 | 1.58E-22 | 1.37E-20 |
| ENSG00000143476 | DTL | 1.2610729 | 1.21E-37 | 2.36E-35 |
| ENSG00000112118 | MCM3 | 1.2590209 | 1.39E-87 | 1.82E-84 |
| ENSG00000146966 | DENND2A | 1.2466178 | 1.48E-09 | 2.83E-08 |
| ENSG00000181544 | FANCB | 1.2386254 | 2.91E-06 | 3.04E-05 |
| ENSG00000270181 | BIVM-ERCC5 | 1.2374993 | 0.000989526 | 0.005238527 |
| ENSG00000162496 | DHRS3 | 1.2362893 | 4.24E-26 | 4.55E-24 |
| ENSG00000076003 | MCM6 | 1.2357205 | 2.95E-50 | 1.04E-47 |
| ENSG00000132646 | PCNA | 1.2257671 | 2.85E-39 | 6.02E-37 |
| ENSG00000180730 | SHISA2 | 1.2141036 | 3.18E-14 | 1.12E-12 |
| ENSG00000102312 | PORCN | 1.2068752 | 5.06E-25 | 5.26E-23 |
| ENSG00000135723 | FHOD1 | 1.2068246 | 1.56E-29 | 2.04E-27 |
| ENSG00000161939 | RNASEK-C17orf49 | 1.2063413 | 0.003568777 | 0.015652406 |
| ENSG00000204634 | TBC1D8 | 1.2051934 | 1.84E-19 | 1.14E-17 |
| ENSG00000172379 | ARNT2 | 1.1955508 | 1.16E-24 | 1.15E-22 |
| ENSG00000265190 | ANXA8 | 1.1953403 | 1.45E-10 | 3.25E-09 |
| ENSG00000183049 | CAMK1D | 1.1936647 | 1.92E-10 | 4.22E-09 |
| ENSG00000141574 | SECTM1 | 1.1918516 | 6.08E-07 | 7.32E-06 |
| ENSG00000183779 | ZNF703 | 1.1894284 | 6.86E-18 | 3.64E-16 |
| ENSG00000167772 | ANGPTL4 | 1.1885974 | 9.24E-05 | 0.000665154 |
| ENSG00000198945 | L3MBTL3 | 1.1760215 | 1.51E-05 | 0.000133055 |
| ENSG00000136492 | BRIP1 | 1.1582401 | 5.44E-13 | 1.68E-11 |
| ENSG00000105409 | ATP1A3 | 1.1470539 | 0.000322991 | 0.002019391 |
| ENSG00000160949 | TONSL | 1.1398962 | 1.03E-17 | 5.35E-16 |
| ENSG00000062822 | POLD1 | 1.1333005 | 9.42E-20 | 6.09E-18 |
| ENSG00000065328 | MCM10 | 1.1330254 | 1.56E-21 | 1.25E-19 |
| ENSG00000221963 | APOL6 | 1.131167 | 5.96E-53 | 2.52E-50 |
| ENSG00000155760 | FZD7 | 1.1232756 | 2.12E-32 | 3.26E-30 |
| ENSG00000169258 | GPRIN1 | 1.1211091 | 4.41E-09 | 7.92E-08 |
| ENSG00000042062 | RIPOR3 | 1.1172835 | 2.48E-07 | 3.27E-06 |
| ENSG00000164418 | GRIK2 | 1.1102821 | 0.000438514 | 0.002631081 |
| ENSG00000117228 | GBP1 | 1.108289 | 6.24E-21 | 4.64E-19 |
| ENSG00000135750 | KCNK1 | 1.1025979 | 0.000653428 | 0.003671538 |
| ENSG00000163362 | INAVA | 1.0975041 | 7.70E-12 | 2.03E-10 |
| ENSG00000166508 | MCM7 | 1.0966884 | 2.07E-50 | 7.54E-48 |
| ENSG00000158292 | GPR153 | 1.096508 | 0.000205822 | 0.001362298 |
| ENSG00000154188 | ANGPT1 | 1.0909462 | 0.001594944 | 0.007888558 |
| ENSG00000171051 | FPR1 | 1.0874628 | 0.000712201 | 0.003947559 |
| ENSG00000165474 | GJB2 | 1.0851674 | 0.001128044 | 0.005837296 |
| ENSG00000127423 | AUNIP | 1.0819176 | 0.000559971 | 0.003208374 |
| ENSG00000004799 | PDK4 | 1.0803734 | 0.00690274 | 0.026976319 |
| ENSG00000078900 | TP73 | 1.0792267 | 0.001374549 | 0.006924047 |
| ENSG00000100065 | CARD10 | 1.0770808 | 7.61E-15 | 2.86E-13 |
| ENSG00000105486 | LIG1 | 1.0736441 | 4.74E-17 | 2.32E-15 |
| ENSG00000129675 | ARHGEF6 | 1.0730635 | 0.007561487 | 0.029064881 |
| ENSG00000116525 | TRIM62 | 1.066931 | 7.77E-05 | 0.000571831 |
| ENSG00000138439 | FAM117B | 1.061816 | 9.69E-07 | 1.12E-05 |
| ENSG00000102996 | MMP15 | 1.0615178 | 4.39E-13 | 1.37E-11 |
| ENSG00000113368 | LMNB1 | 1.0475316 | 6.36E-20 | 4.22E-18 |
| ENSG00000087510 | TFAP2C | 1.0451609 | 3.44E-14 | 1.21E-12 |
| ENSG00000128694 | OSGEPL1 | 1.0378682 | 1.99E-07 | 2.69E-06 |
| ENSG00000138944 | SHISAL1 | 1.0352755 | 0.000685725 | 0.003815348 |
| ENSG00000173530 | TNFRSF10D | 1.0351101 | 3.82E-20 | 2.59E-18 |
| ENSG00000161395 | PGAP3 | 1.0332586 | 3.53E-06 | 3.60E-05 |
| ENSG00000169598 | DFFB | 1.0250426 | 4.72E-05 | 0.000368751 |
| ENSG00000131459 | GFPT2 | 1.0199582 | 3.23E-59 | 1.92E-56 |
| ENSG00000171604 | CXXC5 | 1.0192967 | 1.65E-14 | 5.97E-13 |
| ENSG00000160256 | FAM207A | 1.0171898 | 0.002647147 | 0.012147371 |
| ENSG00000101230 | ISM1 | 1.0149503 | 3.61E-10 | 7.61E-09 |
| ENSG00000162337 | LRP5 | 1.0125218 | 2.96E-45 | 8.79E-43 |
| ENSG00000113389 | NPR3 | 1.0107264 | 0.004048905 | 0.017317301 |
| ENSG00000011478 | QPCTL | 1.0101992 | 5.77E-11 | 1.37E-09 |
| ENSG00000114268 | PFKFB4 | 1.0076652 | 9.87E-20 | 6.33E-18 |
| ENSG00000108515 | ENO3 | -1.003458 | 1.66E-15 | 6.92E-14 |
| ENSG00000140941 | MAP1LC3B | -1.003779 | 1.99E-62 | 1.45E-59 |
| ENSG00000058335 | RASGRF1 | -1.004419 | 5.47E-22 | 4.42E-20 |
| ENSG00000164400 | CSF2 | -1.007418 | 4.85E-24 | 4.70E-22 |
| ENSG00000041982 | TNC | -1.0088 | 1.45E-34 | 2.47E-32 |
| ENSG00000186523 | FAM86B1 | -1.012198 | 1.42E-06 | 1.58E-05 |
| ENSG00000136014 | USP44 | -1.0129 | 0.009831103 | 0.036174481 |
| ENSG00000181722 | ZBTB20 | -1.013939 | 4.00E-08 | 6.20E-07 |
| ENSG00000143382 | ADAMTSL4 | -1.020464 | 9.06E-07 | 1.05E-05 |
| ENSG00000099889 | ARVCF | -1.024453 | 0.012447337 | 0.044067208 |
| ENSG00000197261 | C6orf141 | -1.024533 | 0.004539874 | 0.019001288 |
| ENSG00000133134 | BEX2 | -1.027889 | 2.26E-05 | 0.000190165 |
| ENSG00000166192 | SENP8 | -1.028482 | 0.000248203 | 0.001611041 |
| ENSG00000013588 | GPRC5A | -1.034247 | 4.98E-33 | 7.95E-31 |
| ENSG00000179242 | CDH4 | -1.03708 | 8.33E-24 | 7.84E-22 |
| ENSG00000041515 | MYO16 | -1.039221 | 0.000174142 | 0.001166767 |
| ENSG00000129195 | PIMREG | -1.039362 | 0.005917559 | 0.02373583 |
| ENSG00000160207 | HSF2BP | -1.039528 | 0.000975606 | 0.005187908 |
| ENSG00000175556 | LONRF3 | -1.039864 | 4.84E-15 | 1.88E-13 |
| ENSG00000139132 | FGD4 | -1.041223 | 0.00312124 | 0.01397513 |
| ENSG00000065357 | DGKA | -1.045039 | 8.69E-05 | 0.000629375 |
| ENSG00000184792 | OSBP2 | -1.048682 | 1.84E-09 | 3.49E-08 |
| ENSG00000172731 | LRRC20 | -1.049018 | 1.64E-12 | 4.74E-11 |
| ENSG00000124772 | CPNE5 | -1.049188 | 0.000270896 | 0.001733416 |
| ENSG00000163909 | HEYL | -1.050626 | 0.002141754 | 0.010130003 |
| ENSG00000198919 | DZIP3 | -1.064269 | 7.76E-14 | 2.63E-12 |
| ENSG00000137962 | ARHGAP29 | -1.068899 | 6.86E-66 | 5.99E-63 |
| ENSG00000066279 | ASPM | -1.069278 | 2.15E-26 | 2.32E-24 |
| ENSG00000169760 | NLGN1 | -1.072863 | 0.00399839 | 0.01712923 |
| ENSG00000068489 | PRR11 | -1.073036 | 2.39E-20 | 1.67E-18 |
| ENSG00000177943 | MAMDC4 | -1.098032 | 0.000441453 | 0.002646295 |
| ENSG00000240445 | FOXO3B | -1.106828 | 1.71E-06 | 1.87E-05 |
| ENSG00000081181 | ARG2 | -1.119196 | 1.13E-11 | 2.89E-10 |
| ENSG00000078401 | EDN1 | -1.119793 | 1.78E-13 | 5.79E-12 |
| ENSG00000079385 | CEACAM1 | -1.126278 | 4.75E-07 | 5.92E-06 |
| ENSG00000112559 | MDFI | -1.1336 | 0.003519169 | 0.015476304 |
| ENSG00000115008 | IL1A | -1.133821 | 9.77E-54 | 4.41E-51 |
| ENSG00000135114 | OASL | -1.137504 | 0.00152131 | 0.007561497 |
| ENSG00000101096 | NFATC2 | -1.139565 | 3.44E-18 | 1.87E-16 |
| ENSG00000188177 | ZC3H6 | -1.140272 | 1.09E-07 | 1.55E-06 |
| ENSG00000110723 | EXPH5 | -1.140365 | 1.02E-18 | 5.87E-17 |
| ENSG00000156510 | HKDC1 | -1.144989 | 8.73E-41 | 2.00E-38 |
| ENSG00000129757 | CDKN1C | -1.149425 | 0.006888132 | 0.026935313 |
| ENSG00000083457 | ITGAE | -1.156869 | 0.000708247 | 0.003927915 |
| ENSG00000054392 | HHAT | -1.163009 | 3.31E-12 | 9.09E-11 |
| ENSG00000115163 | CENPA | -1.165234 | 0.000469226 | 0.002777172 |
| ENSG00000166851 | PLK1 | -1.165842 | 8.98E-15 | 3.37E-13 |
| ENSG00000122694 | GLIPR2 | -1.167932 | 1.48E-06 | 1.66E-05 |
| ENSG00000183778 | B3GALT5 | -1.173878 | 2.46E-15 | 9.87E-14 |
| ENSG00000138621 | PPCDC | -1.177076 | 1.59E-05 | 0.000139149 |
| ENSG00000164695 | CHMP4C | -1.184267 | 0.000582017 | 0.003317271 |
| ENSG00000183760 | ACP7 | -1.185069 | 0.000504599 | 0.002943944 |
| ENSG00000107864 | CPEB3 | -1.18548 | 9.90E-06 | 9.21E-05 |
| ENSG00000170961 | HAS2 | -1.186096 | 0.00858095 | 0.032412521 |
| ENSG00000100526 | CDKN3 | -1.187121 | 3.10E-09 | 5.75E-08 |
| ENSG00000117013 | KCNQ4 | -1.187627 | 0.006535662 | 0.025813947 |
| ENSG00000014914 | MTMR11 | -1.190345 | 3.52E-14 | 1.23E-12 |
| ENSG00000168811 | IL12A | -1.194362 | 7.90E-07 | 9.27E-06 |
| ENSG00000138356 | AOX1 | -1.19998 | 4.20E-45 | 1.22E-42 |
| ENSG00000111348 | ARHGDIB | -1.212255 | 5.68E-11 | 1.35E-09 |
| ENSG00000080031 | PTPRH | -1.212885 | 4.20E-07 | 5.30E-06 |
| ENSG00000186364 | NUDT17 | -1.213532 | 0.000108913 | 0.000772 |
| ENSG00000170175 | CHRNB1 | -1.214836 | 1.28E-35 | 2.37E-33 |
| ENSG00000131018 | SYNE1 | -1.221686 | 1.19E-19 | 7.59E-18 |
| ENSG00000137821 | LRRC49 | -1.223828 | 2.84E-13 | 9.05E-12 |
| ENSG00000117650 | NEK2 | -1.227138 | 6.14E-08 | 9.22E-07 |
| ENSG00000138772 | ANXA3 | -1.228228 | 1.27E-47 | 4.28E-45 |
| ENSG00000179148 | ALOXE3 | -1.22924 | 4.34E-16 | 1.92E-14 |
| ENSG00000115380 | EFEMP1 | -1.235982 | 4.30E-19 | 2.56E-17 |
| ENSG00000160712 | IL6R | -1.246794 | 2.39E-07 | 3.17E-06 |
| ENSG00000204262 | COL5A2 | -1.252387 | 0.00310703 | 0.013927866 |
| ENSG00000065618 | COL17A1 | -1.252542 | 1.89E-05 | 0.000162174 |
| ENSG00000188015 | S100A3 | -1.255639 | 0.000122269 | 0.000859664 |
| ENSG00000112782 | CLIC5 | -1.256014 | 0.000559603 | 0.003207673 |
| ENSG00000119771 | KLHL29 | -1.262261 | 5.41E-51 | 2.02E-48 |
| ENSG00000189410 | SH2D5 | -1.263034 | 1.51E-10 | 3.36E-09 |
| ENSG00000183696 | UPP1 | -1.270911 | 1.52E-90 | 2.85E-87 |
| ENSG00000221843 | C2orf16 | -1.2824 | 2.86E-05 | 0.000236295 |
| ENSG00000168685 | IL7R | -1.299189 | 6.87E-15 | 2.60E-13 |
| ENSG00000144583 | 4-Mar | -1.301133 | 1.47E-15 | 6.15E-14 |
| ENSG00000135063 | FAM189A2 | -1.304153 | 0.001109938 | 0.005764103 |
| ENSG00000168386 | FILIP1L | -1.30678 | 1.19E-20 | 8.54E-19 |
| ENSG00000134259 | NGF | -1.313301 | 0.000584642 | 0.003328146 |
| ENSG00000048740 | CELF2 | -1.316662 | 2.32E-21 | 1.82E-19 |
| ENSG00000012124 | CD22 | -1.332696 | 9.49E-54 | 4.41E-51 |
| ENSG00000162591 | MEGF6 | -1.334838 | 8.42E-11 | 1.96E-09 |
| ENSG00000183287 | CCBE1 | -1.334984 | 5.40E-05 | 0.00041608 |
| ENSG00000134198 | TSPAN2 | -1.339206 | 0.004919768 | 0.020305677 |
| ENSG00000167895 | TMC8 | -1.346698 | 2.72E-28 | 3.24E-26 |
| ENSG00000135604 | STX11 | -1.347923 | 0.006432568 | 0.025495776 |
| ENSG00000156869 | FRRS1 | -1.361419 | 8.11E-05 | 0.00059226 |
| ENSG00000170396 | ZNF804A | -1.36804 | 3.03E-08 | 4.80E-07 |
| ENSG00000116717 | GADD45A | -1.368914 | 3.33E-75 | 3.35E-72 |
| ENSG00000188910 | GJB3 | -1.369794 | 0.000250946 | 0.001625624 |
| ENSG00000205755 | CRLF2 | -1.371367 | 0.013716423 | 0.047683329 |
| ENSG00000164949 | GEM | -1.402994 | 1.55E-36 | 2.95E-34 |
| ENSG00000167617 | CDC42EP5 | -1.411779 | 0.003246462 | 0.014456695 |
| ENSG00000019186 | CYP24A1 | -1.429988 | 0.011816681 | 0.042153676 |
| ENSG00000164855 | TMEM184A | -1.443786 | 8.84E-13 | 2.65E-11 |
| ENSG00000184545 | DUSP8 | -1.451918 | 5.26E-14 | 1.80E-12 |
| ENSG00000158023 | WDR66 | -1.454888 | 1.10E-20 | 7.90E-19 |
| ENSG00000104177 | MYEF2 | -1.471036 | 1.96E-39 | 4.21E-37 |
| ENSG00000075340 | ADD2 | -1.472275 | 0.000435288 | 0.002615324 |
| ENSG00000139354 | GAS2L3 | -1.478145 | 7.65E-58 | 4.17E-55 |
| ENSG00000186517 | ARHGAP30 | -1.484643 | 6.16E-05 | 0.000464753 |
| ENSG00000137474 | MYO7A | -1.494197 | 9.59E-05 | 0.000688389 |
| ENSG00000138190 | EXOC6 | -1.496268 | 9.96E-07 | 1.14E-05 |
| ENSG00000226742 | HSBP1L1 | -1.505515 | 2.21E-05 | 0.000186601 |
| ENSG00000184185 | KCNJ12 | -1.512813 | 0.002387512 | 0.011131521 |
| ENSG00000078018 | MAP2 | -1.518729 | 0.000144008 | 0.000992813 |
| ENSG00000173212 | MAB21L3 | -1.540568 | 0.014109853 | 0.048817706 |
| ENSG00000144218 | AFF3 | -1.543142 | 5.02E-14 | 1.72E-12 |
| ENSG00000165046 | LETM2 | -1.549464 | 2.10E-17 | 1.07E-15 |
| ENSG00000027869 | SH2D2A | -1.55613 | 0.000195586 | 0.001299801 |
| ENSG00000143375 | CGN | -1.557482 | 0.000117908 | 0.000830365 |
| ENSG00000171877 | FRMD5 | -1.573253 | 3.61E-32 | 5.43E-30 |
| ENSG00000169429 | CXCL8 | -1.583813 | 3.67E-19 | 2.22E-17 |
| ENSG00000166432 | ZMAT1 | -1.606231 | 9.87E-09 | 1.68E-07 |
| ENSG00000135549 | PKIB | -1.611228 | 7.95E-06 | 7.53E-05 |
| ENSG00000127325 | BEST3 | -1.61168 | 0.000115577 | 0.000816145 |
| ENSG00000198673 | TAFA2 | -1.613914 | 1.34E-05 | 0.000120322 |
| ENSG00000149418 | ST14 | -1.619728 | 0.000259893 | 0.001672507 |
| ENSG00000123610 | TNFAIP6 | -1.65355 | 0.00021371 | 0.001412366 |
| ENSG00000145113 | MUC4 | -1.666757 | 4.85E-10 | 9.96E-09 |
| ENSG00000049192 | ADAMTS6 | -1.685566 | 1.45E-45 | 4.42E-43 |
| ENSG00000147168 | IL2RG | -1.689246 | 0.000298325 | 0.001886143 |
| ENSG00000117069 | ST6GALNAC5 | -1.693897 | 4.91E-07 | 6.08E-06 |
| ENSG00000132561 | MATN2 | -1.699963 | 1.66E-09 | 3.16E-08 |
| ENSG00000104413 | ESRP1 | -1.707506 | 1.01E-06 | 1.15E-05 |
| ENSG00000149573 | MPZL2 | -1.726239 | 0.000530128 | 0.003062855 |
| ENSG00000079931 | MOXD1 | -1.744743 | 0.000224818 | 0.00147757 |
| ENSG00000138769 | CDKL2 | -1.756729 | 1.34E-05 | 0.00012018 |
| ENSG00000187800 | PEAR1 | -1.758403 | 2.05E-10 | 4.47E-09 |
| ENSG00000163661 | PTX3 | -1.758788 | 2.71E-105 | 5.92E-102 |
| ENSG00000172602 | RND1 | -1.760617 | 0.000136237 | 0.00094521 |
| ENSG00000284906 | ARHGAP11B | -1.763085 | 0.000166283 | 0.001120999 |
| ENSG00000170381 | SEMA3E | -1.78617 | 0.010770848 | 0.039039851 |
| ENSG00000140030 | GPR65 | -1.788496 | 0.011373582 | 0.040891054 |
| ENSG00000081041 | CXCL2 | -1.791774 | 2.31E-61 | 1.60E-58 |
| ENSG00000133083 | DCLK1 | -1.796128 | 1.92E-08 | 3.12E-07 |
| ENSG00000075213 | SEMA3A | -1.796655 | 2.67E-05 | 0.00022177 |
| ENSG00000116701 | NCF2 | -1.802319 | 2.08E-08 | 3.36E-07 |
| ENSG00000187688 | TRPV2 | -1.81274 | 8.49E-08 | 1.23E-06 |
| ENSG00000182901 | RGS7 | -1.839776 | 5.30E-05 | 0.000408785 |
| ENSG00000197415 | VEPH1 | -1.907702 | 2.73E-11 | 6.66E-10 |
| ENSG00000144063 | MALL | -1.912127 | 0.001179033 | 0.006081917 |
| ENSG00000101333 | PLCB4 | -1.91427 | 0.000452492 | 0.002696462 |
| ENSG00000135253 | KCP | -1.916477 | 2.12E-18 | 1.17E-16 |
| ENSG00000149591 | TAGLN | -1.946099 | 6.45E-15 | 2.46E-13 |
| ENSG00000113578 | FGF1 | -1.986579 | 6.43E-06 | 6.24E-05 |
| ENSG00000176406 | RIMS2 | -2.012881 | 2.41E-15 | 9.73E-14 |
| ENSG00000169085 | VXN | -2.037868 | 1.17E-05 | 0.000106358 |
| ENSG00000243137 | PSG4 | -2.083097 | 1.09E-27 | 1.25E-25 |
| ENSG00000148483 | TMEM236 | -2.10343 | 1.01E-05 | 9.37E-05 |
| ENSG00000163347 | CLDN1 | -2.134531 | 1.07E-87 | 1.56E-84 |
| ENSG00000181634 | TNFSF15 | -2.250418 | 5.25E-158 | 1.72E-154 |
| ENSG00000170498 | KISS1 | -2.276918 | 2.38E-07 | 3.15E-06 |
| ENSG00000170373 | CST1 | -2.296982 | 0.006695968 | 0.026333315 |
| ENSG00000133101 | CCNA1 | -2.30592 | 8.10E-08 | 1.18E-06 |
| ENSG00000111700 | SLCO1B3 | -2.316231 | 1.19E-16 | 5.58E-15 |
| ENSG00000189056 | RELN | -2.376721 | 1.59E-05 | 0.000138777 |
| ENSG00000172967 | XKR3 | -2.400538 | 0.008650606 | 0.03260977 |
| ENSG00000138161 | CUZD1 | -2.434937 | 0.007182429 | 0.027869698 |
| ENSG00000131242 | RAB11FIP4 | -2.436099 | 0.001620823 | 0.00799541 |
| ENSG00000167306 | MYO5B | -2.584963 | 1.63E-06 | 1.80E-05 |
| ENSG00000151702 | FLI1 | -2.588298 | 2.89E-08 | 4.61E-07 |
| ENSG00000165071 | TMEM71 | -2.644625 | 1.66E-31 | 2.42E-29 |
| ENSG00000249751 | ECSCR | -2.690316 | 0.003729184 | 0.016225481 |
| ENSG00000170323 | FABP4 | -2.713696 | 0.002313527 | 0.010821254 |
| ENSG00000198074 | AKR1B10 | -2.743902 | 0.008163037 | 0.031030917 |
| ENSG00000163121 | NEURL3 | -2.974615 | 6.15E-09 | 1.08E-07 |
| ENSG00000047457 | CP | -3.017074 | 8.85E-06 | 8.31E-05 |
| ENSG00000169994 | MYO7B | -3.087463 | 2.29E-09 | 4.28E-08 |
| ENSG00000196188 | CTSE | -3.566347 | 0.000228003 | 0.001496248 |
| ENSG00000093134 | VNN3 | -3.616259 | 1.12E-11 | 2.88E-10 |
| ENSG00000100867 | DHRS2 | -4.146841 | 3.00E-07 | 3.90E-06 |
| ENSG00000151967 | SCHIP1 | -4.203872 | 2.44E-05 | 0.000204143 |
| ENSG00000205670 | SMIM11A | -4.781804 | 0.006819644 | 0.026715373 |
| ENSG00000215182 | MUC5AC | -5.066089 | 1.39E-08 | 2.31E-07 |
| ENSG00000117983 | MUC5B | -7.544321 | 0.012712263 | 0.044772889 |
| ENSG00000086548 | CEACAM6 | -7.697663 | 1.03E-09 | 2.03E-08 |

**Supplemental Table 5. DEGs in control vs Olaparib treated group analyzed by RNA sequencing.**

| ID | Symbol | log_2_(fc) | P Value | FDR |
| --- | --- | --- | --- | --- |
| ENSG00000162892 | IL24 | -1.298333 | 5.05E-85 | 6.34E-81 |
| ENSG00000119922 | IFIT2 | 1.5845192 | 1.66E-66 | 5.21E-63 |
| ENSG00000185745 | IFIT1 | 1.9751315 | 6.18E-36 | 5.54E-33 |
| ENSG00000173530 | TNFRSF10D | 1.2759206 | 3.89E-30 | 1.88E-27 |
| ENSG00000164283 | ESM1 | 1.9144222 | 1.24E-27 | 5.18E-25 |
| ENSG00000184992 | BRI3BP | 1.2776296 | 1.85E-27 | 7.25E-25 |
| ENSG00000175305 | CCNE2 | 1.6797298 | 8.56E-26 | 2.99E-23 |
| ENSG00000092853 | CLSPN | 1.2256041 | 3.02E-20 | 6.54E-18 |
| ENSG00000183778 | B3GALT5 | -1.275323 | 8.47E-16 | 9.41E-14 |
| ENSG00000066056 | TIE1 | 1.0698281 | 3.03E-15 | 3.07E-13 |
| ENSG00000187608 | ISG15 | 1.1596783 | 4.21E-14 | 3.62E-12 |
| ENSG00000189057 | FAM111B | 1.7411534 | 1.03E-13 | 8.42E-12 |
| ENSG00000133119 | RFC3 | 1.0182742 | 2.07E-12 | 1.29E-10 |
| ENSG00000258465 | AL139011.2 | 1.5275043 | 6.28E-11 | 2.89E-09 |
| ENSG00000132561 | MATN2 | 1.1688658 | 1.72E-10 | 7.06E-09 |
| ENSG00000286132 | AC022415.2 | 1.0218831 | 2.15E-10 | 8.72E-09 |
| ENSG00000171320 | ESCO2 | 1.6523707 | 2.29E-10 | 9.19E-09 |
| ENSG00000135114 | OASL | 1.4112492 | 2.74E-10 | 1.08E-08 |
| ENSG00000137965 | IFI44 | 1.1036356 | 3.47E-10 | 1.32E-08 |
| ENSG00000130487 | KLHDC7B | -1.699295 | 6.76E-10 | 2.45E-08 |
| ENSG00000120738 | EGR1 | -1.054079 | 8.54E-09 | 2.43E-07 |
| ENSG00000241322 | CDRT1 | -1.208674 | 2.04E-08 | 5.30E-07 |
| ENSG00000136982 | DSCC1 | 1.1331251 | 1.02E-07 | 2.19E-06 |
| ENSG00000137628 | DDX60 | 1.042178 | 1.52E-07 | 3.06E-06 |
| ENSG00000267041 | ZNF850 | 1.140725 | 2.78E-07 | 5.36E-06 |
| ENSG00000204287 | HLA-DRA | -1.03827 | 3.76E-07 | 6.93E-06 |
| ENSG00000124788 | ATXN1 | -1.246316 | 5.11E-07 | 9.08E-06 |
| ENSG00000150281 | CTF1 | -1.06805 | 6.53E-07 | 1.13E-05 |
| ENSG00000093134 | VNN3 | -2.039758 | 1.07E-06 | 1.76E-05 |
| ENSG00000205923 | CEMP1 | 6.3750394 | 1.17E-06 | 1.89E-05 |
| ENSG00000162894 | FCMR | -1.364498 | 1.65E-06 | 2.60E-05 |
| ENSG00000147614 | ATP6V0D2 | 1.1119386 | 2.38E-06 | 3.58E-05 |
| ENSG00000198056 | PRIM1 | 1.1787743 | 3.62E-06 | 5.14E-05 |
| ENSG00000152147 | GEMIN6 | 1.1572456 | 4.18E-06 | 5.78E-05 |
| ENSG00000181544 | FANCB | 1.2142069 | 4.52E-06 | 6.18E-05 |
| ENSG00000011052 | NME1-NME2 | 1.3136841 | 4.79E-06 | 6.47E-05 |
| ENSG00000154175 | ABI3BP | 1.073114 | 7.93E-06 | 0.0001 |
| ENSG00000251537 | AC005324.3 | -1.169925 | 1.79E-05 | 0.000201 |
| ENSG00000145569 | OTULINL | 1.0280144 | 2.68E-05 | 0.000285 |
| ENSG00000105290 | APLP1 | 1.255915 | 4.14E-05 | 0.000416 |
| ENSG00000284969 | AL049629.2 | 1.9514915 | 4.56E-05 | 0.000452 |
| ENSG00000081853 | PCDHGA2 | -1.532733 | 5.18E-05 | 0.000507 |
| ENSG00000095739 | BAMBI | 1.0035145 | 0.0001086 | 0.000969 |
| ENSG00000186642 | PDE2A | -1.050878 | 0.0001383 | 0.001188 |
| ENSG00000064489 | BORCS8-MEF2B | -1.174586 | 0.0001826 | 0.001496 |
| ENSG00000111319 | SCNN1A | -1.079387 | 0.0001907 | 0.001553 |
| ENSG00000171931 | FBXW10 | -1.005901 | 0.0002231 | 0.001774 |
| ENSG00000162654 | GBP4 | 1.0665977 | 0.0002664 | 0.002055 |
| ENSG00000164692 | COL1A2 | -7.67948 | 0.0002841 | 0.002169 |
| ENSG00000188015 | S100A3 | -1.045676 | 0.0003348 | 0.002485 |
| ENSG00000171443 | ZNF524 | -1.15624 | 0.0003899 | 0.002826 |
| ENSG00000085840 | ORC1 | 1.0780025 | 0.0004764 | 0.003317 |
| ENSG00000007968 | E2F2 | 1.5643522 | 0.0007143 | 0.004639 |
| ENSG00000068615 | REEP1 | 1.91908 | 0.0007626 | 0.004884 |
| ENSG00000123570 | RAB9B | 1.0551416 | 0.0011716 | 0.006993 |
| ENSG00000168542 | COL3A1 | -4.675565 | 0.0012884 | 0.007568 |
| ENSG00000258677 | AC022826.2 | -1.281487 | 0.0014094 | 0.008191 |
| ENSG00000183128 | CALHM3 | -1.5807 | 0.0017821 | 0.009869 |
| ENSG00000115738 | ID2 | 1.7064803 | 0.0018898 | 0.010292 |
| ENSG00000170962 | PDGFD | 1.09586 | 0.0024116 | 0.012578 |
| ENSG00000180616 | SSTR2 | 1.2129937 | 0.0025367 | 0.013127 |
| ENSG00000215182 | MUC5AC | -4.481127 | 0.0030507 | 0.01521 |
| ENSG00000186517 | ARHGAP30 | -1.144184 | 0.0032159 | 0.015863 |
| ENSG00000284906 | ARHGAP11B | -1.206137 | 0.0033047 | 0.016181 |
| ENSG00000129757 | CDKN1C | -1.115477 | 0.0041379 | 0.019559 |
| ENSG00000136237 | RAPGEF5 | 1.8259706 | 0.0041846 | 0.019737 |
| ENSG00000166396 | SERPINB7 | 1.2137793 | 0.0044012 | 0.020564 |
| ENSG00000170442 | KRT86 | -1.239188 | 0.0044729 | 0.020775 |
| ENSG00000147676 | MAL2 | 1.9891932 | 0.0045488 | 0.021026 |
| ENSG00000086548 | CEACAM6 | -8.434628 | 0.0046454 | 0.021379 |
| ENSG00000196456 | ZNF775 | -1.106915 | 0.0046735 | 0.021484 |
| ENSG00000064687 | ABCA7 | 1.032193 | 0.0047275 | 0.021661 |
| ENSG00000135750 | KCNK1 | 1.002871 | 0.0057001 | 0.025074 |
| ENSG00000169851 | PCDH7 | 1.1255309 | 0.0057489 | 0.025245 |
| ENSG00000235109 | ZSCAN31 | -1.106073 | 0.0058758 | 0.02573 |
| ENSG00000242221 | PSG2 | 1.1214314 | 0.0066251 | 0.028432 |
| ENSG00000179841 | AKAP5 | 1.6374299 | 0.0076955 | 0.031956 |
| ENSG00000079393 | DUSP13 | 1.284096 | 0.0077862 | 0.032232 |
| ENSG00000174721 | FGFBP3 | -1.060696 | 0.0089751 | 0.035932 |
| ENSG00000197506 | SLC28A3 | 1.16711 | 0.0096557 | 0.038079 |
| ENSG00000243708 | PLA2G4B | -1.040957 | 0.0096756 | 0.03814 |
| ENSG00000080493 | SLC4A4 | 1.3785116 | 0.010528 | 0.040646 |
| ENSG00000105388 | CEACAM5 | -6.558421 | 0.0107092 | 0.041257 |
| ENSG00000169684 | CHRNA5 | 1.2090513 | 0.0115277 | 0.043779 |
| ENSG00000269313 | MAGIX | -1.517848 | 0.0117164 | 0.044321 |
| ENSG00000170469 | SPATA24 | -1.231243 | 0.0122534 | 0.046023 |
| ENSG00000038427 | VCAN | -1.179324 | 0.0130112 | 0.048317 |
| ENSG00000170122 | FOXD4 | -1.36257 | 0.0132473 | 0.049049 |
| ENSG00000214967 | NPIPA7 | -1.165115 | 0.0133851 | 0.049442 |
| ENSG00000275074 | NUDT18 | -1.300233 | 0.0135446 | 0.049782 |

**Supplemental Table 6. DEGs in control vs FDI-6/Olaparib co-treated group analyzed by RNA sequencing.**

| ID | Symbol | log_2_(fc) | P Value | FDR |
| --- | --- | --- | --- | --- |
| ENSG00000138061 | CYP1B1 | 1.964662 | 0 | 0 |
| ENSG00000149968 | MMP3 | 2.338077 | 0 | 0 |
| ENSG00000196611 | MMP1 | 3.670781 | 5.06E-264 | 2.33E-260 |
| ENSG00000109971 | HSPA8 | 1.320681 | 8.04E-257 | 2.77E-253 |
| ENSG00000197632 | SERPINB2 | 3.716255 | 1.21E-256 | 3.34E-253 |
| ENSG00000163661 | PTX3 | -1.75586 | 9.97E-216 | 2.29E-212 |
| ENSG00000213949 | ITGA1 | 1.659922 | 1.78E-187 | 3.52E-184 |
| ENSG00000140465 | CYP1A1 | 3.032828 | 8.66E-176 | 1.49E-172 |
| ENSG00000106366 | SERPINE1 | 1.003481 | 9.98E-172 | 1.53E-168 |
| ENSG00000163659 | TIPARP | 1.398939 | 1.04E-146 | 1.43E-143 |
| ENSG00000125538 | IL1B | 1.151807 | 3.01E-135 | 3.78E-132 |
| ENSG00000041982 | TNC | -1.97081 | 1.33E-133 | 1.53E-130 |
| ENSG00000157227 | MMP14 | 1.15329 | 3.72E-114 | 3.66E-111 |
| ENSG00000137801 | THBS1 | 1.236189 | 1.53E-108 | 1.41E-105 |
| ENSG00000130513 | GDF15 | -1.12092 | 1.36E-104 | 1.17E-101 |
| ENSG00000104738 | MCM4 | 1.29192 | 2.82E-100 | 2.29E-97 |
| ENSG00000101255 | TRIB3 | -1.17954 | 2.95E-92 | 2.27E-89 |
| ENSG00000172432 | GTPBP2 | -1.07394 | 6.61E-89 | 4.80E-86 |
| ENSG00000184254 | ALDH1A3 | 1.771466 | 1.05E-87 | 7.25E-85 |
| ENSG00000163347 | CLDN1 | -1.79765 | 2.36E-82 | 1.55E-79 |
| ENSG00000171848 | RRM2 | 1.595653 | 1.28E-73 | 7.66E-71 |
| ENSG00000111799 | COL12A1 | -1.0041 | 3.27E-71 | 1.88E-68 |
| ENSG00000112118 | MCM3 | 1.141438 | 1.73E-68 | 9.57E-66 |
| ENSG00000163638 | ADAMTS9 | 1.259622 | 1.20E-67 | 6.39E-65 |
| ENSG00000189060 | H1F0 | -1.00196 | 4.70E-66 | 2.24E-63 |
| ENSG00000108846 | ABCC3 | -1.32749 | 1.49E-61 | 6.41E-59 |
| ENSG00000131016 | AKAP12 | 1.805476 | 2.09E-61 | 8.72E-59 |
| ENSG00000163131 | CTSS | 1.005583 | 3.90E-57 | 1.54E-54 |
| ENSG00000089723 | OTUB2 | 1.471463 | 1.73E-56 | 6.43E-54 |
| ENSG00000072310 | SREBF1 | -1.01407 | 3.60E-52 | 1.18E-49 |
| ENSG00000105825 | TFPI2 | 1.046346 | 4.14E-51 | 1.29E-48 |
| ENSG00000167767 | KRT80 | -1.05798 | 5.03E-51 | 1.51E-48 |
| ENSG00000119771 | KLHL29 | -1.13237 | 2.35E-50 | 6.90E-48 |
| ENSG00000092853 | CLSPN | 1.687047 | 2.64E-48 | 7.01E-46 |
| ENSG00000144810 | COL8A1 | -1.11599 | 3.96E-48 | 1.03E-45 |
| ENSG00000132510 | KDM6B | -1.33982 | 5.98E-48 | 1.53E-45 |
| ENSG00000134369 | NAV1 | -1.04311 | 4.22E-44 | 9.54E-42 |
| ENSG00000128283 | CDC42EP1 | -1.11332 | 8.67E-44 | 1.90E-41 |
| ENSG00000132646 | PCNA | 1.175768 | 1.01E-43 | 2.18E-41 |
| ENSG00000143476 | DTL | 1.318002 | 1.90E-43 | 4.03E-41 |
| ENSG00000108239 | TBC1D12 | 1.108362 | 5.43E-43 | 1.12E-40 |
| ENSG00000175305 | CCNE2 | 2.152761 | 7.50E-43 | 1.52E-40 |
| ENSG00000114812 | VIPR1 | 1.582077 | 9.64E-43 | 1.93E-40 |
| ENSG00000276043 | UHRF1 | 1.163744 | 2.08E-42 | 4.11E-40 |
| ENSG00000139354 | GAS2L3 | -1.37513 | 2.57E-42 | 5.00E-40 |
| ENSG00000076003 | MCM6 | 1.078602 | 1.14E-40 | 2.10E-38 |
| ENSG00000180730 | SHISA2 | 1.851685 | 5.37E-39 | 9.15E-37 |
| ENSG00000173530 | TNFRSF10D | 1.397823 | 3.50E-38 | 5.74E-36 |
| ENSG00000121858 | TNFSF10 | 2.079126 | 5.88E-38 | 9.55E-36 |
| ENSG00000119714 | GPR68 | 1.372566 | 5.84E-37 | 8.96E-35 |
| ENSG00000180573 | HIST1H2AC | 1.209051 | 7.17E-37 | 1.08E-34 |
| ENSG00000058335 | RASGRF1 | -1.24397 | 1.05E-36 | 1.54E-34 |
| ENSG00000179242 | CDH4 | -1.25345 | 2.54E-36 | 3.62E-34 |
| ENSG00000095002 | MSH2 | 1.004318 | 8.96E-36 | 1.21E-33 |
| ENSG00000164949 | GEM | -1.18662 | 5.25E-34 | 6.41E-32 |
| ENSG00000119922 | IFIT2 | 1.231953 | 8.61E-34 | 1.03E-31 |
| ENSG00000179532 | DNHD1 | -1.06277 | 1.39E-33 | 1.66E-31 |
| ENSG00000184992 | BRI3BP | 1.368616 | 1.33E-32 | 1.52E-30 |
| ENSG00000094804 | CDC6 | 1.027286 | 4.43E-32 | 4.93E-30 |
| ENSG00000011201 | ANOS1 | 1.184139 | 7.18E-32 | 7.93E-30 |
| ENSG00000164283 | ESM1 | 1.982758 | 7.62E-32 | 8.35E-30 |
| ENSG00000110031 | LPXN | 1.408567 | 5.31E-31 | 5.64E-29 |
| ENSG00000167797 | CDK2AP2 | -1.02806 | 1.16E-30 | 1.21E-28 |
| ENSG00000147614 | ATP6V0D2 | 2.287208 | 1.18E-30 | 1.21E-28 |
| ENSG00000065328 | MCM10 | 1.2997 | 2.10E-29 | 1.96E-27 |
| ENSG00000189057 | FAM111B | 2.204912 | 2.88E-29 | 2.63E-27 |
| ENSG00000179046 | TRIML2 | -1.31509 | 5.43E-29 | 4.86E-27 |
| ENSG00000101412 | E2F1 | 1.312768 | 1.30E-27 | 1.12E-25 |
| ENSG00000164251 | F2RL1 | 1.386322 | 1.40E-27 | 1.20E-25 |
| ENSG00000165071 | TMEM71 | -2.10715 | 1.64E-27 | 1.40E-25 |
| ENSG00000146678 | IGFBP1 | 1.195682 | 1.73E-26 | 1.35E-24 |
| ENSG00000171992 | SYNPO | -1.04066 | 6.16E-26 | 4.78E-24 |
| ENSG00000132846 | ZBED3 | -1.08104 | 8.76E-26 | 6.64E-24 |
| ENSG00000169129 | AFAP1L2 | 1.379414 | 1.39E-25 | 1.05E-23 |
| ENSG00000197299 | BLM | 1.269914 | 3.62E-25 | 2.67E-23 |
| ENSG00000167895 | TMC8 | -1.11524 | 1.21E-24 | 8.63E-23 |
| ENSG00000154310 | TNIK | 1.298209 | 1.84E-24 | 1.30E-22 |
| ENSG00000106948 | AKNA | -1.13781 | 2.76E-24 | 1.89E-22 |
| ENSG00000172086 | KRCC1 | -1.02549 | 3.34E-24 | 2.27E-22 |
| ENSG00000132613 | MTSS2 | -1.00122 | 3.44E-24 | 2.33E-22 |
| ENSG00000185745 | IFIT1 | 1.574594 | 1.50E-23 | 9.53E-22 |
| ENSG00000102312 | PORCN | 1.093421 | 1.02E-22 | 6.24E-21 |
| ENSG00000180884 | ZNF792 | 1.658471 | 1.68E-21 | 9.63E-20 |
| ENSG00000182752 | PAPPA | 1.01719 | 2.13E-21 | 1.20E-19 |
| ENSG00000197635 | DPP4 | 1.548825 | 3.20E-21 | 1.79E-19 |
| ENSG00000133119 | RFC3 | 1.227457 | 6.97E-21 | 3.80E-19 |
| ENSG00000130487 | KLHDC7B | -3.07039 | 7.06E-20 | 3.46E-18 |
| ENSG00000164045 | CDC25A | 1.065479 | 2.16E-19 | 1.02E-17 |
| ENSG00000198056 | PRIM1 | 1.895252 | 3.59E-19 | 1.68E-17 |
| ENSG00000171877 | FRMD5 | -1.30031 | 5.34E-19 | 2.47E-17 |
| ENSG00000112297 | CRYBG1 | -1.22038 | 2.12E-18 | 9.27E-17 |
| ENSG00000187608 | ISG15 | 1.256634 | 3.19E-18 | 1.38E-16 |
| ENSG00000136982 | DSCC1 | 1.659163 | 7.61E-18 | 3.18E-16 |
| ENSG00000108515 | ENO3 | -1.13327 | 1.22E-17 | 5.04E-16 |
| ENSG00000158373 | HIST1H2BD | 1.319359 | 2.42E-17 | 9.60E-16 |
| ENSG00000168386 | FILIP1L | -1.10284 | 2.47E-17 | 9.76E-16 |
| ENSG00000177602 | HASPIN | 1.232204 | 2.48E-17 | 9.80E-16 |
| ENSG00000100297 | MCM5 | 1.011799 | 2.52E-17 | 9.90E-16 |
| ENSG00000165244 | ZNF367 | 1.06815 | 6.76E-17 | 2.58E-15 |
| ENSG00000092470 | WDR76 | 1.612539 | 8.44E-17 | 3.19E-15 |
| ENSG00000178764 | ZHX2 | -1.05637 | 1.13E-16 | 4.20E-15 |
| ENSG00000159147 | DONSON | 1.012215 | 1.49E-16 | 5.50E-15 |
| ENSG00000267041 | ZNF850 | 1.44575 | 1.62E-16 | 5.99E-15 |
| ENSG00000135253 | KCP | -1.82562 | 3.84E-16 | 1.36E-14 |
| ENSG00000144681 | STAC | -1.23887 | 6.11E-16 | 2.12E-14 |
| ENSG00000274559 | CU639417.1 | 1.303839 | 7.98E-16 | 2.73E-14 |
| ENSG00000154734 | ADAMTS1 | 1.180236 | 8.80E-16 | 2.98E-14 |
| ENSG00000165046 | LETM2 | -1.56984 | 1.34E-15 | 4.45E-14 |
| ENSG00000258465 | AL139011.2 | 1.818124 | 1.46E-15 | 4.83E-14 |
| ENSG00000265190 | ANXA8 | 1.419803 | 1.47E-15 | 4.84E-14 |
| ENSG00000144354 | CDCA7 | 1.183646 | 1.51E-15 | 4.95E-14 |
| ENSG00000108932 | SLC16A6 | 1.876397 | 3.28E-15 | 1.04E-13 |
| ENSG00000136492 | BRIP1 | 1.177787 | 3.62E-15 | 1.15E-13 |
| ENSG00000221869 | CEBPD | -1.10751 | 7.64E-15 | 2.33E-13 |
| ENSG00000099998 | GGT5 | -1.70083 | 1.44E-14 | 4.28E-13 |
| ENSG00000196584 | XRCC2 | 1.130483 | 2.62E-14 | 7.43E-13 |
| ENSG00000174371 | EXO1 | 1.096845 | 6.48E-14 | 1.75E-12 |
| ENSG00000259207 | ITGB3 | 1.100973 | 8.23E-14 | 2.19E-12 |
| ENSG00000169258 | GPRIN1 | 1.371598 | 1.00E-13 | 2.64E-12 |
| ENSG00000049769 | PPP1R3F | -1.13548 | 1.00E-13 | 2.64E-12 |
| ENSG00000286132 | AC022415.2 | 1.145215 | 1.19E-13 | 3.07E-12 |
| ENSG00000241322 | CDRT1 | -1.39289 | 1.60E-13 | 4.08E-12 |
| ENSG00000167772 | ANGPTL4 | 2.036837 | 1.97E-13 | 4.94E-12 |
| ENSG00000164855 | TMEM184A | -1.54507 | 2.19E-13 | 5.44E-12 |
| ENSG00000171320 | ESCO2 | 1.640661 | 2.73E-13 | 6.76E-12 |
| ENSG00000183778 | B3GALT5 | -1.33382 | 4.41E-13 | 1.06E-11 |
| ENSG00000158483 | FAM86C1 | -1.04824 | 5.99E-13 | 1.43E-11 |
| ENSG00000102996 | MMP15 | 1.014002 | 1.24E-12 | 2.85E-11 |
| ENSG00000181722 | ZBTB20 | -1.21662 | 1.49E-12 | 3.38E-11 |
| ENSG00000131018 | SYNE1 | -1.1123 | 1.75E-12 | 3.93E-11 |
| ENSG00000100479 | POLE2 | 1.36062 | 7.11E-12 | 1.48E-10 |
| ENSG00000137752 | CASP1 | 1.172213 | 8.50E-12 | 1.74E-10 |
| ENSG00000164379 | FOXQ1 | 1.944949 | 1.08E-11 | 2.18E-10 |
| ENSG00000184792 | OSBP2 | -1.13305 | 1.14E-11 | 2.28E-10 |
| ENSG00000137821 | LRRC49 | -1.01514 | 1.46E-11 | 2.88E-10 |
| ENSG00000104413 | ESRP1 | -2.11235 | 1.63E-11 | 3.20E-10 |
| ENSG00000144554 | FANCD2 | 1.027013 | 1.93E-11 | 3.79E-10 |
| ENSG00000101901 | ALG13 | -1.27124 | 2.17E-11 | 4.23E-10 |
| ENSG00000242419 | PCDHGC4 | -2.98453 | 3.03E-11 | 5.78E-10 |
| ENSG00000078081 | LAMP3 | -1.28021 | 5.25E-11 | 9.72E-10 |
| ENSG00000081181 | ARG2 | -1.07262 | 5.36E-11 | 9.92E-10 |
| ENSG00000204406 | MBD5 | -1.15321 | 5.52E-11 | 1.02E-09 |
| ENSG00000101003 | GINS1 | 1.207452 | 5.75E-11 | 1.06E-09 |
| ENSG00000186523 | FAM86B1 | -1.25308 | 7.72E-11 | 1.39E-09 |
| ENSG00000138346 | DNA2 | 1.13236 | 1.17E-10 | 2.06E-09 |
| ENSG00000132326 | PER2 | -1.18386 | 1.58E-10 | 2.71E-09 |
| ENSG00000166801 | FAM111A | 1.049374 | 1.67E-10 | 2.86E-09 |
| ENSG00000166396 | SERPINB7 | 2.210897 | 2.29E-10 | 3.83E-09 |
| ENSG00000184545 | DUSP8 | -1.11631 | 2.78E-10 | 4.57E-09 |
| ENSG00000157110 | RBPMS | 1.013772 | 3.72E-10 | 6.04E-09 |
| ENSG00000184271 | POU6F1 | -1.8005 | 4.31E-10 | 6.92E-09 |
| ENSG00000186871 | ERCC6L | 1.146357 | 7.27E-10 | 1.12E-08 |
| ENSG00000143603 | KCNN3 | 1.224966 | 1.02E-09 | 1.53E-08 |
| ENSG00000167513 | CDT1 | 1.043873 | 1.28E-09 | 1.90E-08 |
| ENSG00000167191 | GPRC5B | 1.159819 | 1.43E-09 | 2.11E-08 |
| ENSG00000168936 | TMEM129 | -1.05802 | 1.55E-09 | 2.27E-08 |
| ENSG00000132470 | ITGB4 | -1.12037 | 2.30E-09 | 3.29E-08 |
| ENSG00000187688 | TRPV2 | -1.97232 | 2.74E-09 | 3.85E-08 |
| ENSG00000166922 | SCG5 | 1.571526 | 2.99E-09 | 4.18E-08 |
| ENSG00000180596 | HIST1H2BC | 1.529992 | 3.88E-09 | 5.32E-08 |
| ENSG00000175287 | PHYHD1 | -1.12127 | 4.16E-09 | 5.69E-08 |
| ENSG00000170054 | SERPINA9 | 5.095924 | 4.76E-09 | 6.48E-08 |
| ENSG00000124788 | ATXN1 | -1.41695 | 6.10E-09 | 8.19E-08 |
| ENSG00000181544 | FANCB | 1.499977 | 7.99E-09 | 1.06E-07 |
| ENSG00000103888 | CEMIP | 2.470027 | 9.68E-09 | 1.26E-07 |
| ENSG00000131153 | GINS2 | 1.43958 | 1.35E-08 | 1.72E-07 |
| ENSG00000105290 | APLP1 | 1.736966 | 1.36E-08 | 1.73E-07 |
| ENSG00000182378 | PLCXD1 | 1.504706 | 1.60E-08 | 1.99E-07 |
| ENSG00000170915 | PAQR8 | -1.7632 | 1.62E-08 | 2.02E-07 |
| ENSG00000085840 | ORC1 | 1.557995 | 2.07E-08 | 2.54E-07 |
| ENSG00000188177 | ZC3H6 | -1.05161 | 2.40E-08 | 2.90E-07 |
| ENSG00000138395 | CDK15 | 2.825971 | 2.48E-08 | 3.00E-07 |
| ENSG00000114491 | UMPS | 1.223948 | 3.58E-08 | 4.20E-07 |
| ENSG00000093134 | VNN3 | -2.16375 | 4.16E-08 | 4.81E-07 |
| ENSG00000116675 | DNAJC6 | -1.01455 | 5.03E-08 | 5.69E-07 |
| ENSG00000129173 | E2F8 | 1.195551 | 5.62E-08 | 6.30E-07 |
| ENSG00000188015 | S100A3 | -1.93597 | 6.82E-08 | 7.54E-07 |
| ENSG00000147804 | SLC39A4 | -1.08875 | 8.69E-08 | 9.43E-07 |
| ENSG00000051180 | RAD51 | 1.012318 | 8.70E-08 | 9.43E-07 |
| ENSG00000123096 | SSPN | -2.08549 | 9.57E-08 | 1.03E-06 |
| ENSG00000197415 | VEPH1 | -1.10296 | 1.28E-07 | 1.34E-06 |
| ENSG00000167889 | MGAT5B | -1.04803 | 1.57E-07 | 1.62E-06 |
| ENSG00000128510 | CPA4 | 1.009438 | 1.83E-07 | 1.87E-06 |
| ENSG00000112149 | CD83 | 1.062429 | 2.01E-07 | 2.03E-06 |
| ENSG00000076770 | MBNL3 | -1.03148 | 2.78E-07 | 2.75E-06 |
| ENSG00000283782 | AC116366.3 | -2.1078 | 2.78E-07 | 2.75E-06 |
| ENSG00000150281 | CTF1 | -1.06173 | 3.12E-07 | 3.06E-06 |
| ENSG00000177943 | MAMDC4 | -1.60618 | 3.25E-07 | 3.19E-06 |
| ENSG00000132010 | ZNF20 | 1.102234 | 3.30E-07 | 3.22E-06 |
| ENSG00000169085 | VXN | -2.57009 | 3.48E-07 | 3.38E-06 |
| ENSG00000176406 | RIMS2 | -1.25536 | 3.54E-07 | 3.43E-06 |
| ENSG00000175899 | A2M | -6.90087 | 3.62E-07 | 3.50E-06 |
| ENSG00000127423 | AUNIP | 1.396791 | 4.97E-07 | 4.68E-06 |
| ENSG00000206073 | SERPINB4 | 1.019687 | 5.27E-07 | 4.93E-06 |
| ENSG00000132394 | EEFSEC | -1.01544 | 5.54E-07 | 5.16E-06 |
| ENSG00000115392 | FANCL | 1.065879 | 5.76E-07 | 5.35E-06 |
| ENSG00000189410 | SH2D5 | -1.21195 | 6.28E-07 | 5.80E-06 |
| ENSG00000241360 | PDXP | 1.019256 | 9.81E-07 | 8.77E-06 |
| ENSG00000078018 | MAP2 | -1.45098 | 9.97E-07 | 8.90E-06 |
| ENSG00000102882 | MAPK3 | -1.04079 | 1.26E-06 | 1.10E-05 |
| ENSG00000157379 | DHRS1 | 1.151144 | 1.29E-06 | 1.12E-05 |
| ENSG00000251537 | AC005324.3 | -1.4892 | 1.34E-06 | 1.16E-05 |
| ENSG00000166432 | ZMAT1 | -1.3757 | 1.67E-06 | 1.43E-05 |
| ENSG00000254122 | PCDHGB7 | -1.23178 | 1.74E-06 | 1.48E-05 |
| ENSG00000164220 | F2RL2 | -1.29177 | 1.88E-06 | 1.60E-05 |
| ENSG00000169760 | NLGN1 | -1.71753 | 1.95E-06 | 1.66E-05 |
| ENSG00000100867 | DHRS2 | -3.07973 | 1.96E-06 | 1.66E-05 |
| ENSG00000153292 | ADGRF1 | 3.008989 | 2.13E-06 | 1.79E-05 |
| ENSG00000187800 | PEAR1 | -1.13088 | 2.21E-06 | 1.86E-05 |
| ENSG00000163121 | NEURL3 | -2.088 | 2.25E-06 | 1.89E-05 |
| ENSG00000077942 | FBLN1 | 1.130041 | 2.28E-06 | 1.91E-05 |
| ENSG00000139269 | INHBE | -1.12832 | 2.88E-06 | 2.36E-05 |
| ENSG00000105270 | CLIP3 | -1.14202 | 3.61E-06 | 2.90E-05 |
| ENSG00000142765 | SYTL1 | -1.73448 | 3.62E-06 | 2.90E-05 |
| ENSG00000141574 | SECTM1 | 1.408146 | 4.33E-06 | 3.42E-05 |
| ENSG00000185339 | TCN2 | -1.04424 | 4.69E-06 | 3.66E-05 |
| ENSG00000162490 | DRAXIN | 1.37707 | 4.69E-06 | 3.67E-05 |
| ENSG00000075340 | ADD2 | -1.25835 | 7.64E-06 | 5.72E-05 |
| ENSG00000135114 | OASL | 1.097517 | 9.23E-06 | 6.80E-05 |
| ENSG00000140379 | BCL2A1 | 2.168542 | 9.38E-06 | 6.90E-05 |
| ENSG00000160781 | PAQR6 | -1.39417 | 9.78E-06 | 7.17E-05 |
| ENSG00000158292 | GPR153 | 1.275832 | 1.13E-05 | 8.23E-05 |
| ENSG00000198945 | L3MBTL3 | 1.096215 | 1.17E-05 | 8.44E-05 |
| ENSG00000115525 | ST3GAL5 | 1.124906 | 1.32E-05 | 9.42E-05 |
| ENSG00000160256 | FAM207A | 1.412874 | 1.35E-05 | 9.62E-05 |
| ENSG00000111319 | SCNN1A | -1.34453 | 1.47E-05 | 0.000104197 |
| ENSG00000075240 | GRAMD4 | -1.01973 | 1.55E-05 | 0.000109037 |
| ENSG00000171951 | SCG2 | -1.15973 | 1.58E-05 | 0.000110966 |
| ENSG00000095739 | BAMBI | 1.041619 | 1.75E-05 | 0.00012162 |
| ENSG00000166997 | CNPY4 | 1.329946 | 2.17E-05 | 0.000148534 |
| ENSG00000280893 | AC009133.6 | 1.953661 | 2.51E-05 | 0.00016916 |
| ENSG00000154920 | EME1 | 1.437228 | 2.51E-05 | 0.000169221 |
| ENSG00000169994 | MYO7B | -1.80453 | 2.61E-05 | 0.000174707 |
| ENSG00000149403 | GRIK4 | -1.39689 | 2.68E-05 | 0.000179347 |
| ENSG00000147536 | GINS4 | 1.10212 | 2.72E-05 | 0.000181759 |
| ENSG00000126785 | RHOJ | -1.00576 | 2.89E-05 | 0.000191564 |
| ENSG00000072163 | LIMS2 | -1.32843 | 3.05E-05 | 0.000200924 |
| ENSG00000064687 | ABCA7 | 1.170306 | 3.14E-05 | 0.000206327 |
| ENSG00000075213 | SEMA3A | -1.13894 | 3.79E-05 | 0.000244612 |
| ENSG00000164619 | BMPER | 1.284815 | 4.56E-05 | 0.000288939 |
| ENSG00000144063 | MALL | -2.23406 | 4.61E-05 | 0.000291328 |
| ENSG00000081853 | PCDHGA2 | -1.49476 | 5.51E-05 | 0.000342063 |
| ENSG00000241697 | TMEFF1 | 1.588629 | 6.54E-05 | 0.000397795 |
| ENSG00000167306 | MYO5B | -1.93963 | 6.57E-05 | 0.000399169 |
| ENSG00000153294 | ADGRF4 | 4.209453 | 8.86E-05 | 0.00052269 |
| ENSG00000176401 | EID2B | 1.213639 | 8.89E-05 | 0.000523986 |
| ENSG00000171115 | GIMAP8 | 7.291554 | 9.07E-05 | 0.000532956 |
| ENSG00000215182 | MUC5AC | -7.80305 | 9.41E-05 | 0.000550593 |
| ENSG00000177294 | FBXO39 | -2.22239 | 9.42E-05 | 0.000550887 |
| ENSG00000091513 | TF | -1.81831 | 9.68E-05 | 0.000565059 |
| ENSG00000197191 | CYSRT1 | 1.788901 | 9.69E-05 | 0.000565229 |
| ENSG00000133083 | DCLK1 | -1.00646 | 0.000112282 | 0.000647508 |
| ENSG00000100311 | PDGFB | 1.237039 | 0.000121991 | 0.000696803 |
| ENSG00000139971 | ARMH4 | 1.127281 | 0.000122144 | 0.000697388 |
| ENSG00000162654 | GBP4 | 1.098778 | 0.00012428 | 0.000707286 |
| ENSG00000179715 | PCED1B | -1.63716 | 0.000125119 | 0.000711429 |
| ENSG00000138376 | BARD1 | 1.043381 | 0.000126818 | 0.000719903 |
| ENSG00000185499 | MUC1 | -1.61262 | 0.000129033 | 0.000730075 |
| ENSG00000171931 | FBXW10 | -1.12286 | 0.000130363 | 0.000736997 |
| ENSG00000113739 | STC2 | -1.16146 | 0.000137467 | 0.000772727 |
| ENSG00000272196 | HIST2H2AA4 | 1.224587 | 0.000208327 | 0.00112126 |
| ENSG00000212724 | KRTAP2-3 | 2.511714 | 0.000263495 | 0.001381546 |
| ENSG00000118785 | SPP1 | -1.68101 | 0.000275504 | 0.001440683 |
| ENSG00000135678 | CPM | 1.398772 | 0.000294036 | 0.001523777 |
| ENSG00000187134 | AKR1C1 | -1.62372 | 0.000318806 | 0.001637945 |
| ENSG00000205923 | CEMP1 | 5.145677 | 0.000358642 | 0.001817578 |
| ENSG00000162062 | TEDC2 | 1.848447 | 0.000478343 | 0.002352504 |
| ENSG00000130475 | FCHO1 | -1.49158 | 0.000482452 | 0.002367651 |
| ENSG00000132207 | SLX1A | 4.406964 | 0.000518452 | 0.002522791 |
| ENSG00000226742 | HSBP1L1 | -1.14591 | 0.000530275 | 0.002574875 |
| ENSG00000273331 | TM4SF19-TCTEX1D2 | 1.226696 | 0.000545222 | 0.002640022 |
| ENSG00000110203 | FOLR3 | 4.274481 | 0.000548661 | 0.002655742 |
| ENSG00000213398 | LCAT | -1.28036 | 0.000558897 | 0.002699605 |
| ENSG00000100302 | RASD2 | 1.256062 | 0.000574259 | 0.002759321 |
| ENSG00000188064 | WNT7B | 1.106915 | 0.000583317 | 0.002800894 |
| ENSG00000158406 | HIST1H4H | 1.053467 | 0.000614258 | 0.002933135 |
| ENSG00000286190 | AC055839.2 | -1.17759 | 0.000669381 | 0.00316243 |
| ENSG00000168237 | GLYCTK | -1.30947 | 0.000684304 | 0.003216413 |
| ENSG00000255071 | SAA2-SAA4 | 1.676684 | 0.000707759 | 0.003315369 |
| ENSG00000007968 | E2F2 | 1.532874 | 0.000744842 | 0.003467888 |
| ENSG00000250722 | SELENOP | -2.65662 | 0.000774104 | 0.003577572 |
| ENSG00000175643 | RMI2 | 1.096002 | 0.000805858 | 0.003706947 |
| ENSG00000215126 | CBWD6 | -1.01788 | 0.000838508 | 0.003836674 |
| ENSG00000196826 | AC008758.1 | 6.643856 | 0.000867912 | 0.003948957 |
| ENSG00000182162 | P2RY8 | 2.547488 | 0.000891752 | 0.004044853 |
| ENSG00000163431 | LMOD1 | -1.7028 | 0.000891919 | 0.004044853 |
| ENSG00000138622 | HCN4 | 1.956931 | 0.000910285 | 0.004120019 |
| ENSG00000101115 | SALL4 | 1.683816 | 0.000935013 | 0.004219485 |
| ENSG00000186364 | NUDT17 | -1.00297 | 0.000936572 | 0.004223929 |
| ENSG00000267740 | AC024592.3 | -1.025 | 0.000945872 | 0.004257511 |
| ENSG00000286905 | AC108488.2 | -1.07912 | 0.000993427 | 0.004449625 |
| ENSG00000165474 | GJB2 | 1.091281 | 0.001079699 | 0.004789409 |
| ENSG00000134259 | NGF | -1.20778 | 0.001127183 | 0.004971278 |
| ENSG00000165197 | VEGFD | -2.71484 | 0.00118592 | 0.00519711 |
| ENSG00000260238 | PMF1-BGLAP | 1.064012 | 0.001210724 | 0.00529404 |
| ENSG00000080298 | RFX3 | -1.07966 | 0.001212514 | 0.005297643 |
| ENSG00000171219 | CDC42BPG | -1.78427 | 0.001236377 | 0.005385735 |
| ENSG00000182901 | RGS7 | -1.42884 | 0.001249987 | 0.005433013 |
| ENSG00000115226 | FNDC4 | 1.437267 | 0.001341915 | 0.005785202 |
| ENSG00000136960 | ENPP2 | 1.666492 | 0.001377269 | 0.005917227 |
| ENSG00000135750 | KCNK1 | 1.06463 | 0.00143789 | 0.006133811 |
| ENSG00000115844 | DLX2 | -1.28063 | 0.001463688 | 0.006230379 |
| ENSG00000112559 | MDFI | -1.28504 | 0.001555856 | 0.006580085 |
| ENSG00000196188 | CTSE | -2.45943 | 0.001595283 | 0.006722107 |
| ENSG00000110944 | IL23A | 1.833058 | 0.001702904 | 0.007111152 |
| ENSG00000253485 | PCDHGA5 | -1.23133 | 0.001703069 | 0.007111152 |
| ENSG00000038427 | VCAN | -2.17932 | 0.001759729 | 0.007310131 |
| ENSG00000000971 | CFH | -1.33842 | 0.001865671 | 0.007706154 |
| ENSG00000118513 | MYB | 1.954196 | 0.001870438 | 0.007721224 |
| ENSG00000275493 | AL627230.1 | -10.2167 | 0.002007569 | 0.008191736 |
| ENSG00000167272 | POP5 | 1.049162 | 0.00219862 | 0.008861262 |
| ENSG00000173702 | MUC13 | -5 | 0.002215175 | 0.008912365 |
| ENSG00000197253 | TPSB2 | -1.73697 | 0.002265556 | 0.009091212 |
| ENSG00000268975 | MIA-RAB4B | 1.930098 | 0.002278316 | 0.009139755 |
| ENSG00000133106 | EPSTI1 | -1.25904 | 0.00232061 | 0.009279748 |
| ENSG00000253846 | PCDHGA10 | -1.2076 | 0.002340625 | 0.009348949 |
| ENSG00000128422 | KRT17 | -3.64 | 0.002341453 | 0.009349391 |
| ENSG00000249624 | AP000295.1 | 1.156276 | 0.002388264 | 0.009503473 |
| ENSG00000228253 | MT-ATP8 | -1.65156 | 0.00255174 | 0.010038202 |
| ENSG00000180998 | GPR137C | 1.062464 | 0.002613233 | 0.010245061 |
| ENSG00000129757 | CDKN1C | -1.13235 | 0.002718341 | 0.010584962 |
| ENSG00000286088 | AC073585.2 | 7.321928 | 0.003043243 | 0.011672251 |
| ENSG00000214814 | FER1L6 | 2.807355 | 0.003044515 | 0.011673882 |
| ENSG00000243414 | TICAM2 | 3.044394 | 0.003206276 | 0.012192509 |
| ENSG00000128408 | RIBC2 | 1.924813 | 0.003366804 | 0.012708397 |
| ENSG00000149573 | MPZL2 | -1.35184 | 0.00344509 | 0.012969563 |
| ENSG00000168306 | ACOX2 | -1.5221 | 0.003452432 | 0.012992125 |
| ENSG00000177989 | ODF3B | -2.34031 | 0.003563006 | 0.01335039 |
| ENSG00000133101 | CCNA1 | -1.05956 | 0.00363474 | 0.013589654 |
| ENSG00000153404 | PLEKHG4B | 2.273018 | 0.003654554 | 0.013648943 |
| ENSG00000183128 | CALHM3 | -1.49399 | 0.003661081 | 0.013665922 |
| ENSG00000176020 | AMIGO3 | -3.39689 | 0.003889378 | 0.014391097 |
| ENSG00000285991 | AL355312.6 | 2.253757 | 0.003889768 | 0.014391097 |
| ENSG00000180616 | SSTR2 | 1.140481 | 0.004296032 | 0.015646673 |
| ENSG00000179627 | ZBTB42 | 1.258016 | 0.004557678 | 0.016447686 |
| ENSG00000183647 | ZNF530 | 1.270891 | 0.004575848 | 0.01650894 |
| ENSG00000267059 | AC005943.1 | -1.30068 | 0.0047111 | 0.016912898 |
| ENSG00000180509 | KCNE1 | -2.35252 | 0.004826707 | 0.017278484 |
| ENSG00000187824 | TMEM220 | -1.27417 | 0.005047716 | 0.01791167 |
| ENSG00000275778 | AC018630.2 | -1.00753 | 0.00505007 | 0.017915417 |
| ENSG00000168032 | ENTPD3 | 1.548893 | 0.00518528 | 0.018300989 |
| ENSG00000109472 | CPE | -1.37638 | 0.005206897 | 0.018358503 |
| ENSG00000170381 | SEMA3E | -1.13409 | 0.005235266 | 0.018444388 |
| ENSG00000057657 | PRDM1 | 1.220048 | 0.005358428 | 0.018806284 |
| ENSG00000175322 | ZNF519 | 1.394279 | 0.005602351 | 0.019523345 |
| ENSG00000161939 | RNASEK-C17orf49 | 1.223331 | 0.00573629 | 0.019924691 |
| ENSG00000204262 | COL5A2 | -1.1304 | 0.005782283 | 0.020064616 |
| ENSG00000138190 | EXOC6 | -1.18552 | 0.005803105 | 0.02012132 |
| ENSG00000275074 | NUDT18 | -1.50834 | 0.005949361 | 0.02053703 |
| ENSG00000179331 | RAB39A | 1.664133 | 0.006490818 | 0.022062386 |
| ENSG00000186469 | GNG2 | -1.40078 | 0.006551917 | 0.022242672 |
| ENSG00000135925 | WNT10A | -1.09289 | 0.006646379 | 0.022532345 |
| ENSG00000132837 | DMGDH | -1.63227 | 0.006727173 | 0.022753673 |
| ENSG00000138769 | CDKL2 | -1.09115 | 0.006754944 | 0.022830818 |
| ENSG00000285920 | AC087721.2 | -3.24793 | 0.006816533 | 0.023016433 |
| ENSG00000118137 | APOA1 | -4.30117 | 0.006977255 | 0.023467248 |
| ENSG00000131650 | KREMEN2 | 1.982722 | 0.0071977 | 0.024121726 |
| ENSG00000149418 | ST14 | -1.08246 | 0.00735008 | 0.024571489 |
| ENSG00000257446 | ZNF878 | 1.771376 | 0.007834577 | 0.025939818 |
| ENSG00000154493 | C10orf90 | 1.584963 | 0.008000858 | 0.026376456 |
| ENSG00000112837 | TBX18 | -3.24793 | 0.008050213 | 0.02649053 |
| ENSG00000197978 | GOLGA6L9 | -1.10813 | 0.008120091 | 0.026672235 |
| ENSG00000108771 | DHX58 | -1.31893 | 0.008274735 | 0.027136727 |
| ENSG00000165092 | ALDH1A1 | -6.71081 | 0.008355382 | 0.027355698 |
| ENSG00000198074 | AKR1B10 | -3.2363 | 0.008398402 | 0.027476992 |
| ENSG00000197568 | HHLA3 | -1.29078 | 0.008721821 | 0.028380364 |
| ENSG00000120328 | PCDHB12 | -1.11847 | 0.008829189 | 0.028682394 |
| ENSG00000284906 | ARHGAP11B | -1.0972 | 0.008990602 | 0.029122155 |
| ENSG00000122432 | SPATA1 | -2.51694 | 0.009216845 | 0.029717865 |
| ENSG00000167550 | RHEBL1 | 1.657475 | 0.00938438 | 0.030152374 |
| ENSG00000152580 | IGSF10 | 1.784271 | 0.009730082 | 0.031047529 |
| ENSG00000168955 | TM4SF20 | -4.32193 | 0.010130392 | 0.032152577 |
| ENSG00000270757 | HSPE1-MOB4 | 4.075949 | 0.010580901 | 0.033344698 |
| ENSG00000163689 | C3orf67 | -1.0091 | 0.010891872 | 0.034176405 |
| ENSG00000143919 | CAMKMT | -1.42369 | 0.010955495 | 0.034329207 |
| ENSG00000228570 | NUTM2E | 3.115477 | 0.011198397 | 0.034922345 |
| ENSG00000117425 | PTCH2 | -1.12199 | 0.011224046 | 0.03497219 |
| ENSG00000170442 | KRT86 | -1.21573 | 0.0116819 | 0.036105311 |
| ENSG00000180316 | PNPLA1 | -1.41871 | 0.011815811 | 0.036453877 |
| ENSG00000157890 | MEGF11 | -2.69561 | 0.011851684 | 0.036548211 |
| ENSG00000106560 | GIMAP2 | 4.017922 | 0.012779238 | 0.038955927 |
| ENSG00000117983 | MUC5B | -5.80735 | 0.012792329 | 0.038987221 |
| ENSG00000179841 | AKAP5 | 1.300395 | 0.013170668 | 0.039972556 |
| ENSG00000168273 | SMIM4 | 1.299489 | 0.013646535 | 0.041100433 |
| ENSG00000187595 | ZNF385C | -1.76275 | 0.013811003 | 0.041505191 |
| ENSG00000131095 | GFAP | -2.36457 | 0.013899966 | 0.04172711 |
| ENSG00000172476 | RAB40A | -1.35199 | 0.014265989 | 0.042603472 |
| ENSG00000105538 | RASIP1 | -1.82716 | 0.014583298 | 0.043326053 |
| ENSG00000133048 | CHI3L1 | -3.14296 | 0.014678291 | 0.043552013 |
| ENSG00000155792 | DEPTOR | 1.208317 | 0.015008035 | 0.044339731 |
| ENSG00000196730 | DAPK1 | -3.04439 | 0.01521923 | 0.044896403 |
| ENSG00000284969 | AL049629.2 | 1.415619 | 0.015415525 | 0.045368789 |
| ENSG00000080493 | SLC4A4 | 1.286881 | 0.015471101 | 0.045503238 |
| ENSG00000086548 | CEACAM6 | -11.7566 | 0.015747127 | 0.046206753 |
| ENSG00000088386 | SLC15A1 | 1.791413 | 0.016034742 | 0.046950868 |
| ENSG00000103154 | NECAB2 | -1.8009 | 0.016283272 | 0.047517266 |
| ENSG00000068615 | REEP1 | 1.013056 | 0.016294219 | 0.047539159 |
| ENSG00000185955 | C7orf61 | 2.30117 | 0.016356501 | 0.047670477 |
| ENSG00000004776 | HSPB6 | -2.72935 | 0.016512371 | 0.048053716 |
| ENSG00000109511 | ANXA10 | 1.682428 | 0.016643944 | 0.048375406 |
| ENSG00000142675 | CNKSR1 | -1.00978 | 0.017031364 | 0.049387018 |

1. **Supplemental Figures**

**Supplemental Fig. 1**

**
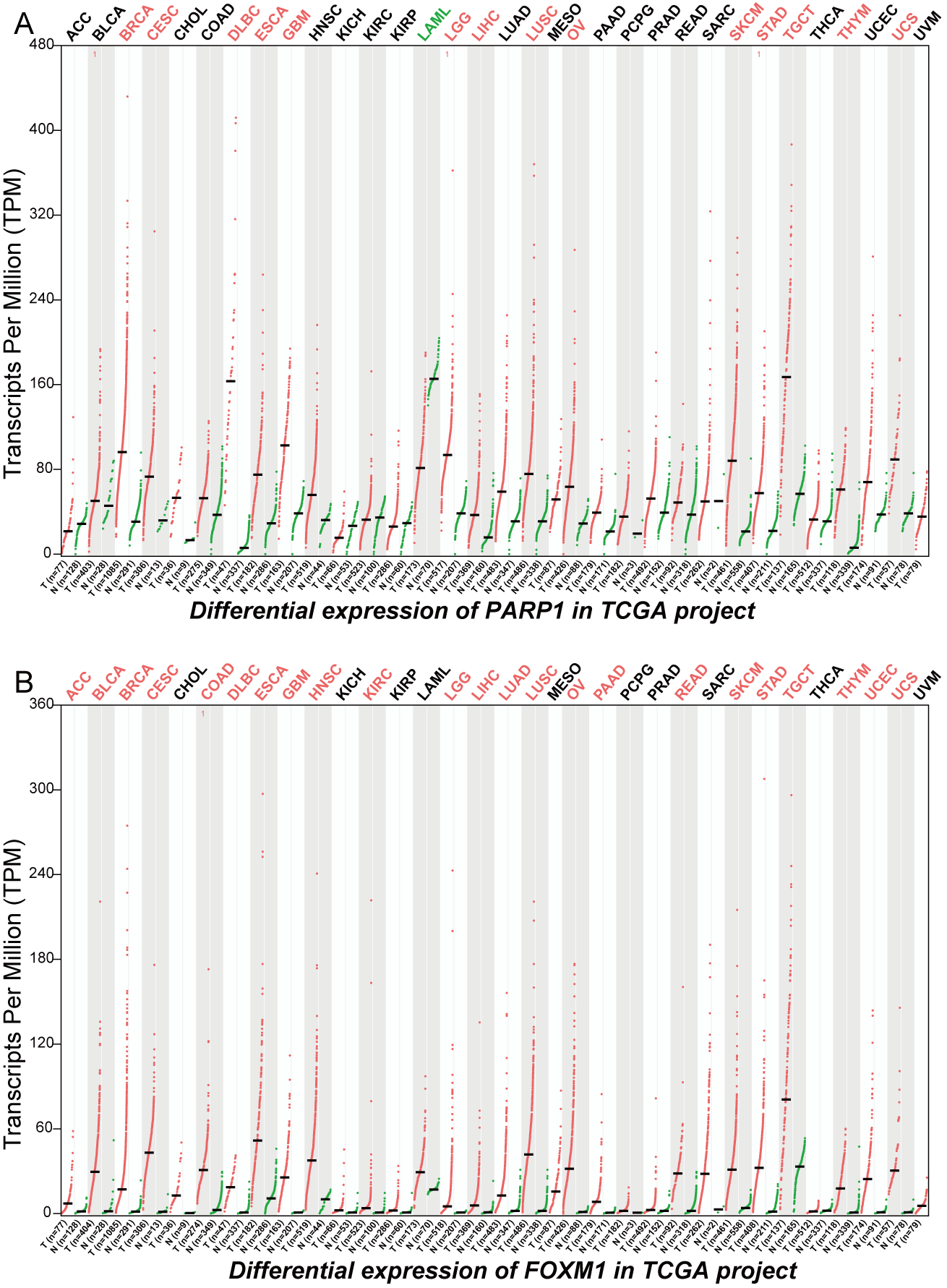
**

**Supplemental Fig. 1. Differential expression of PARP1 and FOXM1 in the TCGA project.** Differential expression of PARP1 (A) and FOXM1 (B) between tumor tissues and adjacent tissues in the TCGA project. Red represents the expression of genes in tumor tissues is higher than in adjacent normal tissues, Green represents the expression of genes in tumor tissues is lower than in adjacent normal tissues.

**Supplemental Fig. 2**

**
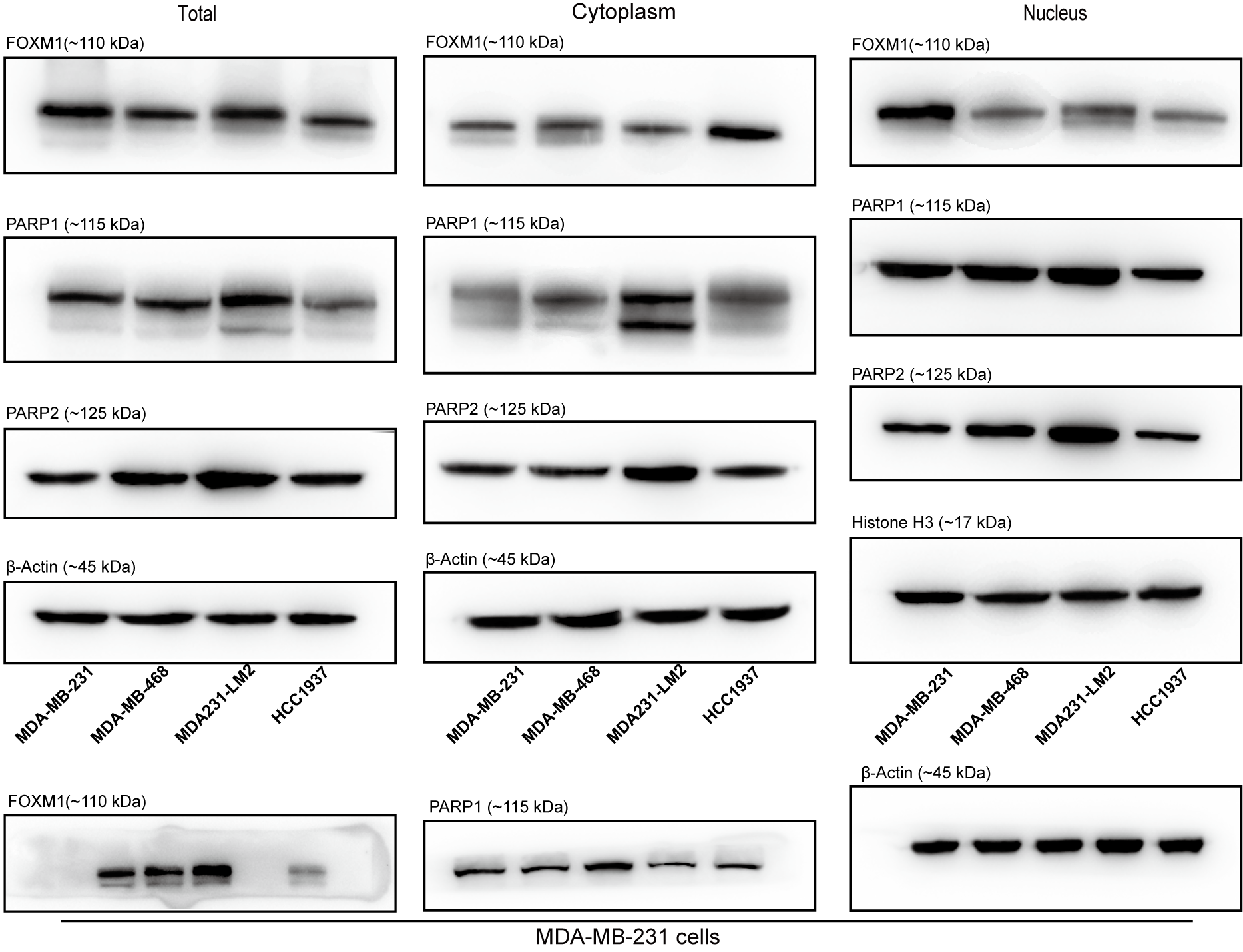
Supplemental Fig. 2. Full length images of FOXM1, PARP1, PARP2 Histone H3 and β-Actin in four TNBC cell lines MDA-MB-231, MDA-MB-468, MDA231-LM2 and HCC1937.** The dilution ratios of all primary antibodies are 1:1000.

**Supplemental Fig. 3**

**
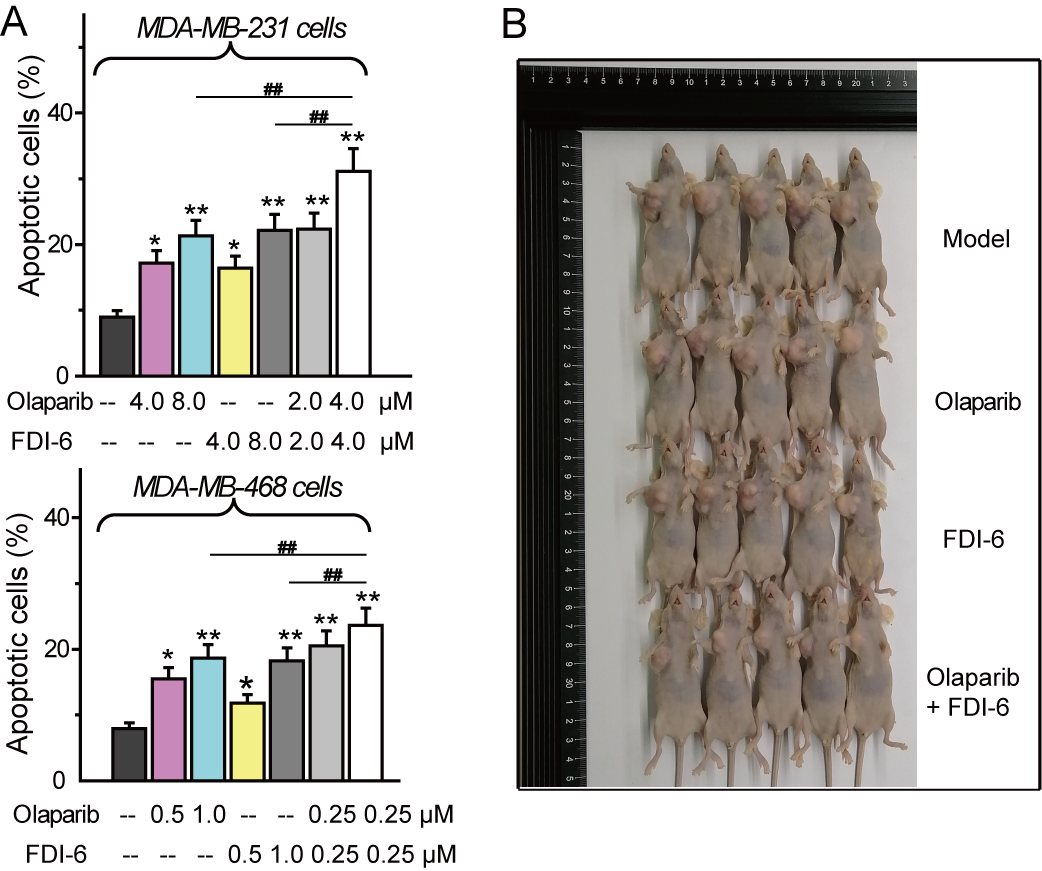
**

**Supplemental Fig. 3. FDI-6 and Olaparib synergistically inhibit the growth of MDA-MB-231 cells *in vitro* and *in vivo*.** (A). Percentage of apoptotic cells in MDA-MB-231 cells and MDA-MB-468 cells. (B). The mice model for the effects of FDI-6 and/or Olaparib on the growth of MDA-MB-231 tumor xenografts. The results from three independent experiments were statistically analyzed using one-way ANOVA: ^*^P<0.05, ^**^P<0.01 compared with the control; ^#^P<0.05, ^##^P<0.01 compared with the FDI-6/Olaparib (4.0 + 4.0 μM or 0.25 + 0.25 μM) combined group.

**Supplemental Fig. 4**

**
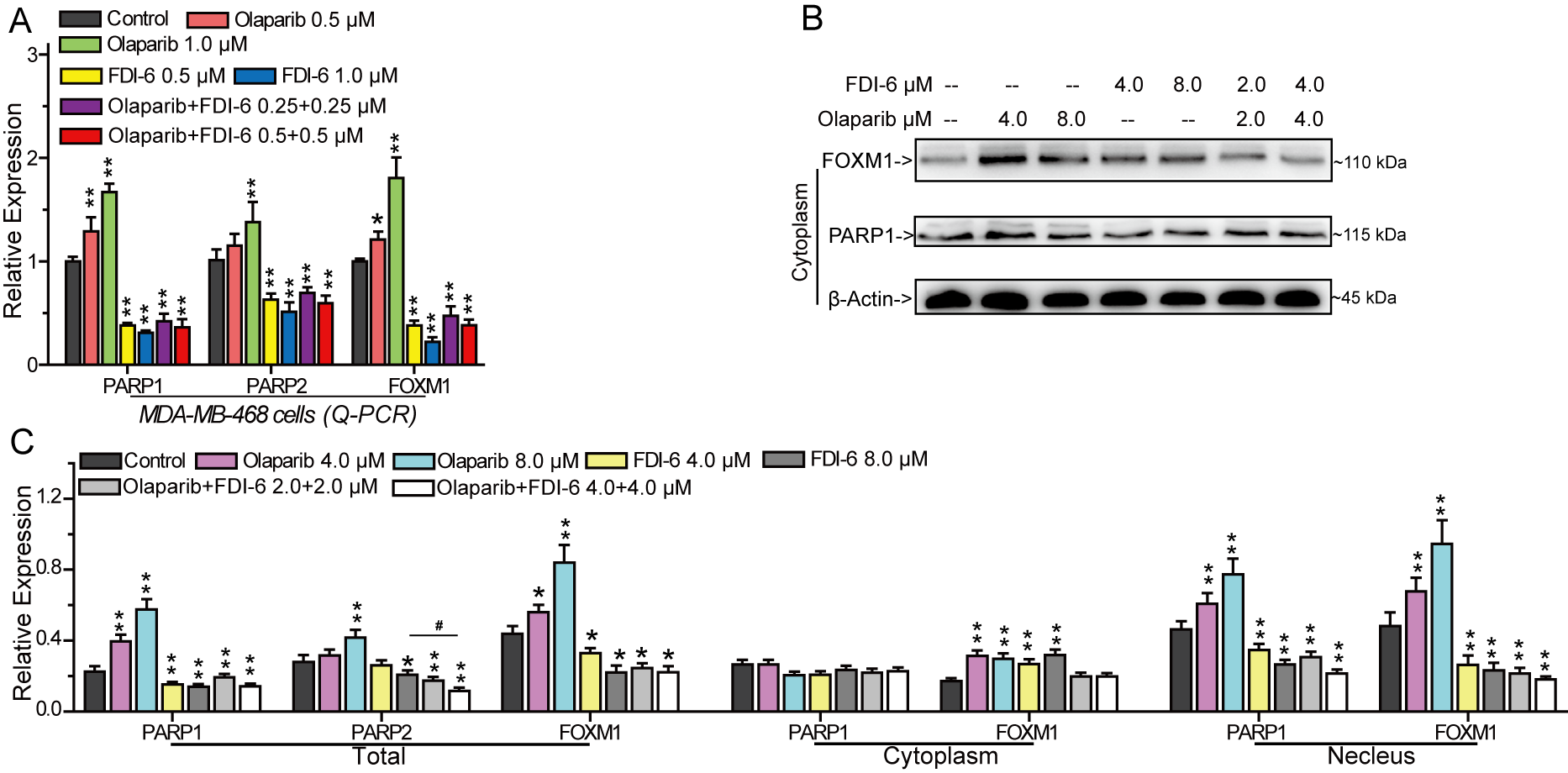
Supplemental Fig. 4. FDI-6 and Olaparib synergistically inhibit the expression of FOXM1, PARP1 and PARP2 *in vitro* and *in vivo*.** (A). The expression of PARP1, PARP2 and FOXM1 *in vitro* analyzed by Q-PCR. (B). The expression of PARP1, PARP2 and FOXM1 in Cytoplasm analyzed by Western bolts. (C). The expression of PARP1, PARP2 and FOXM1 *in vitro* analyzed by Western bolts. The results from three independent experiments were statistically analyzed using one-way ANOVA: ^*^P<0.05, ^**^P<0.01 compared with the control; ^#^P<0.05, ^##^P<0.01 compared with the FDI-6/Olaparib (4.0 + 4.0 μM or 0.5 + 0.5 μM) combined group.

**Supplemental Fig. 5**


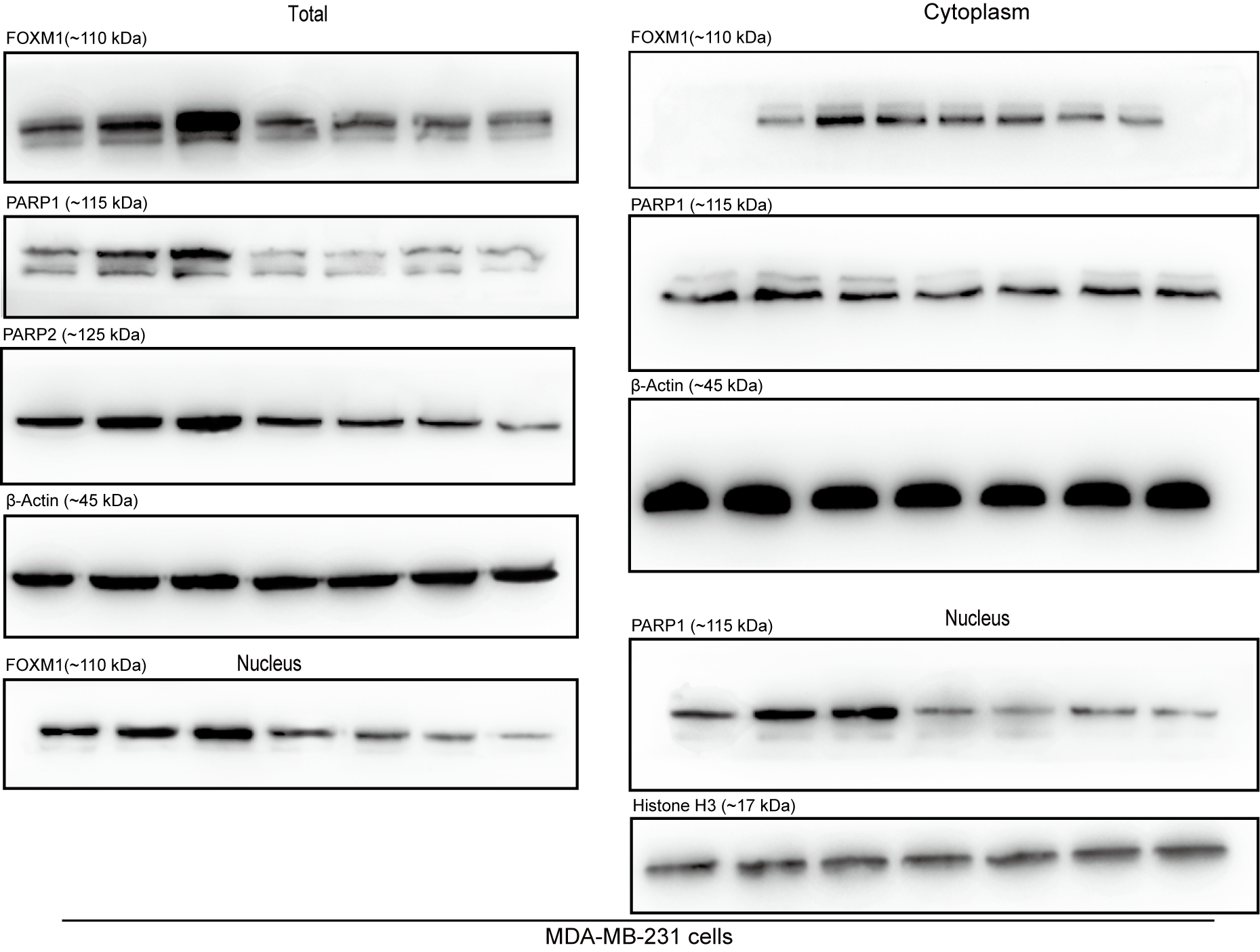
**Supplemental Fig. 5. Full length images of FOXM1, PARP1, PARP2 Histone H3 and β-Actin in MDA-MB-231 cells treated with FDI-6, Olaparib or their combination.** The dilution ratios of all primary antibodies are 1:1000.

**Supplemental Fig. 6**


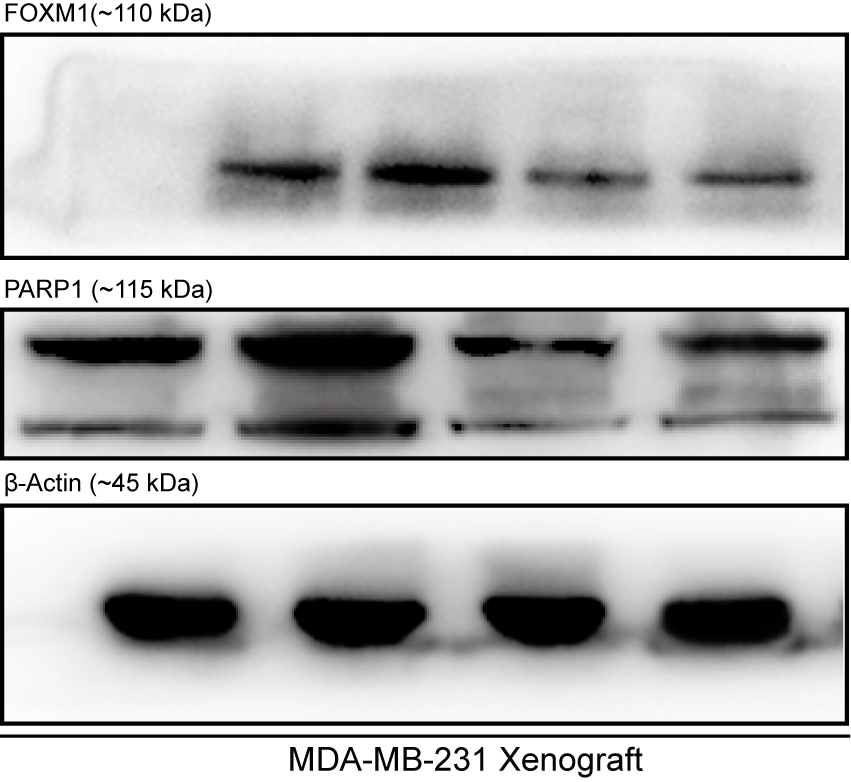


**Supplemental Fig. 6. Full length images of FOXM1, PARP1 and β-Actin in MDA-MB-231 xenograft treated with FDI-6, Olaparib or their combination.** The dilution ratios of all primary antibodies are 1:1000.

**Supplemental Fig. 7**

**
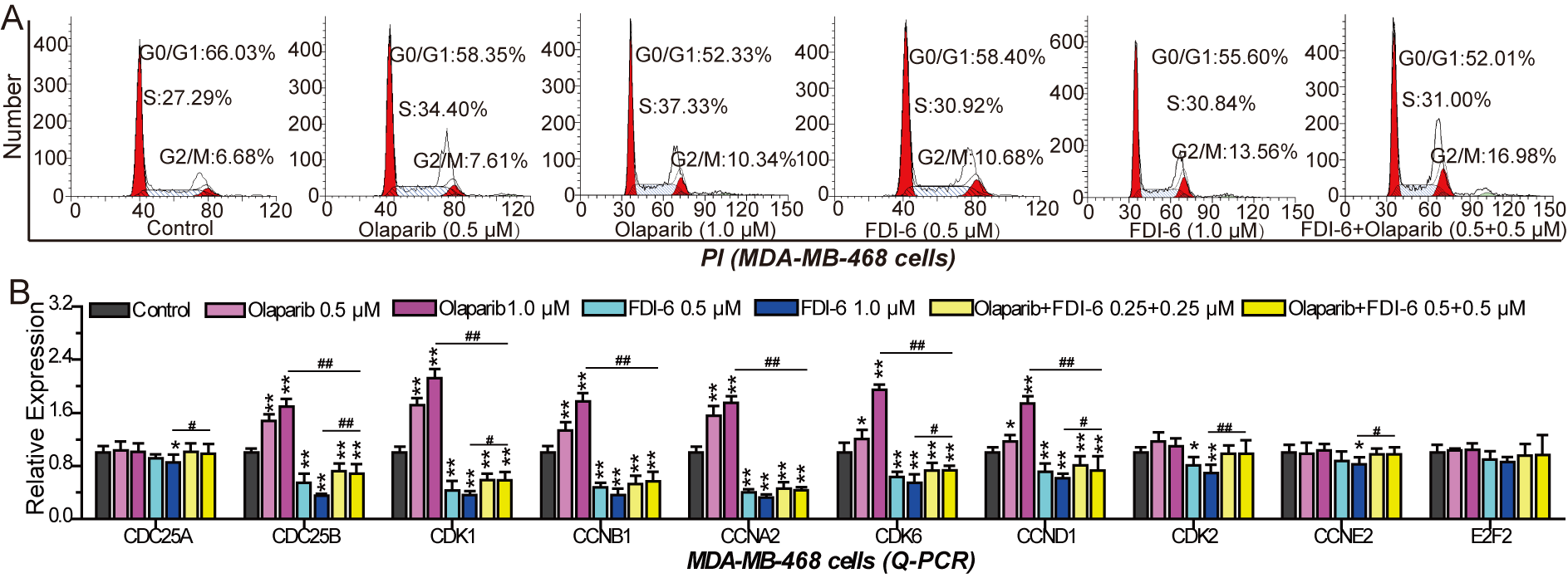
Supplemental Fig. 7. The effects of FDI-6 and/or Olaparib on cell cycle progression.** (A). The effects of FDI-6 and Olaparib on cell cycle progression in MDA-MB-468 cells. (B). The effects of FDI-6 and Olaparib on genes involved in cell cycle control analyzed by Q-PCR in MDA-MB-468 cells. The results from three independent experiments were statistically analyzed using one-way ANOVA: ^*^P<0.05, ^**^P<0.01 compared with the control; ^#^P<0.05, ^##^P<0.01 compared with the FDI-6/Olaparib (0.5 + 0.5 μM) combined group.

**Supplemental Fig. 8**

**
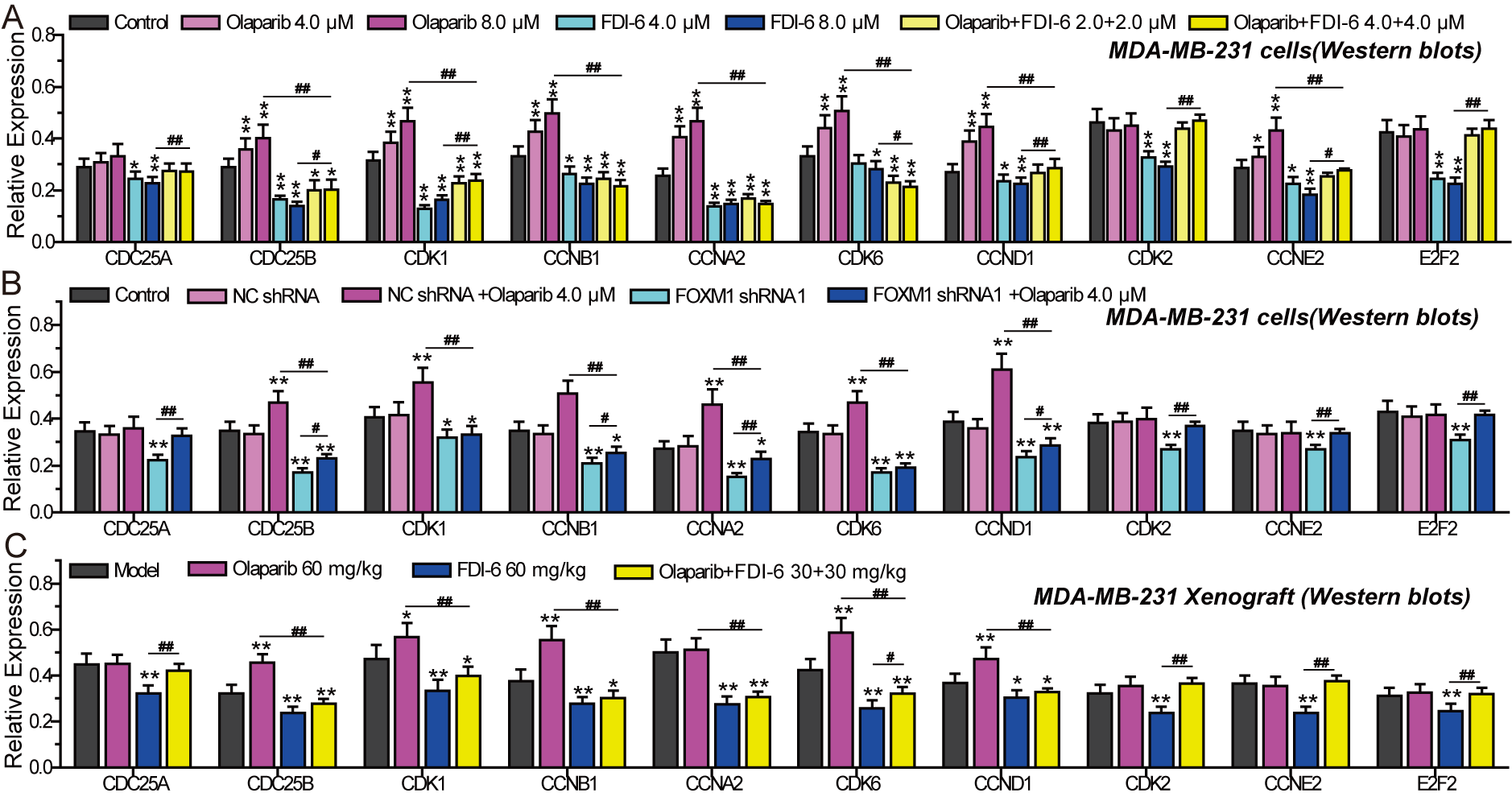
Supplemental Fig. 8. FDI-6 impairs Olaparib-induced expression of proteins involved in cell cycle control.** (A). The effects of FDI-6 and Olaparib on the expression of proteins involved in cell cycle control analyzed by Western blots *in vitro*. (B). The effects of FOXM1 shRNA and Olaparib on the expression of proteins involved in cell cycle control analyzed by Western blots. (C). The effects of FDI-6 and Olaparib on the expression of proteins involved in cell cycle control analyzed by Western blots *in vivo*. The results from three independent experiments were statistically analyzed using one-way ANOVA: ^*^P<0.05, ^**^P<0.01 compared with the control; ^#^P<0.05, ^##^P<0.01 compared with the FDI-6/Olaparib (4.0 + 4.0 μM or 30 + 30 mg/kg) combined group.

**Supplemental Fig. 9**


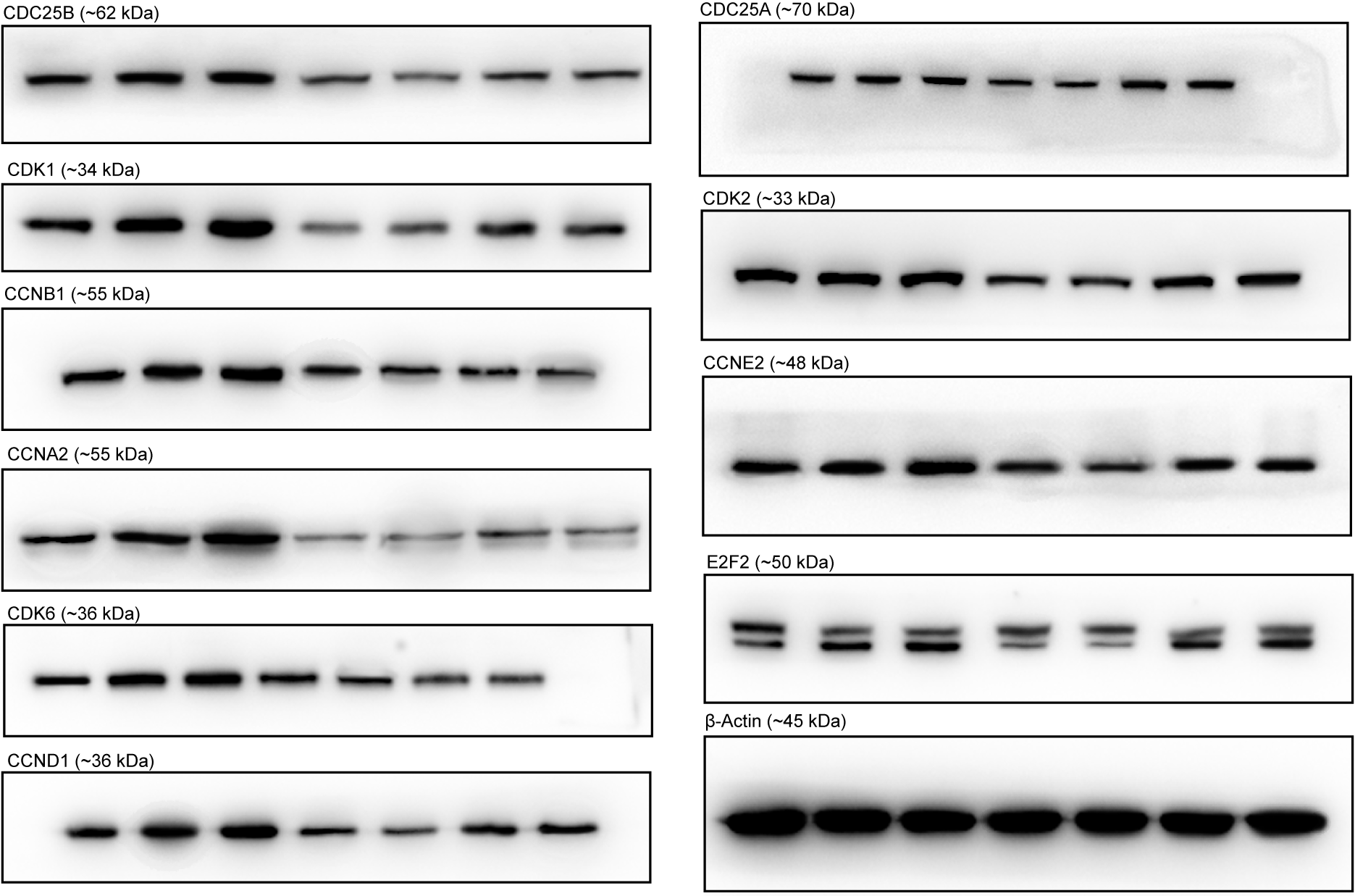


**Supplemental Fig. 9. Full length images of CDC25B, CDC25A, CDK1, CDK2, CCNB1, CCNE2, CCNA2, E2F2, CDK6, CCND1 and β-Actin in MDA-MB-231 cells.** The dilution ratios of all primary antibodies are 1:1000.

**Supplemental Fig. 10**


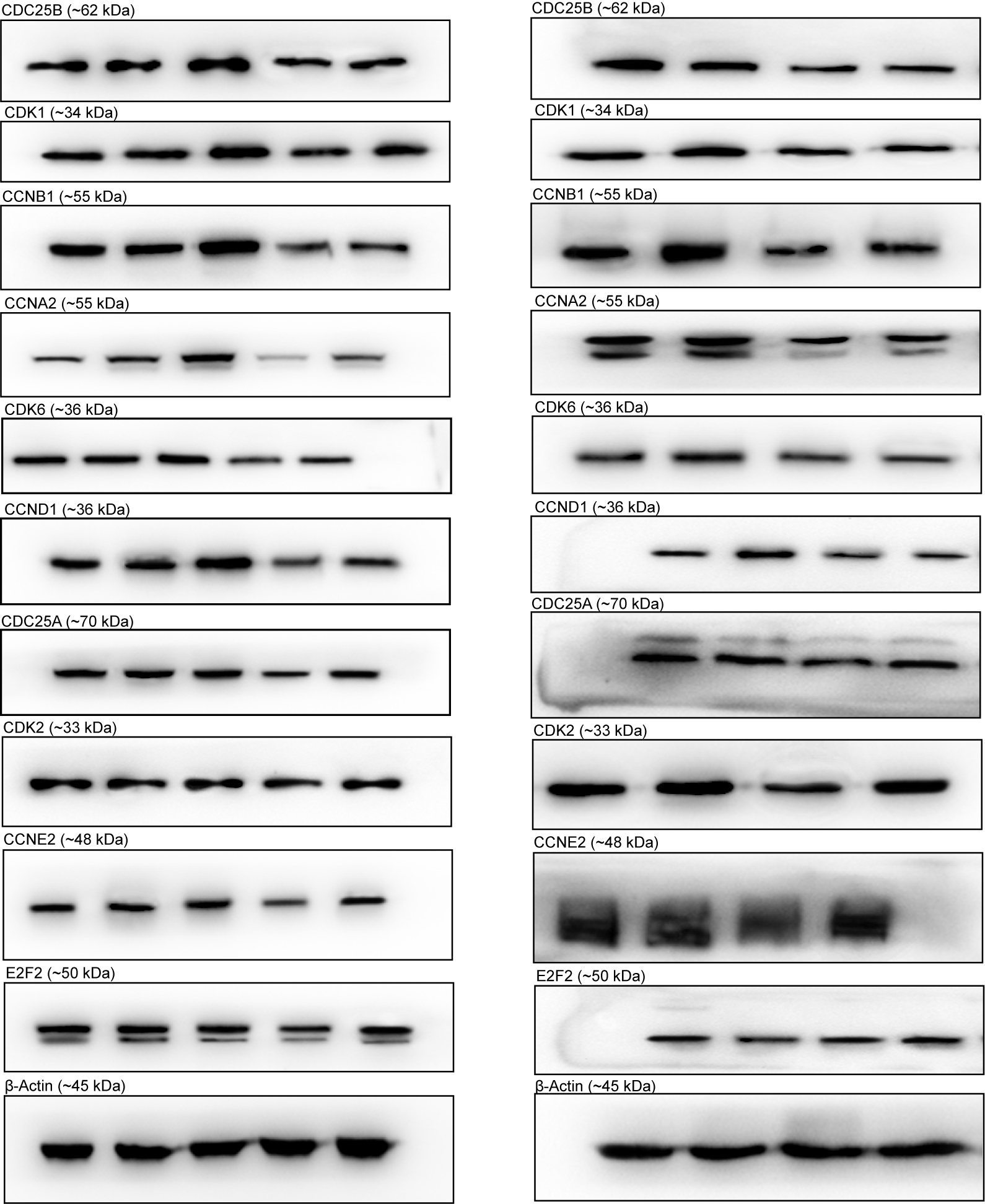


**Supplemental Fig. 10. Full length images of CDC25B, CDC25A, CDK1, CDK2, CCNB1, CCNE2, CCNA2, E2F2, CDK6, CCND1 and β-Actin in MDA-MB-231 cells and MDA-MB-231 xenografts.** The dilution ratios of all primary antibodies are 1:1000.

**Supplemental Fig. 11**

**
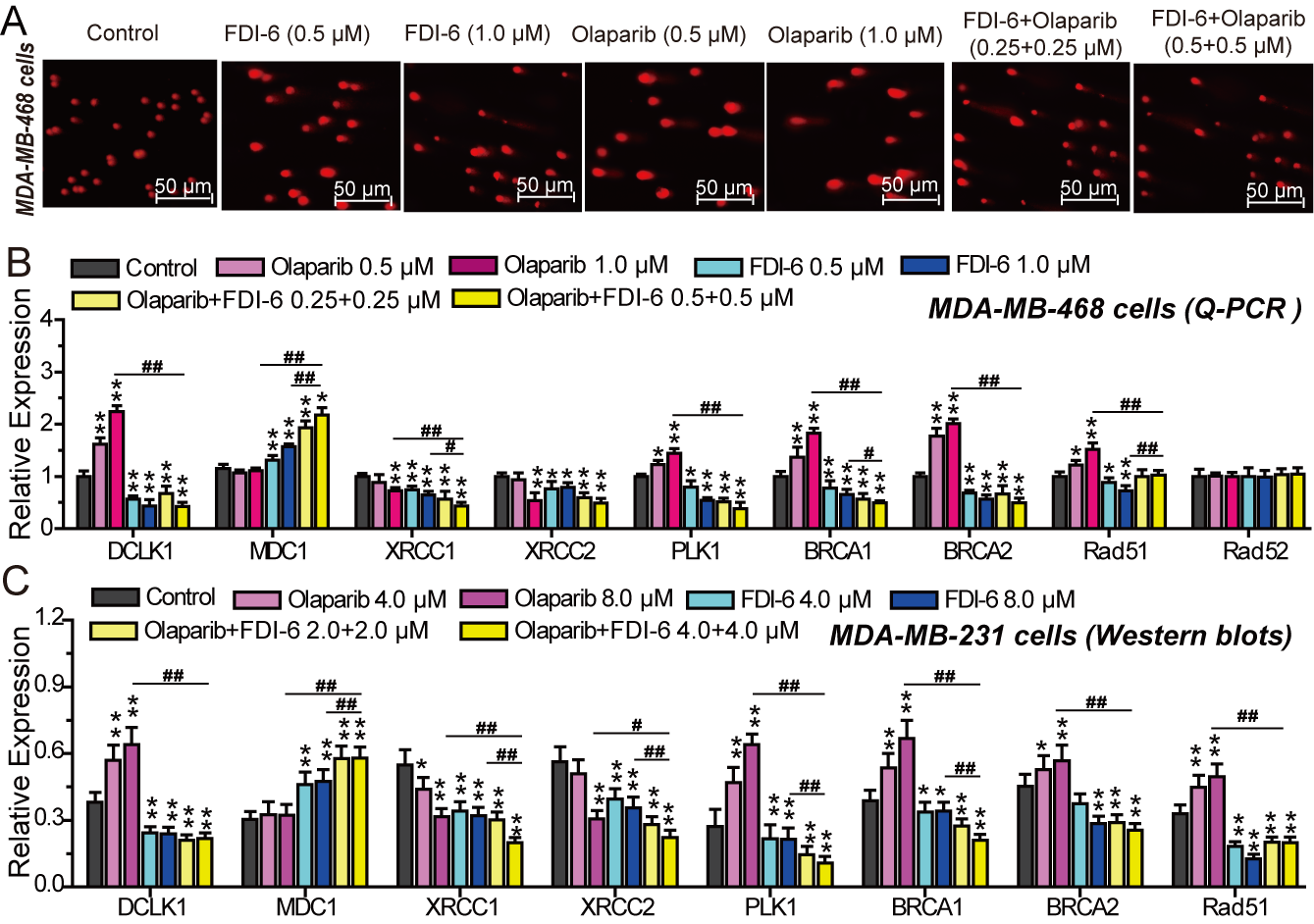
**

**Supplemental Fig. 11. FDI-6 and Olaparib synergistically promote DNA damage *in vitro*.** (A). The extent of DNA damage detected by alkaline comet assay in MDA-MB-468 cells. (B). The effects of FDI-6 and Olaparib on the expression of DNA repair-related genes analyzed by Q-PCR in MDA-MB-468 cells. (C). The effects of FDI-6 and Olaparib on the expression of DNA repair-related proteins analyzed by Western blots in MDA-MB-231 cells. The results from three independent experiments were statistically analyzed using one-way ANOVA: ^*^P<0.05, ^**^P<0.01 compared with control; ^#^P<0.05, ^##^P<0.01 compared with FDI-6/Olaparib combined group (4.0+4.0 μM in MDA-MB-231 cells; 0.5+0.5 μM in MDA-MB-468 cells).

**Supplemental Fig. 12**

**
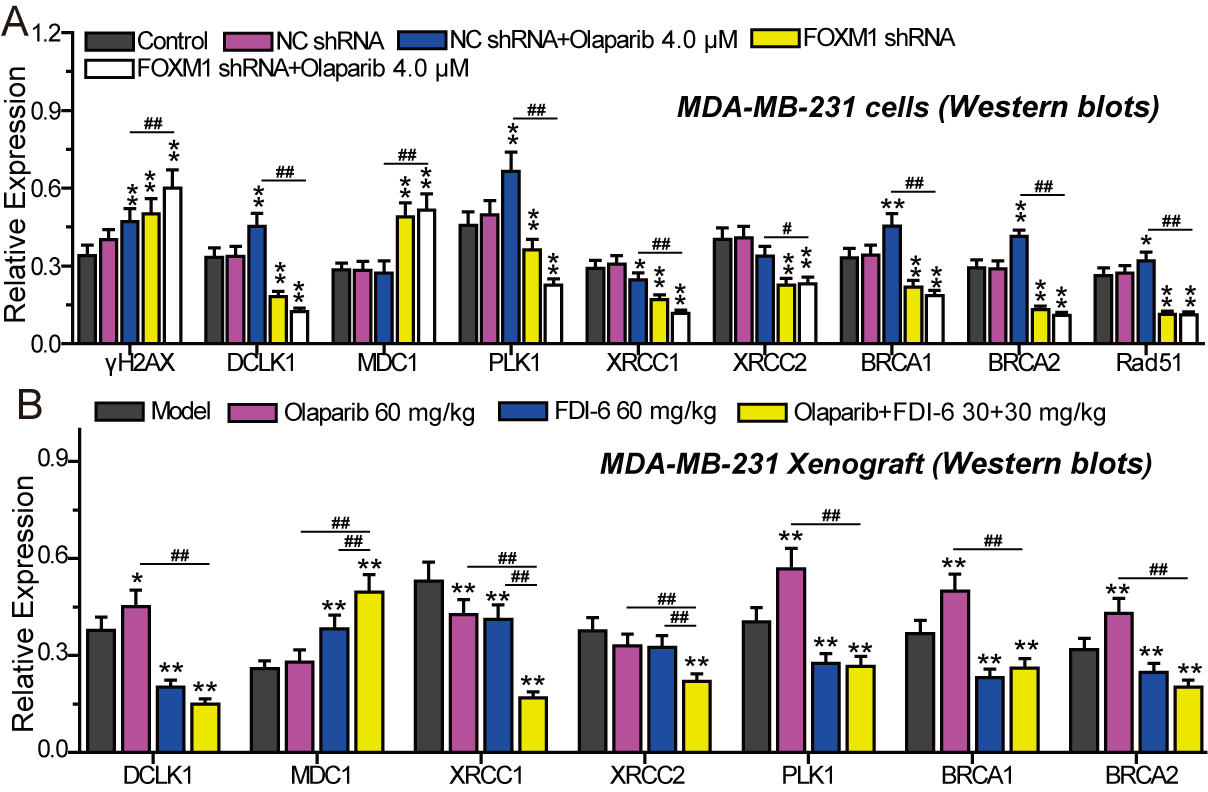
**

**Supplemental Fig. 12. FOXM1 inhibition blocks the expression of DNA repair-related genes.** (A). The effects of FOXM1 shRNA and Olaparib on the expression of DNA repair-related proteins *in vitro* analyzed by Western blots. (B). The effects of FDI-6 and Olaparib on the expression of proteins involved in DNA repair *in vivo* analyzed by Western blots. The results from three independent experiments were statistically analyzed using one-way ANOVA: ^*^P<0.05, ^**^P<0.01 compared with the control; ^#^P<0.05, ^##^P<0.01 compared with the combined group (FDI-6/Olaparib: 30+30 mg/kg; FOXM1 shRNA1/Olaparib: FOXM1 shRNA1+Olaparib 4.0 μM).

**Supplemental Fig. 13**


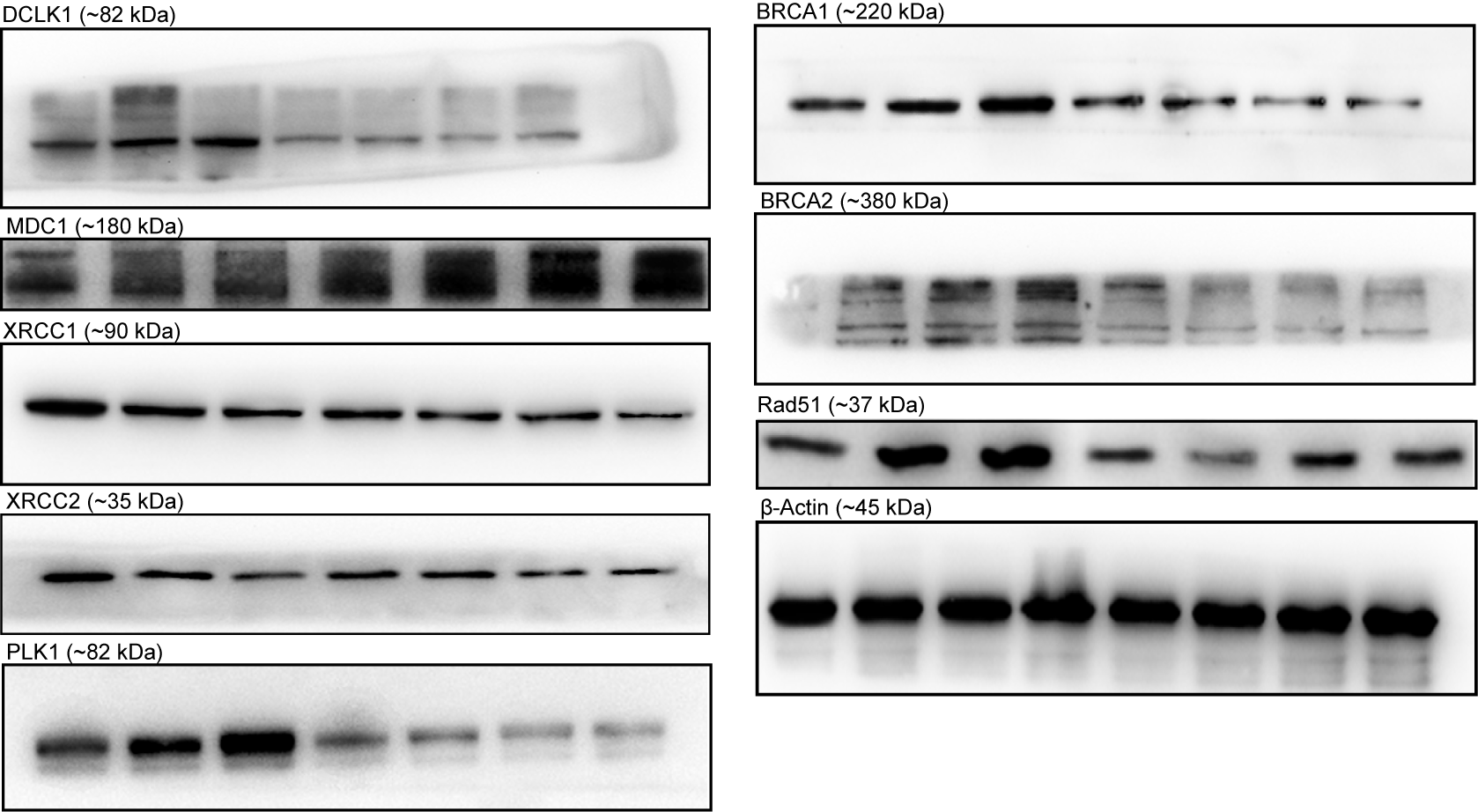


**Supplemental Fig. 13. Full length images of DCLK1, MDC1, XRCC1, XRCC2, PLK1, BRCA1, BRCA2, RAD51 and β-Actin in MDA-MB-231 cells.** The dilution ratios of all primary antibodies are 1:1000.

**Supplemental Fig. 14**


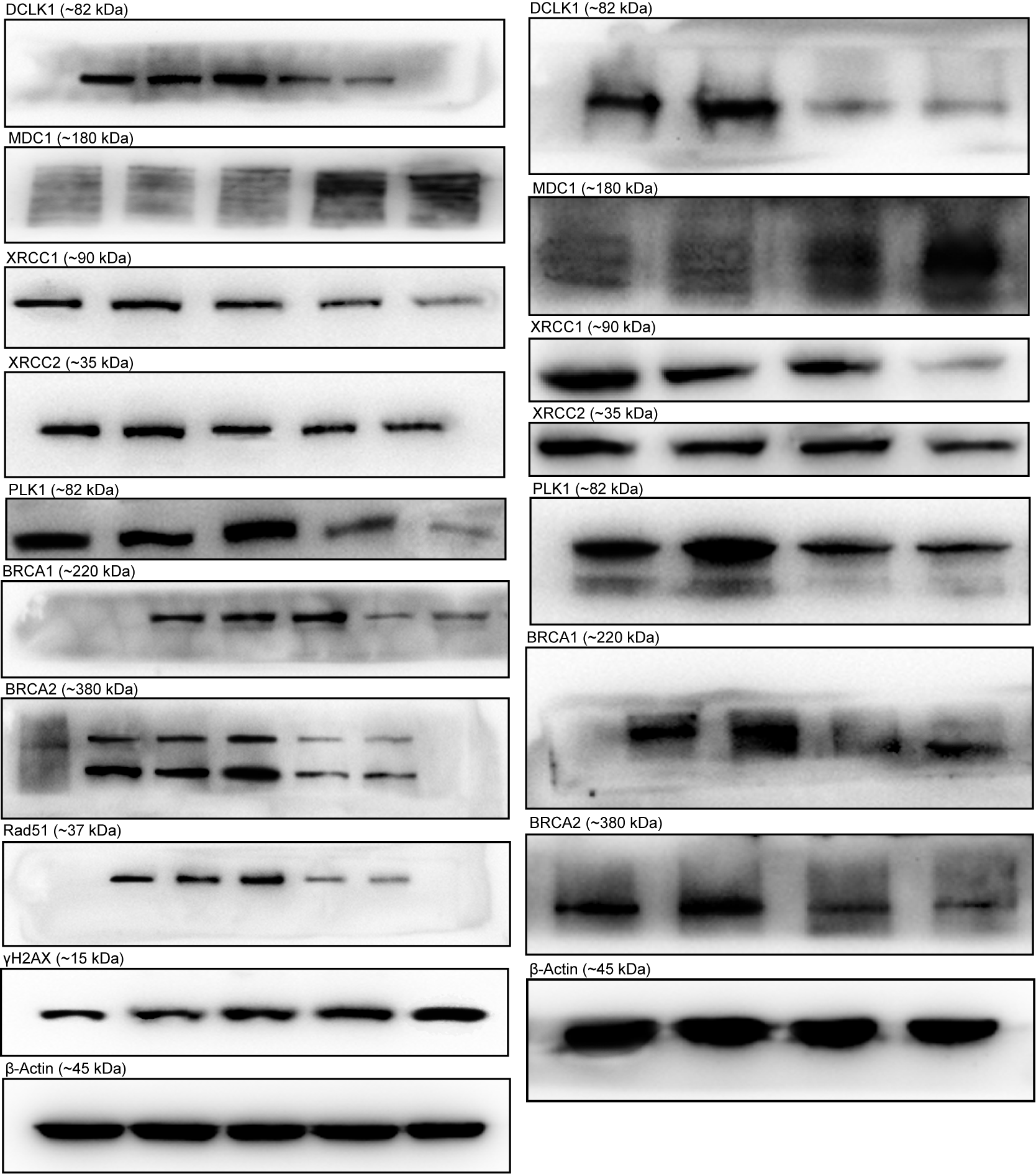


**Supplemental Fig. 14. Full length images of DCLK1, MDC1, XRCC1, XRCC2, PLK1, BRCA1, BRCA2, RAD51 γH2AX and β-Actin in MDA-MB-231 cells and MDA-MB-231 xenografts.** The dilution ratios of all primary antibodies are 1:1000.
