# Supplementary material for: FDI-6 inhibits the expression and function of FOXM1 to sensitize BRCA-proficient triple-negative breast cancer cells to Olaparib by regulating cell cycle progression and DNA damage repair
Source: Cell Death Dis. 2021 Dec 8;12(12):1138. doi: 10.1038/s41419-021-04434-9 (PMC8654856; doi:10.1038/s41419-021-04434-9)
Supplement: Supplementary file 2 — Supplementary Figure Legend [file 41419_2021_4434_MOESM2_ESM.doc]

**Supplementary Figure Legends**

**Supplemental Fig. 1. Differential expression of PARP1 and FOXM1 in the TCGA project.** Differential expression of PARP1 (A) and FOXM1 (B) between tumor tissues and adjacent tissues in the TCGA project. Red represents the expression of genes in tumor tissues is higher than in adjacent normal tissues, Green represents the expression of genes in tumor tissues is lower than in adjacent normal tissues.

**Supplemental Fig. 2. Full length images of FOXM1, PARP1, PARP2 Histone H3 and β-Actin in four TNBC cell lines MDA-MB-231, MDA-MB-468, MDA231-LM2 and HCC1937.** The dilution ratios of all primary antibodies are 1:1000.

**Supplemental Fig. 3. FDI-6 and Olaparib synergistically inhibit the growth of MDA-MB-231 cells *in vitro* and *in vivo*.** (A). Percentage of apoptotic cells in MDA-MB-231 cells and MDA-MB-468 cells. (B). The mice model for the effects of FDI-6 and/or Olaparib on the growth of MDA-MB-231 tumor xenografts. The results from three independent experiments were statistically analyzed using one-way ANOVA: ^*^P<0.05, ^**^P<0.01 compared with the control; ^#^P<0.05, ^##^P<0.01 compared with the FDI-6/Olaparib (4.0 + 4.0 μM or 0.25 + 0.25 μM) combined group.

**Supplemental Fig. 4. FDI-6 and Olaparib synergistically inhibit the expression of FOXM1, PARP1 and PARP2 *in vitro* and *in vivo*.** (A). The expression of PARP1, PARP2 and FOXM1 *in vitro* analyzed by Q-PCR. (B). The expression of PARP1, PARP2 and FOXM1 in Cytoplasm analyzed by Western bolts. (C). The expression of PARP1, PARP2 and FOXM1 *in vitro* analyzed by Western bolts. The results from three independent experiments were statistically analyzed using one-way ANOVA: ^*^P<0.05, ^**^P<0.01 compared with the control; ^#^P<0.05, ^##^P<0.01 compared with the FDI-6/Olaparib (4.0 + 4.0 μM or 0.5 + 0.5 μM) combined group.

**Supplemental Fig. 5. Full length images of FOXM1, PARP1, PARP2 Histone H3 and β-Actin in MDA-MB-231 cells treated with FDI-6, Olaparib or their combination.** The dilution ratios of all primary antibodies are 1:1000.

**Supplemental Fig. 6. Full length images of FOXM1, PARP1 and β-Actin in MDA-MB-231 xenograft treated with FDI-6, Olaparib or their combination.** The dilution ratios of all primary antibodies are 1:1000.

**Supplemental Fig. 7. The effects of FDI-6 and/or Olaparib on cell cycle progression.** (A). The effects of FDI-6 and Olaparib on cell cycle progression in MDA-MB-468 cells. (B). The effects of FDI-6 and Olaparib on genes involved in cell cycle control analyzed by Q-PCR in MDA-MB-468 cells. The results from three independent experiments were statistically analyzed using one-way ANOVA: ^*^P<0.05, ^**^P<0.01 compared with the control; ^#^P<0.05, ^##^P<0.01 compared with the FDI-6/Olaparib (0.5 + 0.5 μM) combined group.

**Supplemental Fig. 8. FDI-6 impairs Olaparib-induced expression of proteins involved in cell cycle control.** (A). The effects of FDI-6 and Olaparib on the expression of proteins involved in cell cycle control analyzed by Western blots *in vitro*. (B). The effects of FOXM1 shRNA and Olaparib on the expression of proteins involved in cell cycle control analyzed by Western blots. (C). The effects of FDI-6 and Olaparib on the expression of proteins involved in cell cycle control analyzed by Western blots *in vivo*. The results from three independent experiments were statistically analyzed using one-way ANOVA: ^*^P<0.05, ^**^P<0.01 compared with the control; ^#^P<0.05, ^##^P<0.01 compared with the FDI-6/Olaparib (4.0 + 4.0 μM or 30 + 30 mg/kg) combined group.

**Supplemental Fig. 9. Full length images of CDC25B, CDC25A, CDK1, CDK2, CCNB1, CCNE2, CCNA2, E2F2, CDK6, CCND1 and β-Actin in MDA-MB-231 cells.** The dilution ratios of all primary antibodies are 1:1000.

**Supplemental Fig. 10. Full length images of CDC25B, CDC25A, CDK1, CDK2, CCNB1, CCNE2, CCNA2, E2F2, CDK6, CCND1 and β-Actin in MDA-MB-231 cells and MDA-MB-231 xenografts.** The dilution ratios of all primary antibodies are 1:1000.

**Supplemental Fig. 11. FDI-6 and Olaparib synergistically promote DNA damage *in vitro*.** (A). The extent of DNA damage detected by alkaline comet assay in MDA-MB-468 cells. (B). The effects of FDI-6 and Olaparib on the expression of DNA repair-related genes analyzed by Q-PCR in MDA-MB-468 cells. (C). The effects of FDI-6 and Olaparib on the expression of DNA repair-related proteins analyzed by Western blots in MDA-MB-231 cells. The results from three independent experiments were statistically analyzed using one-way ANOVA: ^*^P<0.05, ^**^P<0.01 compared with control; ^#^P<0.05, ^##^P<0.01 compared with FDI-6/Olaparib combined group (4.0+4.0 μM in MDA-MB-231 cells; 0.5+0.5 μM in MDA-MB-468 cells).

**Supplemental Fig. 12. FOXM1 inhibition blocks the expression of DNA repair-related genes.** (A). The effects of FOXM1 shRNA and Olaparib on the expression of DNA repair-related proteins *in vitro* analyzed by Western blots. (B). The effects of FDI-6 and Olaparib on the expression of proteins involved in DNA repair *in vivo* analyzed by Western blots. The results from three independent experiments were statistically analyzed using one-way ANOVA: ^*^P<0.05, ^**^P<0.01 compared with the control; ^#^P<0.05, ^##^P<0.01 compared with the combined group (FDI-6/Olaparib: 30+30 mg/kg; FOXM1 shRNA1/Olaparib: FOXM1 shRNA1+Olaparib 4.0 μM).

**Supplemental Fig. 13. Full length images of DCLK1, MDC1, XRCC1, XRCC2, PLK1, BRCA1, BRCA2, RAD51 and β-Actin in MDA-MB-231 cells.** The dilution ratios of all primary antibodies are 1:1000.

**Supplemental Fig. 14. Full length images of DCLK1, MDC1, XRCC1, XRCC2, PLK1, BRCA1, BRCA2, RAD51 γH2AX and β-Actin in MDA-MB-231 cells and MDA-MB-231 xenografts.** The dilution ratios of all primary antibodies are 1:1000.
