# Supplementary material for: FDI-6 inhibits the expression and function of FOXM1 to sensitize BRCA-proficient triple-negative breast cancer cells to Olaparib by regulating cell cycle progression and DNA damage repair
Source: Cell Death Dis. 2021 Dec 8;12(12):1138. doi: 10.1038/s41419-021-04434-9 (PMC8654856; doi:10.1038/s41419-021-04434-9)
Supplement: Supplementary file 17 — Supplementary Table 1 [file 41419_2021_4434_MOESM17_ESM.doc]

**Supplemental Tables**

**Supplemental Table 1. Sequences for shRNA.**

| Name | Sequence |
| --- | --- |
| NC shRNA | CCGGCATTCTCCGAACGTGTCACGTCTCGAGACGTGACACGTTCGGAGAATTTTTG |
| FOXM1 shRNA1 | CCGGCAGCTGGGATCAAGATTATTACTCGAGTAATAATCTTGATCCCAGCTGTTTTTG |
| FOXM1 shRNA2 | CCGGGCGGCCACCCTACTCTTACATCTCGAGATGTAAGAGTAGGGTGGCCGCTTTTTG |
| FOXM1 shRNA3 | CCGGCAGGCTGCACTATCAACAATACTCGAGTATTGTTGATAGTGCAGCCTGTTTTTG |
