# Supplementary material for: FDI-6 inhibits the expression and function of FOXM1 to sensitize BRCA-proficient triple-negative breast cancer cells to Olaparib by regulating cell cycle progression and DNA damage repair
Source: Cell Death Dis. 2021 Dec 8;12(12):1138. doi: 10.1038/s41419-021-04434-9 (PMC8654856; doi:10.1038/s41419-021-04434-9)
Supplement: Supplementary file 18 — Supplementary Table 2 [file 41419_2021_4434_MOESM18_ESM.doc]

**Supplemental Tables**

**Supplemental Table 2.** Primer sequences for genes in Q-PCR.

| **Name** | **Sense (5’-3’)** | **Antisense (5’-3’)** |
| --- | --- | --- |
| PARP1 | ACACAATGCGTATGACTTGGA | CCGTGCCACAGCAATCTTCG |
| PARP2 | ACTATGCCACCAATACTCAGG | CTGTACCCGAAGATCTAGCTG |
| FOXM1 | ACCCAAACCAGCTATGATGCC | TCTCCCGTTTCTGCTCGCAAA |
| DCLK1 | GCAAAGAGCACATGATCCAGA | AGTTCAGTTGGCACATCCATC |
| MDC1 | TTGCAGCTTCCAGACAACAGGT | TTGGTCTCCTGGTATTGCCCTA |
| XRCC1 | CTCAAGGCAGACACTTACCGAA | AGCACCTCCACGAAAGCTGA |
| XRCC2 | CCTTTTGATTTTGGATAGCCT | GCGATAGTCATTTACAAGCTTC |
| PLK1 | GTACCTGCACCGAAACCGAGT | CCTCTCCCCGTCATATTCGACT |
| BRCA1 | TACTAGGCATAGCACCGTTG | ATGCCTTTGCCAATATTACCTG |
| BRCA2 | TGAAATTAAACGGAAGTTTGC | GAATAAAAGCCCCTAAACCC |
| Rad51 | CCCATTTCACGGTTAGAGCA | CTTTGGCTTCACTAATTCCCT |
| Rad52 | TTGCCACCAGAAACCACAAGC | ATTCCCAGTTTCCTGTTGTGC |
| CDK15 | ATAAATGGACAACTAGTGGCTT | ATTGGCATGTTTCAAACCCTT |
| CDC25A | CCTCCGAGTCAACAGATTCAGG | CTTCAGAGCTGGACTACATCCC |
| CDC25B | ACGCACCTATCCCTGTCTCG | TTCAAACGTCTGCTCCGCCAT |
| CDK1 | ATTTGGAGTATAGGCACCAT | GCCACACTTCATTATTGGGA |
| CCNB1 | TGAGAGCCATCCTAATTGACT | AATTATTCTGCATGAACCGAT |
| CCNA2 | CTGAAATAAGGCACAGACCCAA | ATACAGGGTCTCTGCTCGAA |
| CDK6 | ACTTTCTTCATTCACACCGAGT | AGTTTTATTTGTCCGCTGCT |
| CCND1 | CCATGAACTACCTGGACCGCTT | CTTAGAGGCCACGAACATGCAA |
| CDK2 | GCTTTCTGCCATTCTCATCGG | TGGCTAGTCCAAAGTCTGC |
| CCNE2 | TCCAAGAGTTTGCTTACGTCA | TTTAGGAGCATCTTTAAGAGC |
| E2F2 | CGGCGCATCTATGACATCACCA | CAAACATTCCCCTGCCTACCC |
| GAPDH | GAAACTGTGGCGTGATGGC | CACCACTGACACGTTGGCAG |
