# Supplementary material for: FDI-6 inhibits the expression and function of FOXM1 to sensitize BRCA-proficient triple-negative breast cancer cells to Olaparib by regulating cell cycle progression and DNA damage repair
Source: Cell Death Dis. 2021 Dec 8;12(12):1138. doi: 10.1038/s41419-021-04434-9 (PMC8654856; doi:10.1038/s41419-021-04434-9)
Supplement: Supplementary file 19 — Supplementary Table 3 [file 41419_2021_4434_MOESM19_ESM.doc]

**Supplemental Tables**

**Supplemental Table 3.** The inhibition ratios of FDI-6 against 361 kinases.

| Kinase | % Enzyme Activity | | IC50(M) Control Cmpd | Control Cmpd ID |
| --- | --- | --- | --- | --- |
|  | FDI-6 (1.0 μM) | |  |  |
|  | Data 1 | Data 2 |  |  |
| ABL1 | 105.01 | 102.85 | 7.56E-08 | STAUROSPORINE |
| ABL2/ARG | 113.72 | 112.99 | 2.98E-08 | STAUROSPORINE |
| ACK1 | 107.11 | 106.77 | 6.22E-08 | STAUROSPORINE |
| AKT1 | 98.68 | 92.74 | 2.35E-09 | STAUROSPORINE |
| AKT2 | 102.88 | 100.98 | 1.75E-08 | STAUROSPORINE |
| AKT3 | 114.11 | 106.61 | 3.25E-09 | STAUROSPORINE |
| ALK | 90.18 | 85.13 | 2.36E-09 | STAUROSPORINE |
| ALK1/ACVRL1 | 111.32 | 110.32 | 2.56E-08 | LDN193189 |
| ALK2/ACVR1 | 113.86 | 113.86 | 3.12E-08 | LDN193189 |
| ALK3/BMPR1A | 117.76 | 115.02 | 4.85E-08 | LDN193189 |
| ALK4/ACVR1B | 92.32 | 90.85 | 2.17E-07 | LDN193189 |
| ALK5/TGFBR1 | 108.13 | 106.94 | 5.19E-07 | LDN193189 |
| ALK6/BMPR1B | 100.57 | 100.36 | 1.02E-08 | LDN193189 |
| ARAF | 109.77 | 105.87 | 2.40E-08 | GW5074 |
| ARK5/NUAK1 | 90.82 | 89.44 | 6.61E-10 | STAUROSPORINE |
| ASK1/MAP3K5 | 95.06 | 94.96 | 1.53E-08 | STAUROSPORINE |
| AURORA A | 115.24 | 114.10 | 2.06E-09 | STAUROSPORINE |
| AURORA B | 103.28 | 100.47 | 2.31E-08 | STAUROSPORINE |
| AURORA C | 115.06 | 114.13 | 6.16E-09 | STAUROSPORINE |
| AXL | 102.52 | 101.95 | 4.02E-09 | STAUROSPORINE |
| BLK | 89.95 | 88.55 | 1.28E-09 | STAUROSPORINE |
| BMPR2 | 100.60 | 94.16 | 3.60E-07 | STAUROSPORINE |
| BMX/ETK | 91.00 | 90.82 | 5.21E-09 | STAUROSPORINE |
| BRAF | 83.23 | 80.32 | 4.41E-09 | GW5074 |
| BRK | 84.39 | 83.51 | 3.86E-07 | STAUROSPORINE |
| BRSK1 | 90.26 | 90.05 | 5.86E-10 | STAUROSPORINE |
| BRSK2 | 96.27 | 95.35 | 1.03E-09 | STAUROSPORINE |
| BTK | 95.02 | 89.70 | 1.57E-08 | STAUROSPORINE |
| C-KIT | 87.36 | 86.72 | 7.41E-10 | STAUROSPORINE |
| C-MER | 91.58 | 90.06 | 1.35E-08 | STAUROSPORINE |
| C-MET | 70.86 | 70.81 | 1.26E-07 | STAUROSPORINE |
| C-SRC | 92.88 | 91.68 | 1.83E-09 | STAUROSPORINE |
| CAMK1A | 105.50 | 104.70 | 3.30E-09 | STAUROSPORINE |
| CAMK1b | 102.13 | 101.96 | 4.70E-09 | STAUROSPORINE |
| CAMK1d | 105.15 | 103.58 | 6.56E-10 | STAUROSPORINE |
| CAMK1G | 102.12 | 100.64 | 5.75E-09 | STAUROSPORINE |
| CAMK2a | 98.37 | 94.57 | 7.97E-11 | STAUROSPORINE |
| CAMK2B | 109.96 | 108.55 | 7.98E-11 | STAUROSPORINE |
| CAMK2D | 92.10 | 89.10 | 5.54E-11 | STAUROSPORINE |
| CAMK2G | 84.35 | 81.41 | 1.86E-10 | STAUROSPORINE |
| CAMK4 | 77.24 | 76.97 | 9.44E-08 | STAUROSPORINE |
| CAMKK1 | 103.90 | 102.05 | 7.92E-08 | STAUROSPORINE |
| CAMKK2 | 72.06 | 65.75 | 2.97E-08 | STAUROSPORINE |
| CDC7/DBF4 | 88.56 | 87.34 | 4.72E-08 | STAUROSPORINE |
| CDK1/CYCLIN A | 115.15 | 111.88 | 6.02E-09 | STAUROSPORINE |
| CDK1/CYCLIN B | 92.79 | 91.32 | 2.46E-09 | STAUROSPORINE |
| CDK1/CYCLIN E | 87.05 | 86.79 | 3.39E-09 | STAUROSPORINE |
| CDK14/CYCLIN Y | 91.44 | 90.64 | 1.38E-07 | STAUROSPORINE |
| CDK16/CYCLIN Y | 95.12 | 93.40 | 2.22E-08 | STAUROSPORINE |
| CDK17/CYCLIN Y | 104.14 | 103.27 | 1.68E-08 | STAUROSPORINE |
| CDK18/CYCLIN Y | 88.97 | 84.92 | 1.77E-08 | STAUROSPORINE |
| CDK19/CYCLIN C | 97.34 | 95.76 | 2.78E-07 | STAUROSPORINE |
| CDK2/CYCLIN A | 122.08 | 121.92 | 7.96E-10 | STAUROSPORINE |
| CDK2/CYCLIN A1 | 104.31 | 104.22 | 1.69E-09 | STAUROSPORINE |
| CDK2/CYCLIN E | 98.80 | 97.73 | 2.24E-09 | STAUROSPORINE |
| CDK2/CYCLIN E2 | 94.27 | 92.28 | 2.66E-09 | STAUROSPORINE |
| CDK2/CYCLIN O | 98.12 | 95.43 | 1.35E-09 | STAUROSPORINE |
| CDK3/CYCLIN E | 95.00 | 94.49 | 2.56E-09 | STAUROSPORINE |
| CDK3/CYCLIN E2 | 103.86 | 100.38 | 2.83E-09 | STAUROSPORINE |
| CDK4/CYCLIN D1 | 97.54 | 94.90 | 1.55E-08 | STAUROSPORINE |
| CDK4/CYCLIN D3 | 81.13 | 80.11 | 1.63E-08 | STAUROSPORINE |
| CDK5/P25 | 100.01 | 97.18 | 1.63E-09 | STAUROSPORINE |
| CDK5/P35 | 102.69 | 102.25 | 2.19E-09 | STAUROSPORINE |
| CDK6/CYCLIN D1 | 98.95 | 96.46 | 4.05E-09 | STAUROSPORINE |
| CDK6/CYCLIN D3 | 87.26 | 85.95 | 2.41E-08 | STAUROSPORINE |
| CDK7/CYCLIN H | 106.10 | 102.43 | 6.15E-08 | STAUROSPORINE |
| CDK8/CYCLIN C | 108.26 | 107.11 | 2.07E-10 | STAUROSPORINE |
| CDK9/CYCLIN K | 95.01 | 89.61 | 2.09E-08 | STAUROSPORINE |
| CDK9/cyclin T1 | 86.24 | 84.88 | 4.72E-09 | STAUROSPORINE |
| CDK9/CYCLIN T2 | 104.22 | 102.18 | 3.94E-09 | STAUROSPORINE |
| CHK1 | 95.78 | 93.23 | 6.24E-10 | STAUROSPORINE |
| CHK2 | 98.03 | 97.12 | 6.55E-09 | STAUROSPORINE |
| CK1A1 | 98.56 | 96.07 | 4.41E-06 | STAUROSPORINE |
| CK1A1L | 83.19 | 82.14 | 2.28E-06 | STAUROSPORINE |
| CK1D | 82.28 | 80.11 | 2.17E-07 | D4476 |
| CK1EPSILON | 89.98 | 89.86 | 2.82E-07 | D4476 |
| CK1G2 | 105.55 | 104.71 | 3.78E-06 | STAUROSPORINE |
| CK1G3 | 98.45 | 97.99 | 1.90E-06 | STAUROSPORINE |
| CK2A | 82.23 | 82.02 | 1.95E-07 | GW5074 |
| CK2A2 | 125.45 | 123.06 | 1.22E-06 | STAUROSPORINE |
| CLK1 | 102.10 | 100.22 | 1.03E-08 | STAUROSPORINE |
| CLK2 | 97.17 | 96.80 | 4.58E-09 | STAUROSPORINE |
| CLK4 | 108.59 | 105.58 | 7.51E-08 | STAUROSPORINE |
| COT1/MAP3K8 | 77.53 | 75.94 | 8.70E-06 | RO-31-8220 |
| CSK | 93.31 | 92.44 | 1.44E-08 | STAUROSPORINE |
| CTK/MATK | 90.42 | 86.92 | 1.45E-07 | STAUROSPORINE |
| DAPK1 | 104.13 | 103.46 | 1.59E-08 | STAUROSPORINE |
| DAPK2 | 102.55 | 100.65 | 1.12E-08 | STAUROSPORINE |
| DCAMKL1 | 93.59 | 92.91 | 1.19E-07 | STAUROSPORINE |
| DCAMKL2 | 90.25 | 87.49 | 9.80E-09 | STAUROSPORINE |
| DDR1 | 103.79 | 103.06 | 8.54E-09 | STAUROSPORINE |
| DDR2 | 85.61 | 84.84 | 4.49E-10 | STAUROSPORINE |
| DMPK | 87.35 | 86.45 | 2.41E-08 | STAUROSPORINE |
| DMPK2 | 105.65 | 103.30 | 5.10E-10 | STAUROSPORINE |
| DRAK1/STK17A | 91.09 | 87.26 | 4.18E-08 | STAUROSPORINE |
| DYRK1/DYRK1A | 77.94 | 72.45 | 3.50E-09 | STAUROSPORINE |
| DYRK1B | 95.99 | 95.62 | 2.00E-09 | STAUROSPORINE |
| DYRK2 | 108.45 | 108.35 | 2.59E-07 | STAUROSPORINE |
| DYRK3 | 96.40 | 96.10 | 5.29E-08 | STAUROSPORINE |
| DYRK4 | 90.74 | 88.99 | 3.16E-06 | GW5074 |
| EGFR | 82.47 | 80.76 | 1.39E-07 | STAUROSPORINE |
| EPHA1 | 73.82 | 73.43 | 7.88E-08 | STAUROSPORINE |
| EPHA2 | 112.68 | 106.34 | 6.55E-08 | STAUROSPORINE |
| EPHA3 | 95.14 | 94.95 | 2.75E-08 | STAUROSPORINE |
| EPHA4 | 119.77 | 116.59 | 3.59E-08 | STAUROSPORINE |
| EPHA5 | 100.54 | 97.99 | 6.01E-08 | STAUROSPORINE |
| EPHA6 | 94.77 | 94.30 | 2.79E-08 | STAUROSPORINE |
| EPHA7 | 87.64 | 85.33 | 5.46E-08 | STAUROSPORINE |
| EPHA8 | 106.95 | 104.50 | 1.31E-07 | STAUROSPORINE |
| EPHB1 | 110.12 | 110.07 | 9.15E-08 | STAUROSPORINE |
| EPHB2 | 101.45 | 100.93 | 1.64E-07 | STAUROSPORINE |
| EPHB3 | 93.35 | 93.30 | 1.43E-06 | STAUROSPORINE |
| EPHB4 | 89.38 | 87.48 | 1.99E-07 | STAUROSPORINE |
| ERBB2/HER2 | 107.00 | 105.60 | 1.40E-07 | STAUROSPORINE |
| ERBB4/HER4 | 89.24 | 88.22 | 3.47E-07 | STAUROSPORINE |
| ERK1 | 85.60 | 84.62 | 5.16E-09 | SCH772984 |
| ERK2/MAPK1 | 93.88 | 91.75 | 1.35E-09 | SCH772984 |
| ERK5/MAPK7 | 92.66 | 92.58 | 2.23E-05 | STAUROSPORINE |
| ERK7/MAPK15 | 103.27 | 103.10 | 1.18E-08 | STAUROSPORINE |
| ERN1/IRE1 | 72.70 | 70.11 | 7.45E-08 | STAUROSPORINE |
| ERN2/IRE2 | 111.90 | 109.30 | 6.05E-08 | STAUROSPORINE |
| FAK/PTK2 | 93.27 | 92.77 | 9.99E-09 | STAUROSPORINE |
| FER | 86.52 | 86.49 | 3.20E-10 | STAUROSPORINE |
| FES/FPS | 117.74 | 115.59 | 6.49E-10 | STAUROSPORINE |
| FGFR1 | 94.87 | 92.77 | 4.40E-09 | STAUROSPORINE |
| FGFR2 | 96.57 | 94.73 | 2.57E-09 | STAUROSPORINE |
| FGFR3 | 78.00 | 77.48 | 2.19E-08 | STAUROSPORINE |
| FGFR4 | 107.37 | 96.63 | 5.29E-08 | STAUROSPORINE |
| FGR | 121.76 | 121.59 | 6.38E-10 | STAUROSPORINE |
| FLT1/VEGFR1 | 84.80 | 84.48 | 8.69E-09 | STAUROSPORINE |
| FLT3 | 93.23 | 88.71 | 5.65E-09 | STAUROSPORINE |
| FLT4/VEGFR3 | 93.52 | 93.34 | 2.70E-09 | STAUROSPORINE |
| FMS | 114.63 | 111.56 | 1.17E-09 | STAUROSPORINE |
| FRK/PTK5 | 84.88 | 80.61 | 2.79E-08 | STAUROSPORINE |
| FYN | 94.83 | 93.50 | 8.22E-10 | STAUROSPORINE |
| GCK/MAP4K2 | 101.22 | 98.45 | 4.62E-10 | STAUROSPORINE |
| GLK/MAP4K3 | 109.29 | 108.90 | 1.79E-10 | STAUROSPORINE |
| GRK1 | 85.41 | 82.39 | 5.46E-08 | STAUROSPORINE |
| GRK2 | 96.66 | 95.01 | 1.16E-06 | STAUROSPORINE |
| GRK3 | 93.08 | 91.08 | 6.61E-07 | STAUROSPORINE |
| GRK4 | 117.24 | 116.32 | 1.50E-07 | STAUROSPORINE |
| GRK5 | 107.65 | 103.88 | 6.70E-08 | STAUROSPORINE |
| GRK6 | 118.62 | 117.74 | 6.04E-08 | STAUROSPORINE |
| GRK7 | 74.81 | 74.11 | 3.72E-09 | STAUROSPORINE |
| GSK3a | 99.67 | 99.20 | 5.05E-09 | STAUROSPORINE |
| GSK3b | 94.70 | 93.83 | 5.03E-09 | STAUROSPORINE |
| HASPIN | 104.63 | 102.37 | 2.28E-08 | STAUROSPORINE |
| HCK | 103.71 | 103.38 | 1.77E-09 | STAUROSPORINE |
| HGK/MAP4K4 | 70.74 | 62.24 | 1.02E-09 | STAUROSPORINE |
| HIPK1 | 102.94 | 101.94 | 1.98E-06 | RO-31-8220 |
| HIPK2 | 107.57 | 103.38 | 3.48E-07 | STAUROSPORINE |
| HIPK3 | 96.05 | 91.65 | 1.92E-06 | STAUROSPORINE |
| HIPK4 | 101.48 | 100.64 | 6.00E-07 | STAUROSPORINE |
| HPK1/MAP4K1 | 87.35 | 85.33 | 6.58E-08 | RO-31-8220 |
| IGF1R | 82.06 | 80.72 | 7.64E-08 | STAUROSPORINE |
| IKKA/CHUK | 85.54 | 84.35 | 1.14E-07 | STAUROSPORINE |
| IKKB/IKBKB | 79.96 | 79.13 | 3.17E-07 | STAUROSPORINE |
| IKKE/IKBKE | 97.40 | 96.55 | 4.22E-10 | STAUROSPORINE |
| IR | 103.70 | 102.24 | 2.74E-08 | STAUROSPORINE |
| IRAK1 | 83.24 | 81.10 | 6.35E-08 | STAUROSPORINE |
| IRAK4 | 85.03 | 84.79 | 2.43E-09 | STAUROSPORINE |
| IRR/INSRR | 98.36 | 98.17 | 1.70E-08 | STAUROSPORINE |
| ITK | 117.39 | 117.35 | 2.15E-08 | STAUROSPORINE |
| JAK1 | 94.25 | 93.70 | 8.48E-10 | STAUROSPORINE |
| JAK2 | 92.76 | 92.29 | 2.96E-10 | STAUROSPORINE |
| JAK3 | 100.76 | 100.30 | 1.55E-10 | STAUROSPORINE |
| JNK1 | 92.84 | 91.12 | 8.13E-07 | STAUROSPORINE |
| JNK2 | 114.51 | 110.72 | 2.92E-06 | STAUROSPORINE |
| JNK3 | 70.10 | 66.15 | 4.77E-08 | JNKI VIII |
| KDR/VEGFR2 | 95.23 | 94.97 | 1.99E-08 | STAUROSPORINE |
| KHS/MAP4K5 | 79.35 | 78.47 | 2.75E-10 | STAUROSPORINE |
| KSR1 | 97.48 | 97.15 | 1.32E-05 | STAUROSPORINE |
| KSR2 | 82.54 | 80.64 | 3.19E-06 | STAUROSPORINE |
| LATS1 | 111.49 | 109.80 | 2.26E-08 | STAUROSPORINE |
| LATS2 | 95.17 | 90.60 | 6.73E-09 | STAUROSPORINE |
| LCK | 107.97 | 102.23 | 2.68E-09 | STAUROSPORINE |
| LCK2/ICK | 97.48 | 97.05 | 2.47E-08 | STAUROSPORINE |
| LIMK1 | 96.74 | 96.50 | 1.31E-09 | STAUROSPORINE |
| LIMK2 | 100.69 | 98.16 | 8.01E-08 | STAUROSPORINE |
| LKB1 | 88.43 | 87.29 | 5.82E-08 | STAUROSPORINE |
| LOK/STK10 | 104.05 | 102.81 | 1.04E-07 | RO-31-8220 |
| LRRK2 | 92.78 | 92.27 | 1.29E-08 | STAUROSPORINE |
| LYN | 114.37 | 112.85 | 1.66E-09 | STAUROSPORINE |
| LYN B | 133.34 | 132.30 | 5.64E-09 | STAUROSPORINE |
| MAK | 94.60 | 90.06 | 2.76E-08 | STAUROSPORINE |
| MAPKAPK2 | 75.11 | 74.45 | 1.50E-07 | STAUROSPORINE |
| MAPKAPK3 | 102.15 | 101.95 | 2.84E-06 | STAUROSPORINE |
| MARK1 | 97.50 | 95.69 | 1.09E-10 | STAUROSPORINE |
| MARK2/PAR-1Ba | 96.67 | 96.36 | 1.20E-10 | STAUROSPORINE |
| MARK3 | 99.87 | 97.42 | 1.24E-10 | STAUROSPORINE |
| MARK4 | 84.56 | 84.38 | 4.30E-10 | STAUROSPORINE |
| MAST3 | 102.52 | 101.12 | 1.08E-06 | STAUROSPORINE |
| MASTL | 96.00 | 91.87 | 3.38E-08 | STAUROSPORINE |
| MEK1 | 102.83 | 100.91 | 3.70E-08 | STAUROSPORINE |
| MEK2 | 107.09 | 107.09 | 7.30E-08 | STAUROSPORINE |
| MEK3 | 111.96 | 111.73 | 3.73E-08 | STAUROSPORINE |
| MEK5 | 97.67 | 97.07 | 6.35E-08 | STAUROSPORINE |
| MEKK1 | 114.31 | 113.53 | 8.28E-07 | STAUROSPORINE |
| MEKK2 | 93.49 | 93.01 | 7.89E-08 | STAUROSPORINE |
| MEKK3 | 83.89 | 82.10 | 2.92E-08 | STAUROSPORINE |
| MEKK6 | 102.69 | 102.48 | 5.58E-07 | STAUROSPORINE |
| MELK | 94.30 | 93.00 | 4.22E-10 | STAUROSPORINE |
| MINK/MINK1 | 93.65 | 90.56 | 2.69E-10 | STAUROSPORINE |
| MKK4 | 98.40 | 95.10 | 3.06E-06 | STAUROSPORINE |
| MKK6 | 107.78 | 103.29 | 2.01E-08 | STAUROSPORINE |
| MKK7 | 99.14 | 97.69 | 3.12E-06 | STAUROSPORINE |
| MLCK/MYLK | 104.45 | 103.34 | 3.26E-08 | STAUROSPORINE |
| MLCK2/MYLK2 | 88.30 | 85.29 | 1.64E-08 | STAUROSPORINE |
| MLK1/MAP3K9 | 89.37 | 84.44 | 5.16E-10 | STAUROSPORINE |
| MLK2/MAP3K10 | 113.82 | 105.81 | 3.50E-09 | STAUROSPORINE |
| MLK3/MAP3K11 | 106.73 | 103.96 | 2.08E-09 | STAUROSPORINE |
| MLK4 | 86.85 | 85.86 | 1.92E-06 | STAUROSPORINE |
| MNK1 | 100.40 | 99.90 | 1.12E-07 | STAUROSPORINE |
| MNK2 | 95.13 | 90.39 | 1.90E-08 | STAUROSPORINE |
| MRCKa/CDC42BPA | 104.98 | 104.54 | 7.57E-09 | STAUROSPORINE |
| MRCKB/CDC42BPB | 100.93 | 98.82 | 3.85E-09 | STAUROSPORINE |
| MSK2/RPS6KA4 | 103.80 | 103.59 | 1.76E-09 | STAUROSPORINE |
| MSSK1/STK23 | 77.52 | 74.50 | 1.53E-06 | STAUROSPORINE |
| MST1/STK4 | 110.31 | 106.66 | 1.53E-09 | STAUROSPORINE |
| MST2/STK3 | 98.91 | 98.61 | 4.04E-09 | STAUROSPORINE |
| MST3/STK24 | 95.36 | 94.77 | 7.52E-09 | STAUROSPORINE |
| MST4 | 94.00 | 92.74 | 7.66E-09 | STAUROSPORINE |
| MUSK | 95.04 | 91.17 | 4.68E-09 | STAUROSPORINE |
| MYLK3 | 100.44 | 97.99 | 8.23E-08 | STAUROSPORINE |
| MYLK4 | 78.58 | 77.86 | 3.32E-08 | STAUROSPORINE |
| MYO3A | 88.57 | 86.07 | 2.32E-08 | STAUROSPORINE |
| MYO3b | 100.37 | 97.71 | 8.37E-09 | STAUROSPORINE |
| NEK1 | 104.55 | 102.63 | 2.11E-08 | STAUROSPORINE |
| NEK11 | 104.73 | 103.70 | 1.40E-06 | STAUROSPORINE |
| NEK2 | 104.22 | 103.62 | 2.12E-07 | STAUROSPORINE |
| NEK3 | 104.20 | 92.43 | 1.01E-07 | JNK-IN-7 |
| NEK4 | 121.09 | 120.79 | 1.41E-07 | STAUROSPORINE |
| NEK5 | 83.41 | 83.26 | 2.77E-08 | STAUROSPORINE |
| NEK6 | 86.81 | 85.59 | 1.27E-05 | PKR INHIBITOR |
| NEK7 | 90.76 | 90.51 | 9.71E-06 | PKR INHIBITOR |
| NEK9 | 85.46 | 84.27 | 1.01E-07 | STAUROSPORINE |
| NIM1 | 86.29 | 85.46 | 1.58E-07 | STAUROSPORINE |
| NLK | 92.56 | 90.90 | 5.12E-08 | STAUROSPORINE |
| OSR1/OXSR1 | 96.15 | 95.79 | 7.63E-08 | STAUROSPORINE |
| P38A/MAPK14 | 86.54 | 83.10 | 1.22E-08 | SB202190 |
| P38B/MAPK11 | 90.28 | 88.83 | 2.14E-08 | SB202190 |
| P38D/MAPK13 | 115.90 | 111.11 | 4.62E-07 | STAUROSPORINE |
| P38G | 102.64 | 100.67 | 3.51E-07 | STAUROSPORINE |
| p70S6Kb/RPS6KB2 | 104.56 | 101.41 | 3.26E-09 | STAUROSPORINE |
| PAK1 | 99.84 | 98.41 | 1.49E-10 | STAUROSPORINE |
| PAK2 | 108.67 | 108.19 | 1.36E-09 | STAUROSPORINE |
| PAK3 | 103.80 | 102.45 | 1.68E-10 | STAUROSPORINE |
| PAK4 | 106.24 | 105.53 | 5.44E-09 | STAUROSPORINE |
| PAK5 | 90.13 | 89.74 | 3.68E-09 | STAUROSPORINE |
| PAK6 | 100.79 | 100.55 | 7.93E-09 | STAUROSPORINE |
| PBK/TOPK | 83.47 | 77.58 | 4.27E-08 | STAUROSPORINE |
| PDGFRA | 99.96 | 97.37 | 1.33E-09 | STAUROSPORINE |
| PDGFRB | 91.00 | 86.69 | 1.48E-09 | STAUROSPORINE |
| PDK1/PDPK1 | 101.46 | 98.38 | 1.26E-09 | STAUROSPORINE |
| PHKG1 | 102.15 | 100.30 | 1.63E-09 | STAUROSPORINE |
| PHKG2 | 102.92 | 100.85 | 1.03E-09 | STAUROSPORINE |
| PIM1 | 103.78 | 99.89 | 6.03E-09 | STAUROSPORINE |
| PIM2 | 89.81 | 89.16 | 2.81E-08 | STAUROSPORINE |
| PIM3 | 87.05 | 86.34 | 9.46E-11 | STAUROSPORINE |
| PKA | 78.38 | 76.99 | 2.37E-09 | STAUROSPORINE |
| PKACB | 92.27 | 92.06 | 1.46E-09 | STAUROSPORINE |
| PKACG | 87.03 | 83.12 | 3.29E-09 | STAUROSPORINE |
| PKCa | 113.49 | 112.06 | 3.34E-10 | STAUROSPORINE |
| PKCb1 | 100.45 | 98.96 | 2.34E-09 | STAUROSPORINE |
| PKCb2 | 100.28 | 98.57 | 1.62E-09 | STAUROSPORINE |
| PKCD | 96.95 | 96.45 | 1.79E-10 | STAUROSPORINE |
| PKCEPSILON | 96.50 | 94.15 | 3.28E-10 | STAUROSPORINE |
| PKCETA | 105.23 | 100.47 | 3.59E-10 | STAUROSPORINE |
| PKCG | 92.75 | 91.54 | 2.00E-10 | STAUROSPORINE |
| PKCIOTA | 89.10 | 88.41 | 1.05E-08 | STAUROSPORINE |
| PKCMU/PRKD1 | 101.25 | 98.08 | 1.95E-09 | STAUROSPORINE |
| PKCTHETA | 88.19 | 86.17 | 3.24E-09 | STAUROSPORINE |
| PKCZETA | 99.15 | 96.65 | 6.51E-08 | STAUROSPORINE |
| PKD2/PRKD2 | 87.48 | 85.09 | 1.55E-09 | STAUROSPORINE |
| PKG1A | 83.29 | 80.68 | 2.66E-09 | STAUROSPORINE |
| PKG1B | 85.10 | 81.94 | 1.32E-09 | STAUROSPORINE |
| PKG2/PRKG2 | 94.99 | 89.60 | 2.24E-09 | STAUROSPORINE |
| PKN1/PRK1 | 85.71 | 84.09 | 2.36E-10 | STAUROSPORINE |
| PKN2/PRK2 | 85.02 | 83.20 | 1.44E-09 | STAUROSPORINE |
| PKN3/PRK3 | 97.70 | 96.13 | 8.97E-09 | STAUROSPORINE |
| PLK1 | 93.25 | 92.68 | 2.05E-07 | STAUROSPORINE |
| PLK2 | 79.10 | 77.82 | 1.63E-07 | STAUROSPORINE |
| PLK3 | 108.00 | 107.66 | 6.49E-09 | BI2536 |
| PLK4/SAK | 106.58 | 106.28 | 1.64E-08 | STAUROSPORINE |
| PRKX | 97.81 | 94.52 | 1.82E-09 | STAUROSPORINE |
| PYK2 | 104.09 | 102.03 | 9.69E-09 | STAUROSPORINE |
| RAF1 | 99.21 | 97.55 | 5.71E-09 | GW5074 |
| RET | 95.07 | 93.49 | 2.87E-09 | STAUROSPORINE |
| RIPK4 | 88.40 | 86.65 | 4.51E-07 | STAUROSPORINE |
| ROCK1 | 103.66 | 102.22 | 7.95E-10 | STAUROSPORINE |
| ROCK2 | 110.77 | 108.10 | 2.09E-09 | STAUROSPORINE |
| RON/MST1R | 98.21 | 97.11 | 3.46E-07 | STAUROSPORINE |
| ROS/ROS1 | 100.30 | 98.93 | 1.59E-10 | STAUROSPORINE |
| RSK1 | 87.77 | 87.66 | 1.95E-10 | STAUROSPORINE |
| RSK2 | 98.53 | 98.43 | 1.77E-10 | STAUROSPORINE |
| RSK3 | 94.47 | 87.43 | 2.96E-10 | STAUROSPORINE |
| RSK4 | 84.94 | 84.16 | 1.09E-10 | STAUROSPORINE |
| SBK1 | 92.43 | 91.68 | 8.74E-08 | STAUROSPORINE |
| SGK1 | 101.37 | 99.78 | 9.25E-09 | STAUROSPORINE |
| SGK2 | 111.68 | 106.39 | 1.84E-08 | STAUROSPORINE |
| SGK3/SGKL | 113.58 | 110.75 | 1.44E-07 | STAUROSPORINE |
| SIK1 | 94.85 | 94.81 | 3.96E-09 | STAUROSPORINE |
| SIK2 | 107.80 | 107.46 | 1.20E-09 | STAUROSPORINE |
| SIK3 | 98.67 | 98.41 | 1.87E-09 | STAUROSPORINE |
| SLK/STK2 | 65.49 | 55.28 | 1.24E-08 | STAUROSPORINE |
| SNARK/NUAK2 | 92.77 | 90.19 | 1.50E-09 | STAUROSPORINE |
| SNRK | 108.26 | 104.21 | 3.64E-08 | STAUROSPORINE |
| SRMS | 88.95 | 87.67 | 9.82E-06 | STAUROSPORINE |
| SRPK1 | 100.20 | 98.81 | 6.11E-08 | STAUROSPORINE |
| SRPK2 | 98.20 | 97.79 | 4.99E-07 | STAUROSPORINE |
| SSTK/TSSK6 | 96.42 | 93.72 | 2.22E-07 | STAUROSPORINE |
| STK16 | 97.53 | 92.24 | 1.80E-07 | STAUROSPORINE |
| STK21/CIT | 114.27 | 112.22 | 2.47E-07 | STAUROSPORINE |
| STK22D/TSSK1 | 97.77 | 95.50 | 1.11E-10 | STAUROSPORINE |
| STK25/YSK1 | 99.53 | 98.93 | 2.36E-09 | STAUROSPORINE |
| STK32B/YANK2 | 119.43 | 115.97 | 4.10E-08 | STAUROSPORINE |
| STK32C/YANK3 | 80.85 | 80.34 | 1.29E-07 | STAUROSPORINE |
| STK33 | 92.02 | 85.75 | 2.09E-08 | STAUROSPORINE |
| STK38/NDR1 | 89.32 | 88.25 | 6.56E-10 | STAUROSPORINE |
| STK38L/NDR2 | 101.43 | 99.30 | 1.12E-09 | STAUROSPORINE |
| STK39/STLK3 | 105.49 | 102.46 | 1.62E-08 | STAUROSPORINE |
| SYK | 107.11 | 101.51 | 5.98E-10 | STAUROSPORINE |
| TAK1 | 108.31 | 106.08 | 1.17E-07 | STAUROSPORINE |
| TAOK1 | 96.43 | 96.01 | 7.69E-10 | STAUROSPORINE |
| TAOK2/TAO1 | 91.47 | 90.25 | 5.94E-09 | STAUROSPORINE |
| TAOK3/JIK | 81.89 | 79.89 | 6.05E-09 | STAUROSPORINE |
| TBK1 | 106.73 | 99.41 | 9.66E-10 | STAUROSPORINE |
| TEC | 85.78 | 84.58 | 6.10E-08 | STAUROSPORINE |
| TESK1 | 91.54 | 91.51 | 1.83E-07 | STAUROSPORINE |
| TESK2 | 90.02 | 87.95 | 1.32E-05 | STAUROSPORINE |
| TGFBR2 | 90.07 | 87.81 | 1.14E-07 | LDN193189 |
| TIE2/TEK | 94.63 | 94.44 | 7.68E-08 | STAUROSPORINE |
| TLK1 | 101.33 | 99.95 | 4.75E-08 | STAUROSPORINE |
| TLK2 | 93.06 | 91.90 | 4.02E-09 | STAUROSPORINE |
| TNIK | 98.30 | 97.39 | 3.51E-10 | STAUROSPORINE |
| TNK1 | 77.76 | 76.28 | 4.96E-09 | STAUROSPORINE |
| TRKA | 99.14 | 98.41 | 3.05E-09 | STAUROSPORINE |
| TRKB | 91.34 | 88.49 | 4.47E-10 | STAUROSPORINE |
| TRKC | 99.81 | 99.68 | 2.08E-10 | STAUROSPORINE |
| TSSK2 | 93.60 | 93.45 | 6.52E-09 | STAUROSPORINE |
| TSSK3/STK22C | 93.97 | 92.92 | 5.98E-09 | STAUROSPORINE |
| TTBK1 | 109.75 | 105.17 | 1.63E-05 | SB202190 |
| TTBK2 | 96.30 | 95.07 | 4.19E-06 | SB202190 |
| TXK | 92.34 | 92.10 | 3.90E-08 | STAUROSPORINE |
| TYK1/LTK | 103.77 | 99.57 | 4.81E-08 | STAUROSPORINE |
| TYK2 | 82.12 | 80.86 | 4.02E-10 | STAUROSPORINE |
| TYRO3/SKY | 113.76 | 110.75 | 3.35E-09 | STAUROSPORINE |
| ULK1 | 87.32 | 83.81 | 6.32E-09 | STAUROSPORINE |
| ULK2 | 103.59 | 102.95 | 2.96E-09 | STAUROSPORINE |
| ULK3 | 90.10 | 89.65 | 3.75E-09 | STAUROSPORINE |
| VRK1 | 82.45 | 81.77 | 4.59E-07 | RO-31-8220 |
| VRK2 | 88.26 | 87.20 | 1.18E-05 | RO-31-8220 |
| WEE1 | 73.14 | 72.76 | 6.16E-08 | WEE-1 INHIBITOR |
| WNK1 | 93.38 | 92.23 | 1.98E-05 | STAUROSPORINE |
| WNK2 | 79.44 | 77.35 | 6.20E-06 | STAUROSPORINE |
| WNK3 | 100.27 | 99.93 | 1.43E-06 | WEE-1 INHIBITOR |
| YES/YES1 | 103.25 | 100.41 | 9.14E-10 | STAUROSPORINE |
| YSK4/MAP3K19 | 98.99 | 92.24 | 1.08E-08 | STAUROSPORINE |
| ZAK/MLTK | 101.85 | 101.12 | 1.41E-06 | GW5074 |
| ZAP70 | 100.24 | 99.07 | 1.36E-08 | STAUROSPORINE |
| ZIPK/DAPK3 | 101.36 | 100.39 | 6.28E-09 | STAUROSPORINE |
