# Supplementary material for: FDI-6 inhibits the expression and function of FOXM1 to sensitize BRCA-proficient triple-negative breast cancer cells to Olaparib by regulating cell cycle progression and DNA damage repair
Source: Cell Death Dis. 2021 Dec 8;12(12):1138. doi: 10.1038/s41419-021-04434-9 (PMC8654856; doi:10.1038/s41419-021-04434-9)
Supplement: Supplementary file 20 — Supplementary Table 4 [file 41419_2021_4434_MOESM20_ESM.doc]

**Supplemental Tables**

**Supplemental Table 4. DEGs in control vs FDI-6 treated group analyzed by RNA sequencing.**

| ID | Symbol | log_2_(fc) | P Value | FDR |
| --- | --- | --- | --- | --- |
| ENSG00000205923 | CEMP1 | 5.5046204 | 7.15E-05 | 0.000530735 |
| ENSG00000132207 | SLX1A | 5.4093909 | 0.000666232 | 0.003724187 |
| ENSG00000138395 | CDK15 | 3.1154772 | 1.38E-10 | 3.11E-09 |
| ENSG00000153404 | PLEKHG4B | 2.9385995 | 2.17E-05 | 0.000183443 |
| ENSG00000138622 | HCN4 | 2.7327161 | 1.69E-07 | 2.31E-06 |
| ENSG00000114812 | VIPR1 | 2.7202068 | 6.69E-169 | 2.92E-165 |
| ENSG00000140465 | CYP1A1 | 2.7147406 | 9.13E-115 | 2.39E-111 |
| ENSG00000184937 | WT1 | 2.6421064 | 0.004971273 | 0.020466638 |
| ENSG00000186628 | FSD2 | 2.4594316 | 0.009372494 | 0.034731019 |
| ENSG00000060566 | CREB3L3 | 2.428237 | 9.31E-07 | 1.08E-05 |
| ENSG00000103888 | CEMIP | 2.2983413 | 1.47E-07 | 2.02E-06 |
| ENSG00000138061 | CYP1B1 | 2.2829056 | 0 | 0 |
| ENSG00000197632 | SERPINB2 | 2.2672984 | 3.66E-60 | 2.28E-57 |
| ENSG00000189057 | FAM111B | 2.2576071 | 4.19E-31 | 5.90E-29 |
| ENSG00000121858 | TNFSF10 | 2.2307016 | 5.41E-44 | 1.51E-41 |
| ENSG00000268975 | MIA-RAB4B | 2.2209992 | 9.41E-05 | 0.00067697 |
| ENSG00000165495 | PKNOX2 | 2.1926451 | 0.013010983 | 0.045667505 |
| ENSG00000280893 | AC009133.6 | 2.103951 | 9.24E-06 | 8.65E-05 |
| ENSG00000092470 | WDR76 | 2.0990294 | 5.37E-19 | 3.17E-17 |
| ENSG00000148735 | PLEKHS1 | 2.0285692 | 0.0087332 | 0.032864343 |
| ENSG00000175322 | ZNF519 | 2.0204641 | 6.38E-06 | 6.19E-05 |
| ENSG00000143858 | SYT2 | 1.9919627 | 5.38E-09 | 9.52E-08 |
| ENSG00000118513 | MYB | 1.9886847 | 0.00397823 | 0.017065198 |
| ENSG00000085840 | ORC1 | 1.9686959 | 3.94E-14 | 1.38E-12 |
| ENSG00000198125 | MB | 1.9391748 | 0.003628047 | 0.015853935 |
| ENSG00000007968 | E2F2 | 1.9130167 | 3.03E-06 | 3.15E-05 |
| ENSG00000196611 | MMP1 | 1.8774116 | 5.00E-47 | 1.60E-44 |
| ENSG00000159307 | SCUBE1 | 1.8744691 | 0.001938357 | 0.009281992 |
| ENSG00000128408 | RIBC2 | 1.8008999 | 0.007645008 | 0.029342845 |
| ENSG00000187554 | TLR5 | 1.7454272 | 0.008238164 | 0.031280175 |
| ENSG00000100302 | RASD2 | 1.714132 | 3.43E-07 | 4.41E-06 |
| ENSG00000144354 | CDCA7 | 1.6619403 | 5.16E-25 | 5.32E-23 |
| ENSG00000162062 | TEDC2 | 1.6380087 | 0.002217765 | 0.010438945 |
| ENSG00000166106 | ADAMTS15 | 1.6343914 | 4.16E-38 | 8.38E-36 |
| ENSG00000185920 | PTCH1 | 1.6163796 | 1.35E-51 | 5.37E-49 |
| ENSG00000100297 | MCM5 | 1.6127744 | 4.16E-42 | 1.07E-39 |
| ENSG00000143603 | KCNN3 | 1.6088092 | 2.37E-12 | 6.65E-11 |
| ENSG00000198056 | PRIM1 | 1.5818262 | 3.45E-11 | 8.31E-10 |
| ENSG00000175305 | CCNE2 | 1.5754523 | 5.00E-27 | 5.54E-25 |
| ENSG00000164920 | OSR2 | 1.5687066 | 0.003604265 | 0.015771068 |
| ENSG00000169129 | AFAP1L2 | 1.5677552 | 1.25E-34 | 2.15E-32 |
| ENSG00000100628 | ASB2 | 1.556226 | 4.84E-73 | 4.53E-70 |
| ENSG00000114491 | UMPS | 1.5462054 | 0.000104794 | 0.00074499 |
| ENSG00000104738 | MCM4 | 1.5349083 | 2.52E-88 | 4.12E-85 |
| ENSG00000124171 | PARD6B | 1.5011848 | 2.06E-51 | 7.94E-49 |
| ENSG00000239732 | TLR9 | 1.4882865 | 1.45E-07 | 2.00E-06 |
| ENSG00000115687 | PASK | 1.4707469 | 1.32E-08 | 2.21E-07 |
| ENSG00000162654 | GBP4 | 1.4666425 | 5.98E-08 | 9.01E-07 |
| ENSG00000133056 | PIK3C2B | 1.4565141 | 2.45E-13 | 7.87E-12 |
| ENSG00000180616 | SSTR2 | 1.447459 | 0.000116134 | 0.000818885 |
| ENSG00000171408 | PDE7B | 1.4267705 | 1.64E-10 | 3.63E-09 |
| ENSG00000250644 | AC068580.4 | 1.4203889 | 1.04E-80 | 1.23E-77 |
| ENSG00000101412 | E2F1 | 1.4142383 | 1.05E-30 | 1.44E-28 |
| ENSG00000265763 | ZNF488 | 1.4095624 | 0.000193736 | 0.001289475 |
| ENSG00000104081 | BMF | 1.4010691 | 1.72E-39 | 3.81E-37 |
| ENSG00000147614 | ATP6V0D2 | 1.3953772 | 1.10E-09 | 2.15E-08 |
| ENSG00000109971 | HSPA8 | 1.3903778 | 2.16E-232 | 1.41E-228 |
| ENSG00000164379 | FOXQ1 | 1.3752913 | 1.00E-05 | 9.29E-05 |
| ENSG00000146678 | IGFBP1 | 1.3604565 | 1.74E-33 | 2.85E-31 |
| ENSG00000119714 | GPR68 | 1.3501051 | 9.43E-35 | 1.65E-32 |
| ENSG00000100479 | POLE2 | 1.3468981 | 1.09E-11 | 2.81E-10 |
| ENSG00000180884 | ZNF792 | 1.3440538 | 3.30E-12 | 9.09E-11 |
| ENSG00000131153 | GINS2 | 1.3331992 | 2.52E-06 | 2.68E-05 |
| ENSG00000241360 | PDXP | 1.3223362 | 5.44E-12 | 1.46E-10 |
| ENSG00000276043 | UHRF1 | 1.2958629 | 1.32E-46 | 4.12E-44 |
| ENSG00000089723 | OTUB2 | 1.289543 | 7.11E-40 | 1.61E-37 |
| ENSG00000184992 | BRI3BP | 1.2894196 | 2.35E-25 | 2.48E-23 |
| ENSG00000077942 | FBLN1 | 1.2797174 | 2.09E-07 | 2.80E-06 |
| ENSG00000167513 | CDT1 | 1.2771627 | 3.32E-13 | 1.05E-11 |
| ENSG00000136982 | DSCC1 | 1.2620322 | 2.07E-10 | 4.52E-09 |
| ENSG00000092853 | CLSPN | 1.2617209 | 1.58E-22 | 1.37E-20 |
| ENSG00000143476 | DTL | 1.2610729 | 1.21E-37 | 2.36E-35 |
| ENSG00000112118 | MCM3 | 1.2590209 | 1.39E-87 | 1.82E-84 |
| ENSG00000146966 | DENND2A | 1.2466178 | 1.48E-09 | 2.83E-08 |
| ENSG00000181544 | FANCB | 1.2386254 | 2.91E-06 | 3.04E-05 |
| ENSG00000270181 | BIVM-ERCC5 | 1.2374993 | 0.000989526 | 0.005238527 |
| ENSG00000162496 | DHRS3 | 1.2362893 | 4.24E-26 | 4.55E-24 |
| ENSG00000076003 | MCM6 | 1.2357205 | 2.95E-50 | 1.04E-47 |
| ENSG00000132646 | PCNA | 1.2257671 | 2.85E-39 | 6.02E-37 |
| ENSG00000180730 | SHISA2 | 1.2141036 | 3.18E-14 | 1.12E-12 |
| ENSG00000102312 | PORCN | 1.2068752 | 5.06E-25 | 5.26E-23 |
| ENSG00000135723 | FHOD1 | 1.2068246 | 1.56E-29 | 2.04E-27 |
| ENSG00000161939 | RNASEK-C17orf49 | 1.2063413 | 0.003568777 | 0.015652406 |
| ENSG00000204634 | TBC1D8 | 1.2051934 | 1.84E-19 | 1.14E-17 |
| ENSG00000172379 | ARNT2 | 1.1955508 | 1.16E-24 | 1.15E-22 |
| ENSG00000265190 | ANXA8 | 1.1953403 | 1.45E-10 | 3.25E-09 |
| ENSG00000183049 | CAMK1D | 1.1936647 | 1.92E-10 | 4.22E-09 |
| ENSG00000141574 | SECTM1 | 1.1918516 | 6.08E-07 | 7.32E-06 |
| ENSG00000183779 | ZNF703 | 1.1894284 | 6.86E-18 | 3.64E-16 |
| ENSG00000167772 | ANGPTL4 | 1.1885974 | 9.24E-05 | 0.000665154 |
| ENSG00000198945 | L3MBTL3 | 1.1760215 | 1.51E-05 | 0.000133055 |
| ENSG00000136492 | BRIP1 | 1.1582401 | 5.44E-13 | 1.68E-11 |
| ENSG00000105409 | ATP1A3 | 1.1470539 | 0.000322991 | 0.002019391 |
| ENSG00000160949 | TONSL | 1.1398962 | 1.03E-17 | 5.35E-16 |
| ENSG00000062822 | POLD1 | 1.1333005 | 9.42E-20 | 6.09E-18 |
| ENSG00000065328 | MCM10 | 1.1330254 | 1.56E-21 | 1.25E-19 |
| ENSG00000221963 | APOL6 | 1.131167 | 5.96E-53 | 2.52E-50 |
| ENSG00000155760 | FZD7 | 1.1232756 | 2.12E-32 | 3.26E-30 |
| ENSG00000169258 | GPRIN1 | 1.1211091 | 4.41E-09 | 7.92E-08 |
| ENSG00000042062 | RIPOR3 | 1.1172835 | 2.48E-07 | 3.27E-06 |
| ENSG00000164418 | GRIK2 | 1.1102821 | 0.000438514 | 0.002631081 |
| ENSG00000117228 | GBP1 | 1.108289 | 6.24E-21 | 4.64E-19 |
| ENSG00000135750 | KCNK1 | 1.1025979 | 0.000653428 | 0.003671538 |
| ENSG00000163362 | INAVA | 1.0975041 | 7.70E-12 | 2.03E-10 |
| ENSG00000166508 | MCM7 | 1.0966884 | 2.07E-50 | 7.54E-48 |
| ENSG00000158292 | GPR153 | 1.096508 | 0.000205822 | 0.001362298 |
| ENSG00000154188 | ANGPT1 | 1.0909462 | 0.001594944 | 0.007888558 |
| ENSG00000171051 | FPR1 | 1.0874628 | 0.000712201 | 0.003947559 |
| ENSG00000165474 | GJB2 | 1.0851674 | 0.001128044 | 0.005837296 |
| ENSG00000127423 | AUNIP | 1.0819176 | 0.000559971 | 0.003208374 |
| ENSG00000004799 | PDK4 | 1.0803734 | 0.00690274 | 0.026976319 |
| ENSG00000078900 | TP73 | 1.0792267 | 0.001374549 | 0.006924047 |
| ENSG00000100065 | CARD10 | 1.0770808 | 7.61E-15 | 2.86E-13 |
| ENSG00000105486 | LIG1 | 1.0736441 | 4.74E-17 | 2.32E-15 |
| ENSG00000129675 | ARHGEF6 | 1.0730635 | 0.007561487 | 0.029064881 |
| ENSG00000116525 | TRIM62 | 1.066931 | 7.77E-05 | 0.000571831 |
| ENSG00000138439 | FAM117B | 1.061816 | 9.69E-07 | 1.12E-05 |
| ENSG00000102996 | MMP15 | 1.0615178 | 4.39E-13 | 1.37E-11 |
| ENSG00000113368 | LMNB1 | 1.0475316 | 6.36E-20 | 4.22E-18 |
| ENSG00000087510 | TFAP2C | 1.0451609 | 3.44E-14 | 1.21E-12 |
| ENSG00000128694 | OSGEPL1 | 1.0378682 | 1.99E-07 | 2.69E-06 |
| ENSG00000138944 | SHISAL1 | 1.0352755 | 0.000685725 | 0.003815348 |
| ENSG00000173530 | TNFRSF10D | 1.0351101 | 3.82E-20 | 2.59E-18 |
| ENSG00000161395 | PGAP3 | 1.0332586 | 3.53E-06 | 3.60E-05 |
| ENSG00000169598 | DFFB | 1.0250426 | 4.72E-05 | 0.000368751 |
| ENSG00000131459 | GFPT2 | 1.0199582 | 3.23E-59 | 1.92E-56 |
| ENSG00000171604 | CXXC5 | 1.0192967 | 1.65E-14 | 5.97E-13 |
| ENSG00000160256 | FAM207A | 1.0171898 | 0.002647147 | 0.012147371 |
| ENSG00000101230 | ISM1 | 1.0149503 | 3.61E-10 | 7.61E-09 |
| ENSG00000162337 | LRP5 | 1.0125218 | 2.96E-45 | 8.79E-43 |
| ENSG00000113389 | NPR3 | 1.0107264 | 0.004048905 | 0.017317301 |
| ENSG00000011478 | QPCTL | 1.0101992 | 5.77E-11 | 1.37E-09 |
| ENSG00000114268 | PFKFB4 | 1.0076652 | 9.87E-20 | 6.33E-18 |
| ENSG00000108515 | ENO3 | -1.003458 | 1.66E-15 | 6.92E-14 |
| ENSG00000140941 | MAP1LC3B | -1.003779 | 1.99E-62 | 1.45E-59 |
| ENSG00000058335 | RASGRF1 | -1.004419 | 5.47E-22 | 4.42E-20 |
| ENSG00000164400 | CSF2 | -1.007418 | 4.85E-24 | 4.70E-22 |
| ENSG00000041982 | TNC | -1.0088 | 1.45E-34 | 2.47E-32 |
| ENSG00000186523 | FAM86B1 | -1.012198 | 1.42E-06 | 1.58E-05 |
| ENSG00000136014 | USP44 | -1.0129 | 0.009831103 | 0.036174481 |
| ENSG00000181722 | ZBTB20 | -1.013939 | 4.00E-08 | 6.20E-07 |
| ENSG00000143382 | ADAMTSL4 | -1.020464 | 9.06E-07 | 1.05E-05 |
| ENSG00000099889 | ARVCF | -1.024453 | 0.012447337 | 0.044067208 |
| ENSG00000197261 | C6orf141 | -1.024533 | 0.004539874 | 0.019001288 |
| ENSG00000133134 | BEX2 | -1.027889 | 2.26E-05 | 0.000190165 |
| ENSG00000166192 | SENP8 | -1.028482 | 0.000248203 | 0.001611041 |
| ENSG00000013588 | GPRC5A | -1.034247 | 4.98E-33 | 7.95E-31 |
| ENSG00000179242 | CDH4 | -1.03708 | 8.33E-24 | 7.84E-22 |
| ENSG00000041515 | MYO16 | -1.039221 | 0.000174142 | 0.001166767 |
| ENSG00000129195 | PIMREG | -1.039362 | 0.005917559 | 0.02373583 |
| ENSG00000160207 | HSF2BP | -1.039528 | 0.000975606 | 0.005187908 |
| ENSG00000175556 | LONRF3 | -1.039864 | 4.84E-15 | 1.88E-13 |
| ENSG00000139132 | FGD4 | -1.041223 | 0.00312124 | 0.01397513 |
| ENSG00000065357 | DGKA | -1.045039 | 8.69E-05 | 0.000629375 |
| ENSG00000184792 | OSBP2 | -1.048682 | 1.84E-09 | 3.49E-08 |
| ENSG00000172731 | LRRC20 | -1.049018 | 1.64E-12 | 4.74E-11 |
| ENSG00000124772 | CPNE5 | -1.049188 | 0.000270896 | 0.001733416 |
| ENSG00000163909 | HEYL | -1.050626 | 0.002141754 | 0.010130003 |
| ENSG00000198919 | DZIP3 | -1.064269 | 7.76E-14 | 2.63E-12 |
| ENSG00000137962 | ARHGAP29 | -1.068899 | 6.86E-66 | 5.99E-63 |
| ENSG00000066279 | ASPM | -1.069278 | 2.15E-26 | 2.32E-24 |
| ENSG00000169760 | NLGN1 | -1.072863 | 0.00399839 | 0.01712923 |
| ENSG00000068489 | PRR11 | -1.073036 | 2.39E-20 | 1.67E-18 |
| ENSG00000177943 | MAMDC4 | -1.098032 | 0.000441453 | 0.002646295 |
| ENSG00000240445 | FOXO3B | -1.106828 | 1.71E-06 | 1.87E-05 |
| ENSG00000081181 | ARG2 | -1.119196 | 1.13E-11 | 2.89E-10 |
| ENSG00000078401 | EDN1 | -1.119793 | 1.78E-13 | 5.79E-12 |
| ENSG00000079385 | CEACAM1 | -1.126278 | 4.75E-07 | 5.92E-06 |
| ENSG00000112559 | MDFI | -1.1336 | 0.003519169 | 0.015476304 |
| ENSG00000115008 | IL1A | -1.133821 | 9.77E-54 | 4.41E-51 |
| ENSG00000135114 | OASL | -1.137504 | 0.00152131 | 0.007561497 |
| ENSG00000101096 | NFATC2 | -1.139565 | 3.44E-18 | 1.87E-16 |
| ENSG00000188177 | ZC3H6 | -1.140272 | 1.09E-07 | 1.55E-06 |
| ENSG00000110723 | EXPH5 | -1.140365 | 1.02E-18 | 5.87E-17 |
| ENSG00000156510 | HKDC1 | -1.144989 | 8.73E-41 | 2.00E-38 |
| ENSG00000129757 | CDKN1C | -1.149425 | 0.006888132 | 0.026935313 |
| ENSG00000083457 | ITGAE | -1.156869 | 0.000708247 | 0.003927915 |
| ENSG00000054392 | HHAT | -1.163009 | 3.31E-12 | 9.09E-11 |
| ENSG00000115163 | CENPA | -1.165234 | 0.000469226 | 0.002777172 |
| ENSG00000166851 | PLK1 | -1.165842 | 8.98E-15 | 3.37E-13 |
| ENSG00000122694 | GLIPR2 | -1.167932 | 1.48E-06 | 1.66E-05 |
| ENSG00000183778 | B3GALT5 | -1.173878 | 2.46E-15 | 9.87E-14 |
| ENSG00000138621 | PPCDC | -1.177076 | 1.59E-05 | 0.000139149 |
| ENSG00000164695 | CHMP4C | -1.184267 | 0.000582017 | 0.003317271 |
| ENSG00000183760 | ACP7 | -1.185069 | 0.000504599 | 0.002943944 |
| ENSG00000107864 | CPEB3 | -1.18548 | 9.90E-06 | 9.21E-05 |
| ENSG00000170961 | HAS2 | -1.186096 | 0.00858095 | 0.032412521 |
| ENSG00000100526 | CDKN3 | -1.187121 | 3.10E-09 | 5.75E-08 |
| ENSG00000117013 | KCNQ4 | -1.187627 | 0.006535662 | 0.025813947 |
| ENSG00000014914 | MTMR11 | -1.190345 | 3.52E-14 | 1.23E-12 |
| ENSG00000168811 | IL12A | -1.194362 | 7.90E-07 | 9.27E-06 |
| ENSG00000138356 | AOX1 | -1.19998 | 4.20E-45 | 1.22E-42 |
| ENSG00000111348 | ARHGDIB | -1.212255 | 5.68E-11 | 1.35E-09 |
| ENSG00000080031 | PTPRH | -1.212885 | 4.20E-07 | 5.30E-06 |
| ENSG00000186364 | NUDT17 | -1.213532 | 0.000108913 | 0.000772 |
| ENSG00000170175 | CHRNB1 | -1.214836 | 1.28E-35 | 2.37E-33 |
| ENSG00000131018 | SYNE1 | -1.221686 | 1.19E-19 | 7.59E-18 |
| ENSG00000137821 | LRRC49 | -1.223828 | 2.84E-13 | 9.05E-12 |
| ENSG00000117650 | NEK2 | -1.227138 | 6.14E-08 | 9.22E-07 |
| ENSG00000138772 | ANXA3 | -1.228228 | 1.27E-47 | 4.28E-45 |
| ENSG00000179148 | ALOXE3 | -1.22924 | 4.34E-16 | 1.92E-14 |
| ENSG00000115380 | EFEMP1 | -1.235982 | 4.30E-19 | 2.56E-17 |
| ENSG00000160712 | IL6R | -1.246794 | 2.39E-07 | 3.17E-06 |
| ENSG00000204262 | COL5A2 | -1.252387 | 0.00310703 | 0.013927866 |
| ENSG00000065618 | COL17A1 | -1.252542 | 1.89E-05 | 0.000162174 |
| ENSG00000188015 | S100A3 | -1.255639 | 0.000122269 | 0.000859664 |
| ENSG00000112782 | CLIC5 | -1.256014 | 0.000559603 | 0.003207673 |
| ENSG00000119771 | KLHL29 | -1.262261 | 5.41E-51 | 2.02E-48 |
| ENSG00000189410 | SH2D5 | -1.263034 | 1.51E-10 | 3.36E-09 |
| ENSG00000183696 | UPP1 | -1.270911 | 1.52E-90 | 2.85E-87 |
| ENSG00000221843 | C2orf16 | -1.2824 | 2.86E-05 | 0.000236295 |
| ENSG00000168685 | IL7R | -1.299189 | 6.87E-15 | 2.60E-13 |
| ENSG00000144583 | 4-Mar | -1.301133 | 1.47E-15 | 6.15E-14 |
| ENSG00000135063 | FAM189A2 | -1.304153 | 0.001109938 | 0.005764103 |
| ENSG00000168386 | FILIP1L | -1.30678 | 1.19E-20 | 8.54E-19 |
| ENSG00000134259 | NGF | -1.313301 | 0.000584642 | 0.003328146 |
| ENSG00000048740 | CELF2 | -1.316662 | 2.32E-21 | 1.82E-19 |
| ENSG00000012124 | CD22 | -1.332696 | 9.49E-54 | 4.41E-51 |
| ENSG00000162591 | MEGF6 | -1.334838 | 8.42E-11 | 1.96E-09 |
| ENSG00000183287 | CCBE1 | -1.334984 | 5.40E-05 | 0.00041608 |
| ENSG00000134198 | TSPAN2 | -1.339206 | 0.004919768 | 0.020305677 |
| ENSG00000167895 | TMC8 | -1.346698 | 2.72E-28 | 3.24E-26 |
| ENSG00000135604 | STX11 | -1.347923 | 0.006432568 | 0.025495776 |
| ENSG00000156869 | FRRS1 | -1.361419 | 8.11E-05 | 0.00059226 |
| ENSG00000170396 | ZNF804A | -1.36804 | 3.03E-08 | 4.80E-07 |
| ENSG00000116717 | GADD45A | -1.368914 | 3.33E-75 | 3.35E-72 |
| ENSG00000188910 | GJB3 | -1.369794 | 0.000250946 | 0.001625624 |
| ENSG00000205755 | CRLF2 | -1.371367 | 0.013716423 | 0.047683329 |
| ENSG00000164949 | GEM | -1.402994 | 1.55E-36 | 2.95E-34 |
| ENSG00000167617 | CDC42EP5 | -1.411779 | 0.003246462 | 0.014456695 |
| ENSG00000019186 | CYP24A1 | -1.429988 | 0.011816681 | 0.042153676 |
| ENSG00000164855 | TMEM184A | -1.443786 | 8.84E-13 | 2.65E-11 |
| ENSG00000184545 | DUSP8 | -1.451918 | 5.26E-14 | 1.80E-12 |
| ENSG00000158023 | WDR66 | -1.454888 | 1.10E-20 | 7.90E-19 |
| ENSG00000104177 | MYEF2 | -1.471036 | 1.96E-39 | 4.21E-37 |
| ENSG00000075340 | ADD2 | -1.472275 | 0.000435288 | 0.002615324 |
| ENSG00000139354 | GAS2L3 | -1.478145 | 7.65E-58 | 4.17E-55 |
| ENSG00000186517 | ARHGAP30 | -1.484643 | 6.16E-05 | 0.000464753 |
| ENSG00000137474 | MYO7A | -1.494197 | 9.59E-05 | 0.000688389 |
| ENSG00000138190 | EXOC6 | -1.496268 | 9.96E-07 | 1.14E-05 |
| ENSG00000226742 | HSBP1L1 | -1.505515 | 2.21E-05 | 0.000186601 |
| ENSG00000184185 | KCNJ12 | -1.512813 | 0.002387512 | 0.011131521 |
| ENSG00000078018 | MAP2 | -1.518729 | 0.000144008 | 0.000992813 |
| ENSG00000173212 | MAB21L3 | -1.540568 | 0.014109853 | 0.048817706 |
| ENSG00000144218 | AFF3 | -1.543142 | 5.02E-14 | 1.72E-12 |
| ENSG00000165046 | LETM2 | -1.549464 | 2.10E-17 | 1.07E-15 |
| ENSG00000027869 | SH2D2A | -1.55613 | 0.000195586 | 0.001299801 |
| ENSG00000143375 | CGN | -1.557482 | 0.000117908 | 0.000830365 |
| ENSG00000171877 | FRMD5 | -1.573253 | 3.61E-32 | 5.43E-30 |
| ENSG00000169429 | CXCL8 | -1.583813 | 3.67E-19 | 2.22E-17 |
| ENSG00000166432 | ZMAT1 | -1.606231 | 9.87E-09 | 1.68E-07 |
| ENSG00000135549 | PKIB | -1.611228 | 7.95E-06 | 7.53E-05 |
| ENSG00000127325 | BEST3 | -1.61168 | 0.000115577 | 0.000816145 |
| ENSG00000198673 | TAFA2 | -1.613914 | 1.34E-05 | 0.000120322 |
| ENSG00000149418 | ST14 | -1.619728 | 0.000259893 | 0.001672507 |
| ENSG00000123610 | TNFAIP6 | -1.65355 | 0.00021371 | 0.001412366 |
| ENSG00000145113 | MUC4 | -1.666757 | 4.85E-10 | 9.96E-09 |
| ENSG00000049192 | ADAMTS6 | -1.685566 | 1.45E-45 | 4.42E-43 |
| ENSG00000147168 | IL2RG | -1.689246 | 0.000298325 | 0.001886143 |
| ENSG00000117069 | ST6GALNAC5 | -1.693897 | 4.91E-07 | 6.08E-06 |
| ENSG00000132561 | MATN2 | -1.699963 | 1.66E-09 | 3.16E-08 |
| ENSG00000104413 | ESRP1 | -1.707506 | 1.01E-06 | 1.15E-05 |
| ENSG00000149573 | MPZL2 | -1.726239 | 0.000530128 | 0.003062855 |
| ENSG00000079931 | MOXD1 | -1.744743 | 0.000224818 | 0.00147757 |
| ENSG00000138769 | CDKL2 | -1.756729 | 1.34E-05 | 0.00012018 |
| ENSG00000187800 | PEAR1 | -1.758403 | 2.05E-10 | 4.47E-09 |
| ENSG00000163661 | PTX3 | -1.758788 | 2.71E-105 | 5.92E-102 |
| ENSG00000172602 | RND1 | -1.760617 | 0.000136237 | 0.00094521 |
| ENSG00000284906 | ARHGAP11B | -1.763085 | 0.000166283 | 0.001120999 |
| ENSG00000170381 | SEMA3E | -1.78617 | 0.010770848 | 0.039039851 |
| ENSG00000140030 | GPR65 | -1.788496 | 0.011373582 | 0.040891054 |
| ENSG00000081041 | CXCL2 | -1.791774 | 2.31E-61 | 1.60E-58 |
| ENSG00000133083 | DCLK1 | -1.796128 | 1.92E-08 | 3.12E-07 |
| ENSG00000075213 | SEMA3A | -1.796655 | 2.67E-05 | 0.00022177 |
| ENSG00000116701 | NCF2 | -1.802319 | 2.08E-08 | 3.36E-07 |
| ENSG00000187688 | TRPV2 | -1.81274 | 8.49E-08 | 1.23E-06 |
| ENSG00000182901 | RGS7 | -1.839776 | 5.30E-05 | 0.000408785 |
| ENSG00000197415 | VEPH1 | -1.907702 | 2.73E-11 | 6.66E-10 |
| ENSG00000144063 | MALL | -1.912127 | 0.001179033 | 0.006081917 |
| ENSG00000101333 | PLCB4 | -1.91427 | 0.000452492 | 0.002696462 |
| ENSG00000135253 | KCP | -1.916477 | 2.12E-18 | 1.17E-16 |
| ENSG00000149591 | TAGLN | -1.946099 | 6.45E-15 | 2.46E-13 |
| ENSG00000113578 | FGF1 | -1.986579 | 6.43E-06 | 6.24E-05 |
| ENSG00000176406 | RIMS2 | -2.012881 | 2.41E-15 | 9.73E-14 |
| ENSG00000169085 | VXN | -2.037868 | 1.17E-05 | 0.000106358 |
| ENSG00000243137 | PSG4 | -2.083097 | 1.09E-27 | 1.25E-25 |
| ENSG00000148483 | TMEM236 | -2.10343 | 1.01E-05 | 9.37E-05 |
| ENSG00000163347 | CLDN1 | -2.134531 | 1.07E-87 | 1.56E-84 |
| ENSG00000181634 | TNFSF15 | -2.250418 | 5.25E-158 | 1.72E-154 |
| ENSG00000170498 | KISS1 | -2.276918 | 2.38E-07 | 3.15E-06 |
| ENSG00000170373 | CST1 | -2.296982 | 0.006695968 | 0.026333315 |
| ENSG00000133101 | CCNA1 | -2.30592 | 8.10E-08 | 1.18E-06 |
| ENSG00000111700 | SLCO1B3 | -2.316231 | 1.19E-16 | 5.58E-15 |
| ENSG00000189056 | RELN | -2.376721 | 1.59E-05 | 0.000138777 |
| ENSG00000172967 | XKR3 | -2.400538 | 0.008650606 | 0.03260977 |
| ENSG00000138161 | CUZD1 | -2.434937 | 0.007182429 | 0.027869698 |
| ENSG00000131242 | RAB11FIP4 | -2.436099 | 0.001620823 | 0.00799541 |
| ENSG00000167306 | MYO5B | -2.584963 | 1.63E-06 | 1.80E-05 |
| ENSG00000151702 | FLI1 | -2.588298 | 2.89E-08 | 4.61E-07 |
| ENSG00000165071 | TMEM71 | -2.644625 | 1.66E-31 | 2.42E-29 |
| ENSG00000249751 | ECSCR | -2.690316 | 0.003729184 | 0.016225481 |
| ENSG00000170323 | FABP4 | -2.713696 | 0.002313527 | 0.010821254 |
| ENSG00000198074 | AKR1B10 | -2.743902 | 0.008163037 | 0.031030917 |
| ENSG00000163121 | NEURL3 | -2.974615 | 6.15E-09 | 1.08E-07 |
| ENSG00000047457 | CP | -3.017074 | 8.85E-06 | 8.31E-05 |
| ENSG00000169994 | MYO7B | -3.087463 | 2.29E-09 | 4.28E-08 |
| ENSG00000196188 | CTSE | -3.566347 | 0.000228003 | 0.001496248 |
| ENSG00000093134 | VNN3 | -3.616259 | 1.12E-11 | 2.88E-10 |
| ENSG00000100867 | DHRS2 | -4.146841 | 3.00E-07 | 3.90E-06 |
| ENSG00000151967 | SCHIP1 | -4.203872 | 2.44E-05 | 0.000204143 |
| ENSG00000205670 | SMIM11A | -4.781804 | 0.006819644 | 0.026715373 |
| ENSG00000215182 | MUC5AC | -5.066089 | 1.39E-08 | 2.31E-07 |
| ENSG00000117983 | MUC5B | -7.544321 | 0.012712263 | 0.044772889 |
| ENSG00000086548 | CEACAM6 | -7.697663 | 1.03E-09 | 2.03E-08 |
