# Supplementary material for: FDI-6 inhibits the expression and function of FOXM1 to sensitize BRCA-proficient triple-negative breast cancer cells to Olaparib by regulating cell cycle progression and DNA damage repair
Source: Cell Death Dis. 2021 Dec 8;12(12):1138. doi: 10.1038/s41419-021-04434-9 (PMC8654856; doi:10.1038/s41419-021-04434-9)
Supplement: Supplementary file 21 — Supplementary Table 5 [file 41419_2021_4434_MOESM21_ESM.doc]

**Supplemental Tables**

**Supplemental Table 5. DEGs in control vs Olaparib treated group analyzed by RNA sequencing.**

| ID | Symbol | log_2_(fc) | P Value | FDR |
| --- | --- | --- | --- | --- |
| ENSG00000162892 | IL24 | -1.298333 | 5.05E-85 | 6.34E-81 |
| ENSG00000119922 | IFIT2 | 1.5845192 | 1.66E-66 | 5.21E-63 |
| ENSG00000185745 | IFIT1 | 1.9751315 | 6.18E-36 | 5.54E-33 |
| ENSG00000173530 | TNFRSF10D | 1.2759206 | 3.89E-30 | 1.88E-27 |
| ENSG00000164283 | ESM1 | 1.9144222 | 1.24E-27 | 5.18E-25 |
| ENSG00000184992 | BRI3BP | 1.2776296 | 1.85E-27 | 7.25E-25 |
| ENSG00000175305 | CCNE2 | 1.6797298 | 8.56E-26 | 2.99E-23 |
| ENSG00000092853 | CLSPN | 1.2256041 | 3.02E-20 | 6.54E-18 |
| ENSG00000183778 | B3GALT5 | -1.275323 | 8.47E-16 | 9.41E-14 |
| ENSG00000066056 | TIE1 | 1.0698281 | 3.03E-15 | 3.07E-13 |
| ENSG00000187608 | ISG15 | 1.1596783 | 4.21E-14 | 3.62E-12 |
| ENSG00000189057 | FAM111B | 1.7411534 | 1.03E-13 | 8.42E-12 |
| ENSG00000133119 | RFC3 | 1.0182742 | 2.07E-12 | 1.29E-10 |
| ENSG00000258465 | AL139011.2 | 1.5275043 | 6.28E-11 | 2.89E-09 |
| ENSG00000132561 | MATN2 | 1.1688658 | 1.72E-10 | 7.06E-09 |
| ENSG00000286132 | AC022415.2 | 1.0218831 | 2.15E-10 | 8.72E-09 |
| ENSG00000171320 | ESCO2 | 1.6523707 | 2.29E-10 | 9.19E-09 |
| ENSG00000135114 | OASL | 1.4112492 | 2.74E-10 | 1.08E-08 |
| ENSG00000137965 | IFI44 | 1.1036356 | 3.47E-10 | 1.32E-08 |
| ENSG00000130487 | KLHDC7B | -1.699295 | 6.76E-10 | 2.45E-08 |
| ENSG00000120738 | EGR1 | -1.054079 | 8.54E-09 | 2.43E-07 |
| ENSG00000241322 | CDRT1 | -1.208674 | 2.04E-08 | 5.30E-07 |
| ENSG00000136982 | DSCC1 | 1.1331251 | 1.02E-07 | 2.19E-06 |
| ENSG00000137628 | DDX60 | 1.042178 | 1.52E-07 | 3.06E-06 |
| ENSG00000267041 | ZNF850 | 1.140725 | 2.78E-07 | 5.36E-06 |
| ENSG00000204287 | HLA-DRA | -1.03827 | 3.76E-07 | 6.93E-06 |
| ENSG00000124788 | ATXN1 | -1.246316 | 5.11E-07 | 9.08E-06 |
| ENSG00000150281 | CTF1 | -1.06805 | 6.53E-07 | 1.13E-05 |
| ENSG00000093134 | VNN3 | -2.039758 | 1.07E-06 | 1.76E-05 |
| ENSG00000205923 | CEMP1 | 6.3750394 | 1.17E-06 | 1.89E-05 |
| ENSG00000162894 | FCMR | -1.364498 | 1.65E-06 | 2.60E-05 |
| ENSG00000147614 | ATP6V0D2 | 1.1119386 | 2.38E-06 | 3.58E-05 |
| ENSG00000198056 | PRIM1 | 1.1787743 | 3.62E-06 | 5.14E-05 |
| ENSG00000152147 | GEMIN6 | 1.1572456 | 4.18E-06 | 5.78E-05 |
| ENSG00000181544 | FANCB | 1.2142069 | 4.52E-06 | 6.18E-05 |
| ENSG00000011052 | NME1-NME2 | 1.3136841 | 4.79E-06 | 6.47E-05 |
| ENSG00000154175 | ABI3BP | 1.073114 | 7.93E-06 | 0.0001 |
| ENSG00000251537 | AC005324.3 | -1.169925 | 1.79E-05 | 0.000201 |
| ENSG00000145569 | OTULINL | 1.0280144 | 2.68E-05 | 0.000285 |
| ENSG00000105290 | APLP1 | 1.255915 | 4.14E-05 | 0.000416 |
| ENSG00000284969 | AL049629.2 | 1.9514915 | 4.56E-05 | 0.000452 |
| ENSG00000081853 | PCDHGA2 | -1.532733 | 5.18E-05 | 0.000507 |
| ENSG00000095739 | BAMBI | 1.0035145 | 0.0001086 | 0.000969 |
| ENSG00000186642 | PDE2A | -1.050878 | 0.0001383 | 0.001188 |
| ENSG00000064489 | BORCS8-MEF2B | -1.174586 | 0.0001826 | 0.001496 |
| ENSG00000111319 | SCNN1A | -1.079387 | 0.0001907 | 0.001553 |
| ENSG00000171931 | FBXW10 | -1.005901 | 0.0002231 | 0.001774 |
| ENSG00000162654 | GBP4 | 1.0665977 | 0.0002664 | 0.002055 |
| ENSG00000164692 | COL1A2 | -7.67948 | 0.0002841 | 0.002169 |
| ENSG00000188015 | S100A3 | -1.045676 | 0.0003348 | 0.002485 |
| ENSG00000171443 | ZNF524 | -1.15624 | 0.0003899 | 0.002826 |
| ENSG00000085840 | ORC1 | 1.0780025 | 0.0004764 | 0.003317 |
| ENSG00000007968 | E2F2 | 1.5643522 | 0.0007143 | 0.004639 |
| ENSG00000068615 | REEP1 | 1.91908 | 0.0007626 | 0.004884 |
| ENSG00000123570 | RAB9B | 1.0551416 | 0.0011716 | 0.006993 |
| ENSG00000168542 | COL3A1 | -4.675565 | 0.0012884 | 0.007568 |
| ENSG00000258677 | AC022826.2 | -1.281487 | 0.0014094 | 0.008191 |
| ENSG00000183128 | CALHM3 | -1.5807 | 0.0017821 | 0.009869 |
| ENSG00000115738 | ID2 | 1.7064803 | 0.0018898 | 0.010292 |
| ENSG00000170962 | PDGFD | 1.09586 | 0.0024116 | 0.012578 |
| ENSG00000180616 | SSTR2 | 1.2129937 | 0.0025367 | 0.013127 |
| ENSG00000215182 | MUC5AC | -4.481127 | 0.0030507 | 0.01521 |
| ENSG00000186517 | ARHGAP30 | -1.144184 | 0.0032159 | 0.015863 |
| ENSG00000284906 | ARHGAP11B | -1.206137 | 0.0033047 | 0.016181 |
| ENSG00000129757 | CDKN1C | -1.115477 | 0.0041379 | 0.019559 |
| ENSG00000136237 | RAPGEF5 | 1.8259706 | 0.0041846 | 0.019737 |
| ENSG00000166396 | SERPINB7 | 1.2137793 | 0.0044012 | 0.020564 |
| ENSG00000170442 | KRT86 | -1.239188 | 0.0044729 | 0.020775 |
| ENSG00000147676 | MAL2 | 1.9891932 | 0.0045488 | 0.021026 |
| ENSG00000086548 | CEACAM6 | -8.434628 | 0.0046454 | 0.021379 |
| ENSG00000196456 | ZNF775 | -1.106915 | 0.0046735 | 0.021484 |
| ENSG00000064687 | ABCA7 | 1.032193 | 0.0047275 | 0.021661 |
| ENSG00000135750 | KCNK1 | 1.002871 | 0.0057001 | 0.025074 |
| ENSG00000169851 | PCDH7 | 1.1255309 | 0.0057489 | 0.025245 |
| ENSG00000235109 | ZSCAN31 | -1.106073 | 0.0058758 | 0.02573 |
| ENSG00000242221 | PSG2 | 1.1214314 | 0.0066251 | 0.028432 |
| ENSG00000179841 | AKAP5 | 1.6374299 | 0.0076955 | 0.031956 |
| ENSG00000079393 | DUSP13 | 1.284096 | 0.0077862 | 0.032232 |
| ENSG00000174721 | FGFBP3 | -1.060696 | 0.0089751 | 0.035932 |
| ENSG00000197506 | SLC28A3 | 1.16711 | 0.0096557 | 0.038079 |
| ENSG00000243708 | PLA2G4B | -1.040957 | 0.0096756 | 0.03814 |
| ENSG00000080493 | SLC4A4 | 1.3785116 | 0.010528 | 0.040646 |
| ENSG00000105388 | CEACAM5 | -6.558421 | 0.0107092 | 0.041257 |
| ENSG00000169684 | CHRNA5 | 1.2090513 | 0.0115277 | 0.043779 |
| ENSG00000269313 | MAGIX | -1.517848 | 0.0117164 | 0.044321 |
| ENSG00000170469 | SPATA24 | -1.231243 | 0.0122534 | 0.046023 |
| ENSG00000038427 | VCAN | -1.179324 | 0.0130112 | 0.048317 |
| ENSG00000170122 | FOXD4 | -1.36257 | 0.0132473 | 0.049049 |
| ENSG00000214967 | NPIPA7 | -1.165115 | 0.0133851 | 0.049442 |
| ENSG00000275074 | NUDT18 | -1.300233 | 0.0135446 | 0.049782 |
