# Supplementary material for: FDI-6 inhibits the expression and function of FOXM1 to sensitize BRCA-proficient triple-negative breast cancer cells to Olaparib by regulating cell cycle progression and DNA damage repair
Source: Cell Death Dis. 2021 Dec 8;12(12):1138. doi: 10.1038/s41419-021-04434-9 (PMC8654856; doi:10.1038/s41419-021-04434-9)
Supplement: Supplementary file 22 — Supplementary Table 6 [file 41419_2021_4434_MOESM22_ESM.doc]

**Supplemental Tables**

**Supplemental Table 6. DEGs in control vs FDI-6/Olaparib co-treated group analyzed by RNA sequencing.**

| ID | Symbol | log_2_(fc) | P Value | FDR |
| --- | --- | --- | --- | --- |
| ENSG00000138061 | CYP1B1 | 1.964662 | 0 | 0 |
| ENSG00000149968 | MMP3 | 2.338077 | 0 | 0 |
| ENSG00000196611 | MMP1 | 3.670781 | 5.06E-264 | 2.33E-260 |
| ENSG00000109971 | HSPA8 | 1.320681 | 8.04E-257 | 2.77E-253 |
| ENSG00000197632 | SERPINB2 | 3.716255 | 1.21E-256 | 3.34E-253 |
| ENSG00000163661 | PTX3 | -1.75586 | 9.97E-216 | 2.29E-212 |
| ENSG00000213949 | ITGA1 | 1.659922 | 1.78E-187 | 3.52E-184 |
| ENSG00000140465 | CYP1A1 | 3.032828 | 8.66E-176 | 1.49E-172 |
| ENSG00000106366 | SERPINE1 | 1.003481 | 9.98E-172 | 1.53E-168 |
| ENSG00000163659 | TIPARP | 1.398939 | 1.04E-146 | 1.43E-143 |
| ENSG00000125538 | IL1B | 1.151807 | 3.01E-135 | 3.78E-132 |
| ENSG00000041982 | TNC | -1.97081 | 1.33E-133 | 1.53E-130 |
| ENSG00000157227 | MMP14 | 1.15329 | 3.72E-114 | 3.66E-111 |
| ENSG00000137801 | THBS1 | 1.236189 | 1.53E-108 | 1.41E-105 |
| ENSG00000130513 | GDF15 | -1.12092 | 1.36E-104 | 1.17E-101 |
| ENSG00000104738 | MCM4 | 1.29192 | 2.82E-100 | 2.29E-97 |
| ENSG00000101255 | TRIB3 | -1.17954 | 2.95E-92 | 2.27E-89 |
| ENSG00000172432 | GTPBP2 | -1.07394 | 6.61E-89 | 4.80E-86 |
| ENSG00000184254 | ALDH1A3 | 1.771466 | 1.05E-87 | 7.25E-85 |
| ENSG00000163347 | CLDN1 | -1.79765 | 2.36E-82 | 1.55E-79 |
| ENSG00000171848 | RRM2 | 1.595653 | 1.28E-73 | 7.66E-71 |
| ENSG00000111799 | COL12A1 | -1.0041 | 3.27E-71 | 1.88E-68 |
| ENSG00000112118 | MCM3 | 1.141438 | 1.73E-68 | 9.57E-66 |
| ENSG00000163638 | ADAMTS9 | 1.259622 | 1.20E-67 | 6.39E-65 |
| ENSG00000189060 | H1F0 | -1.00196 | 4.70E-66 | 2.24E-63 |
| ENSG00000108846 | ABCC3 | -1.32749 | 1.49E-61 | 6.41E-59 |
| ENSG00000131016 | AKAP12 | 1.805476 | 2.09E-61 | 8.72E-59 |
| ENSG00000163131 | CTSS | 1.005583 | 3.90E-57 | 1.54E-54 |
| ENSG00000089723 | OTUB2 | 1.471463 | 1.73E-56 | 6.43E-54 |
| ENSG00000072310 | SREBF1 | -1.01407 | 3.60E-52 | 1.18E-49 |
| ENSG00000105825 | TFPI2 | 1.046346 | 4.14E-51 | 1.29E-48 |
| ENSG00000167767 | KRT80 | -1.05798 | 5.03E-51 | 1.51E-48 |
| ENSG00000119771 | KLHL29 | -1.13237 | 2.35E-50 | 6.90E-48 |
| ENSG00000092853 | CLSPN | 1.687047 | 2.64E-48 | 7.01E-46 |
| ENSG00000144810 | COL8A1 | -1.11599 | 3.96E-48 | 1.03E-45 |
| ENSG00000132510 | KDM6B | -1.33982 | 5.98E-48 | 1.53E-45 |
| ENSG00000134369 | NAV1 | -1.04311 | 4.22E-44 | 9.54E-42 |
| ENSG00000128283 | CDC42EP1 | -1.11332 | 8.67E-44 | 1.90E-41 |
| ENSG00000132646 | PCNA | 1.175768 | 1.01E-43 | 2.18E-41 |
| ENSG00000143476 | DTL | 1.318002 | 1.90E-43 | 4.03E-41 |
| ENSG00000108239 | TBC1D12 | 1.108362 | 5.43E-43 | 1.12E-40 |
| ENSG00000175305 | CCNE2 | 2.152761 | 7.50E-43 | 1.52E-40 |
| ENSG00000114812 | VIPR1 | 1.582077 | 9.64E-43 | 1.93E-40 |
| ENSG00000276043 | UHRF1 | 1.163744 | 2.08E-42 | 4.11E-40 |
| ENSG00000139354 | GAS2L3 | -1.37513 | 2.57E-42 | 5.00E-40 |
| ENSG00000076003 | MCM6 | 1.078602 | 1.14E-40 | 2.10E-38 |
| ENSG00000180730 | SHISA2 | 1.851685 | 5.37E-39 | 9.15E-37 |
| ENSG00000173530 | TNFRSF10D | 1.397823 | 3.50E-38 | 5.74E-36 |
| ENSG00000121858 | TNFSF10 | 2.079126 | 5.88E-38 | 9.55E-36 |
| ENSG00000119714 | GPR68 | 1.372566 | 5.84E-37 | 8.96E-35 |
| ENSG00000180573 | HIST1H2AC | 1.209051 | 7.17E-37 | 1.08E-34 |
| ENSG00000058335 | RASGRF1 | -1.24397 | 1.05E-36 | 1.54E-34 |
| ENSG00000179242 | CDH4 | -1.25345 | 2.54E-36 | 3.62E-34 |
| ENSG00000095002 | MSH2 | 1.004318 | 8.96E-36 | 1.21E-33 |
| ENSG00000164949 | GEM | -1.18662 | 5.25E-34 | 6.41E-32 |
| ENSG00000119922 | IFIT2 | 1.231953 | 8.61E-34 | 1.03E-31 |
| ENSG00000179532 | DNHD1 | -1.06277 | 1.39E-33 | 1.66E-31 |
| ENSG00000184992 | BRI3BP | 1.368616 | 1.33E-32 | 1.52E-30 |
| ENSG00000094804 | CDC6 | 1.027286 | 4.43E-32 | 4.93E-30 |
| ENSG00000011201 | ANOS1 | 1.184139 | 7.18E-32 | 7.93E-30 |
| ENSG00000164283 | ESM1 | 1.982758 | 7.62E-32 | 8.35E-30 |
| ENSG00000110031 | LPXN | 1.408567 | 5.31E-31 | 5.64E-29 |
| ENSG00000167797 | CDK2AP2 | -1.02806 | 1.16E-30 | 1.21E-28 |
| ENSG00000147614 | ATP6V0D2 | 2.287208 | 1.18E-30 | 1.21E-28 |
| ENSG00000065328 | MCM10 | 1.2997 | 2.10E-29 | 1.96E-27 |
| ENSG00000189057 | FAM111B | 2.204912 | 2.88E-29 | 2.63E-27 |
| ENSG00000179046 | TRIML2 | -1.31509 | 5.43E-29 | 4.86E-27 |
| ENSG00000101412 | E2F1 | 1.312768 | 1.30E-27 | 1.12E-25 |
| ENSG00000164251 | F2RL1 | 1.386322 | 1.40E-27 | 1.20E-25 |
| ENSG00000165071 | TMEM71 | -2.10715 | 1.64E-27 | 1.40E-25 |
| ENSG00000146678 | IGFBP1 | 1.195682 | 1.73E-26 | 1.35E-24 |
| ENSG00000171992 | SYNPO | -1.04066 | 6.16E-26 | 4.78E-24 |
| ENSG00000132846 | ZBED3 | -1.08104 | 8.76E-26 | 6.64E-24 |
| ENSG00000169129 | AFAP1L2 | 1.379414 | 1.39E-25 | 1.05E-23 |
| ENSG00000197299 | BLM | 1.269914 | 3.62E-25 | 2.67E-23 |
| ENSG00000167895 | TMC8 | -1.11524 | 1.21E-24 | 8.63E-23 |
| ENSG00000154310 | TNIK | 1.298209 | 1.84E-24 | 1.30E-22 |
| ENSG00000106948 | AKNA | -1.13781 | 2.76E-24 | 1.89E-22 |
| ENSG00000172086 | KRCC1 | -1.02549 | 3.34E-24 | 2.27E-22 |
| ENSG00000132613 | MTSS2 | -1.00122 | 3.44E-24 | 2.33E-22 |
| ENSG00000185745 | IFIT1 | 1.574594 | 1.50E-23 | 9.53E-22 |
| ENSG00000102312 | PORCN | 1.093421 | 1.02E-22 | 6.24E-21 |
| ENSG00000180884 | ZNF792 | 1.658471 | 1.68E-21 | 9.63E-20 |
| ENSG00000182752 | PAPPA | 1.01719 | 2.13E-21 | 1.20E-19 |
| ENSG00000197635 | DPP4 | 1.548825 | 3.20E-21 | 1.79E-19 |
| ENSG00000133119 | RFC3 | 1.227457 | 6.97E-21 | 3.80E-19 |
| ENSG00000130487 | KLHDC7B | -3.07039 | 7.06E-20 | 3.46E-18 |
| ENSG00000164045 | CDC25A | 1.065479 | 2.16E-19 | 1.02E-17 |
| ENSG00000198056 | PRIM1 | 1.895252 | 3.59E-19 | 1.68E-17 |
| ENSG00000171877 | FRMD5 | -1.30031 | 5.34E-19 | 2.47E-17 |
| ENSG00000112297 | CRYBG1 | -1.22038 | 2.12E-18 | 9.27E-17 |
| ENSG00000187608 | ISG15 | 1.256634 | 3.19E-18 | 1.38E-16 |
| ENSG00000136982 | DSCC1 | 1.659163 | 7.61E-18 | 3.18E-16 |
| ENSG00000108515 | ENO3 | -1.13327 | 1.22E-17 | 5.04E-16 |
| ENSG00000158373 | HIST1H2BD | 1.319359 | 2.42E-17 | 9.60E-16 |
| ENSG00000168386 | FILIP1L | -1.10284 | 2.47E-17 | 9.76E-16 |
| ENSG00000177602 | HASPIN | 1.232204 | 2.48E-17 | 9.80E-16 |
| ENSG00000100297 | MCM5 | 1.011799 | 2.52E-17 | 9.90E-16 |
| ENSG00000165244 | ZNF367 | 1.06815 | 6.76E-17 | 2.58E-15 |
| ENSG00000092470 | WDR76 | 1.612539 | 8.44E-17 | 3.19E-15 |
| ENSG00000178764 | ZHX2 | -1.05637 | 1.13E-16 | 4.20E-15 |
| ENSG00000159147 | DONSON | 1.012215 | 1.49E-16 | 5.50E-15 |
| ENSG00000267041 | ZNF850 | 1.44575 | 1.62E-16 | 5.99E-15 |
| ENSG00000135253 | KCP | -1.82562 | 3.84E-16 | 1.36E-14 |
| ENSG00000144681 | STAC | -1.23887 | 6.11E-16 | 2.12E-14 |
| ENSG00000274559 | CU639417.1 | 1.303839 | 7.98E-16 | 2.73E-14 |
| ENSG00000154734 | ADAMTS1 | 1.180236 | 8.80E-16 | 2.98E-14 |
| ENSG00000165046 | LETM2 | -1.56984 | 1.34E-15 | 4.45E-14 |
| ENSG00000258465 | AL139011.2 | 1.818124 | 1.46E-15 | 4.83E-14 |
| ENSG00000265190 | ANXA8 | 1.419803 | 1.47E-15 | 4.84E-14 |
| ENSG00000144354 | CDCA7 | 1.183646 | 1.51E-15 | 4.95E-14 |
| ENSG00000108932 | SLC16A6 | 1.876397 | 3.28E-15 | 1.04E-13 |
| ENSG00000136492 | BRIP1 | 1.177787 | 3.62E-15 | 1.15E-13 |
| ENSG00000221869 | CEBPD | -1.10751 | 7.64E-15 | 2.33E-13 |
| ENSG00000099998 | GGT5 | -1.70083 | 1.44E-14 | 4.28E-13 |
| ENSG00000196584 | XRCC2 | 1.130483 | 2.62E-14 | 7.43E-13 |
| ENSG00000174371 | EXO1 | 1.096845 | 6.48E-14 | 1.75E-12 |
| ENSG00000259207 | ITGB3 | 1.100973 | 8.23E-14 | 2.19E-12 |
| ENSG00000169258 | GPRIN1 | 1.371598 | 1.00E-13 | 2.64E-12 |
| ENSG00000049769 | PPP1R3F | -1.13548 | 1.00E-13 | 2.64E-12 |
| ENSG00000286132 | AC022415.2 | 1.145215 | 1.19E-13 | 3.07E-12 |
| ENSG00000241322 | CDRT1 | -1.39289 | 1.60E-13 | 4.08E-12 |
| ENSG00000167772 | ANGPTL4 | 2.036837 | 1.97E-13 | 4.94E-12 |
| ENSG00000164855 | TMEM184A | -1.54507 | 2.19E-13 | 5.44E-12 |
| ENSG00000171320 | ESCO2 | 1.640661 | 2.73E-13 | 6.76E-12 |
| ENSG00000183778 | B3GALT5 | -1.33382 | 4.41E-13 | 1.06E-11 |
| ENSG00000158483 | FAM86C1 | -1.04824 | 5.99E-13 | 1.43E-11 |
| ENSG00000102996 | MMP15 | 1.014002 | 1.24E-12 | 2.85E-11 |
| ENSG00000181722 | ZBTB20 | -1.21662 | 1.49E-12 | 3.38E-11 |
| ENSG00000131018 | SYNE1 | -1.1123 | 1.75E-12 | 3.93E-11 |
| ENSG00000100479 | POLE2 | 1.36062 | 7.11E-12 | 1.48E-10 |
| ENSG00000137752 | CASP1 | 1.172213 | 8.50E-12 | 1.74E-10 |
| ENSG00000164379 | FOXQ1 | 1.944949 | 1.08E-11 | 2.18E-10 |
| ENSG00000184792 | OSBP2 | -1.13305 | 1.14E-11 | 2.28E-10 |
| ENSG00000137821 | LRRC49 | -1.01514 | 1.46E-11 | 2.88E-10 |
| ENSG00000104413 | ESRP1 | -2.11235 | 1.63E-11 | 3.20E-10 |
| ENSG00000144554 | FANCD2 | 1.027013 | 1.93E-11 | 3.79E-10 |
| ENSG00000101901 | ALG13 | -1.27124 | 2.17E-11 | 4.23E-10 |
| ENSG00000242419 | PCDHGC4 | -2.98453 | 3.03E-11 | 5.78E-10 |
| ENSG00000078081 | LAMP3 | -1.28021 | 5.25E-11 | 9.72E-10 |
| ENSG00000081181 | ARG2 | -1.07262 | 5.36E-11 | 9.92E-10 |
| ENSG00000204406 | MBD5 | -1.15321 | 5.52E-11 | 1.02E-09 |
| ENSG00000101003 | GINS1 | 1.207452 | 5.75E-11 | 1.06E-09 |
| ENSG00000186523 | FAM86B1 | -1.25308 | 7.72E-11 | 1.39E-09 |
| ENSG00000138346 | DNA2 | 1.13236 | 1.17E-10 | 2.06E-09 |
| ENSG00000132326 | PER2 | -1.18386 | 1.58E-10 | 2.71E-09 |
| ENSG00000166801 | FAM111A | 1.049374 | 1.67E-10 | 2.86E-09 |
| ENSG00000166396 | SERPINB7 | 2.210897 | 2.29E-10 | 3.83E-09 |
| ENSG00000184545 | DUSP8 | -1.11631 | 2.78E-10 | 4.57E-09 |
| ENSG00000157110 | RBPMS | 1.013772 | 3.72E-10 | 6.04E-09 |
| ENSG00000184271 | POU6F1 | -1.8005 | 4.31E-10 | 6.92E-09 |
| ENSG00000186871 | ERCC6L | 1.146357 | 7.27E-10 | 1.12E-08 |
| ENSG00000143603 | KCNN3 | 1.224966 | 1.02E-09 | 1.53E-08 |
| ENSG00000167513 | CDT1 | 1.043873 | 1.28E-09 | 1.90E-08 |
| ENSG00000167191 | GPRC5B | 1.159819 | 1.43E-09 | 2.11E-08 |
| ENSG00000168936 | TMEM129 | -1.05802 | 1.55E-09 | 2.27E-08 |
| ENSG00000132470 | ITGB4 | -1.12037 | 2.30E-09 | 3.29E-08 |
| ENSG00000187688 | TRPV2 | -1.97232 | 2.74E-09 | 3.85E-08 |
| ENSG00000166922 | SCG5 | 1.571526 | 2.99E-09 | 4.18E-08 |
| ENSG00000180596 | HIST1H2BC | 1.529992 | 3.88E-09 | 5.32E-08 |
| ENSG00000175287 | PHYHD1 | -1.12127 | 4.16E-09 | 5.69E-08 |
| ENSG00000170054 | SERPINA9 | 5.095924 | 4.76E-09 | 6.48E-08 |
| ENSG00000124788 | ATXN1 | -1.41695 | 6.10E-09 | 8.19E-08 |
| ENSG00000181544 | FANCB | 1.499977 | 7.99E-09 | 1.06E-07 |
| ENSG00000103888 | CEMIP | 2.470027 | 9.68E-09 | 1.26E-07 |
| ENSG00000131153 | GINS2 | 1.43958 | 1.35E-08 | 1.72E-07 |
| ENSG00000105290 | APLP1 | 1.736966 | 1.36E-08 | 1.73E-07 |
| ENSG00000182378 | PLCXD1 | 1.504706 | 1.60E-08 | 1.99E-07 |
| ENSG00000170915 | PAQR8 | -1.7632 | 1.62E-08 | 2.02E-07 |
| ENSG00000085840 | ORC1 | 1.557995 | 2.07E-08 | 2.54E-07 |
| ENSG00000188177 | ZC3H6 | -1.05161 | 2.40E-08 | 2.90E-07 |
| ENSG00000138395 | CDK15 | 2.825971 | 2.48E-08 | 3.00E-07 |
| ENSG00000114491 | UMPS | 1.223948 | 3.58E-08 | 4.20E-07 |
| ENSG00000093134 | VNN3 | -2.16375 | 4.16E-08 | 4.81E-07 |
| ENSG00000116675 | DNAJC6 | -1.01455 | 5.03E-08 | 5.69E-07 |
| ENSG00000129173 | E2F8 | 1.195551 | 5.62E-08 | 6.30E-07 |
| ENSG00000188015 | S100A3 | -1.93597 | 6.82E-08 | 7.54E-07 |
| ENSG00000147804 | SLC39A4 | -1.08875 | 8.69E-08 | 9.43E-07 |
| ENSG00000051180 | RAD51 | 1.012318 | 8.70E-08 | 9.43E-07 |
| ENSG00000123096 | SSPN | -2.08549 | 9.57E-08 | 1.03E-06 |
| ENSG00000197415 | VEPH1 | -1.10296 | 1.28E-07 | 1.34E-06 |
| ENSG00000167889 | MGAT5B | -1.04803 | 1.57E-07 | 1.62E-06 |
| ENSG00000128510 | CPA4 | 1.009438 | 1.83E-07 | 1.87E-06 |
| ENSG00000112149 | CD83 | 1.062429 | 2.01E-07 | 2.03E-06 |
| ENSG00000076770 | MBNL3 | -1.03148 | 2.78E-07 | 2.75E-06 |
| ENSG00000283782 | AC116366.3 | -2.1078 | 2.78E-07 | 2.75E-06 |
| ENSG00000150281 | CTF1 | -1.06173 | 3.12E-07 | 3.06E-06 |
| ENSG00000177943 | MAMDC4 | -1.60618 | 3.25E-07 | 3.19E-06 |
| ENSG00000132010 | ZNF20 | 1.102234 | 3.30E-07 | 3.22E-06 |
| ENSG00000169085 | VXN | -2.57009 | 3.48E-07 | 3.38E-06 |
| ENSG00000176406 | RIMS2 | -1.25536 | 3.54E-07 | 3.43E-06 |
| ENSG00000175899 | A2M | -6.90087 | 3.62E-07 | 3.50E-06 |
| ENSG00000127423 | AUNIP | 1.396791 | 4.97E-07 | 4.68E-06 |
| ENSG00000206073 | SERPINB4 | 1.019687 | 5.27E-07 | 4.93E-06 |
| ENSG00000132394 | EEFSEC | -1.01544 | 5.54E-07 | 5.16E-06 |
| ENSG00000115392 | FANCL | 1.065879 | 5.76E-07 | 5.35E-06 |
| ENSG00000189410 | SH2D5 | -1.21195 | 6.28E-07 | 5.80E-06 |
| ENSG00000241360 | PDXP | 1.019256 | 9.81E-07 | 8.77E-06 |
| ENSG00000078018 | MAP2 | -1.45098 | 9.97E-07 | 8.90E-06 |
| ENSG00000102882 | MAPK3 | -1.04079 | 1.26E-06 | 1.10E-05 |
| ENSG00000157379 | DHRS1 | 1.151144 | 1.29E-06 | 1.12E-05 |
| ENSG00000251537 | AC005324.3 | -1.4892 | 1.34E-06 | 1.16E-05 |
| ENSG00000166432 | ZMAT1 | -1.3757 | 1.67E-06 | 1.43E-05 |
| ENSG00000254122 | PCDHGB7 | -1.23178 | 1.74E-06 | 1.48E-05 |
| ENSG00000164220 | F2RL2 | -1.29177 | 1.88E-06 | 1.60E-05 |
| ENSG00000169760 | NLGN1 | -1.71753 | 1.95E-06 | 1.66E-05 |
| ENSG00000100867 | DHRS2 | -3.07973 | 1.96E-06 | 1.66E-05 |
| ENSG00000153292 | ADGRF1 | 3.008989 | 2.13E-06 | 1.79E-05 |
| ENSG00000187800 | PEAR1 | -1.13088 | 2.21E-06 | 1.86E-05 |
| ENSG00000163121 | NEURL3 | -2.088 | 2.25E-06 | 1.89E-05 |
| ENSG00000077942 | FBLN1 | 1.130041 | 2.28E-06 | 1.91E-05 |
| ENSG00000139269 | INHBE | -1.12832 | 2.88E-06 | 2.36E-05 |
| ENSG00000105270 | CLIP3 | -1.14202 | 3.61E-06 | 2.90E-05 |
| ENSG00000142765 | SYTL1 | -1.73448 | 3.62E-06 | 2.90E-05 |
| ENSG00000141574 | SECTM1 | 1.408146 | 4.33E-06 | 3.42E-05 |
| ENSG00000185339 | TCN2 | -1.04424 | 4.69E-06 | 3.66E-05 |
| ENSG00000162490 | DRAXIN | 1.37707 | 4.69E-06 | 3.67E-05 |
| ENSG00000075340 | ADD2 | -1.25835 | 7.64E-06 | 5.72E-05 |
| ENSG00000135114 | OASL | 1.097517 | 9.23E-06 | 6.80E-05 |
| ENSG00000140379 | BCL2A1 | 2.168542 | 9.38E-06 | 6.90E-05 |
| ENSG00000160781 | PAQR6 | -1.39417 | 9.78E-06 | 7.17E-05 |
| ENSG00000158292 | GPR153 | 1.275832 | 1.13E-05 | 8.23E-05 |
| ENSG00000198945 | L3MBTL3 | 1.096215 | 1.17E-05 | 8.44E-05 |
| ENSG00000115525 | ST3GAL5 | 1.124906 | 1.32E-05 | 9.42E-05 |
| ENSG00000160256 | FAM207A | 1.412874 | 1.35E-05 | 9.62E-05 |
| ENSG00000111319 | SCNN1A | -1.34453 | 1.47E-05 | 0.000104197 |
| ENSG00000075240 | GRAMD4 | -1.01973 | 1.55E-05 | 0.000109037 |
| ENSG00000171951 | SCG2 | -1.15973 | 1.58E-05 | 0.000110966 |
| ENSG00000095739 | BAMBI | 1.041619 | 1.75E-05 | 0.00012162 |
| ENSG00000166997 | CNPY4 | 1.329946 | 2.17E-05 | 0.000148534 |
| ENSG00000280893 | AC009133.6 | 1.953661 | 2.51E-05 | 0.00016916 |
| ENSG00000154920 | EME1 | 1.437228 | 2.51E-05 | 0.000169221 |
| ENSG00000169994 | MYO7B | -1.80453 | 2.61E-05 | 0.000174707 |
| ENSG00000149403 | GRIK4 | -1.39689 | 2.68E-05 | 0.000179347 |
| ENSG00000147536 | GINS4 | 1.10212 | 2.72E-05 | 0.000181759 |
| ENSG00000126785 | RHOJ | -1.00576 | 2.89E-05 | 0.000191564 |
| ENSG00000072163 | LIMS2 | -1.32843 | 3.05E-05 | 0.000200924 |
| ENSG00000064687 | ABCA7 | 1.170306 | 3.14E-05 | 0.000206327 |
| ENSG00000075213 | SEMA3A | -1.13894 | 3.79E-05 | 0.000244612 |
| ENSG00000164619 | BMPER | 1.284815 | 4.56E-05 | 0.000288939 |
| ENSG00000144063 | MALL | -2.23406 | 4.61E-05 | 0.000291328 |
| ENSG00000081853 | PCDHGA2 | -1.49476 | 5.51E-05 | 0.000342063 |
| ENSG00000241697 | TMEFF1 | 1.588629 | 6.54E-05 | 0.000397795 |
| ENSG00000167306 | MYO5B | -1.93963 | 6.57E-05 | 0.000399169 |
| ENSG00000153294 | ADGRF4 | 4.209453 | 8.86E-05 | 0.00052269 |
| ENSG00000176401 | EID2B | 1.213639 | 8.89E-05 | 0.000523986 |
| ENSG00000171115 | GIMAP8 | 7.291554 | 9.07E-05 | 0.000532956 |
| ENSG00000215182 | MUC5AC | -7.80305 | 9.41E-05 | 0.000550593 |
| ENSG00000177294 | FBXO39 | -2.22239 | 9.42E-05 | 0.000550887 |
| ENSG00000091513 | TF | -1.81831 | 9.68E-05 | 0.000565059 |
| ENSG00000197191 | CYSRT1 | 1.788901 | 9.69E-05 | 0.000565229 |
| ENSG00000133083 | DCLK1 | -1.00646 | 0.000112282 | 0.000647508 |
| ENSG00000100311 | PDGFB | 1.237039 | 0.000121991 | 0.000696803 |
| ENSG00000139971 | ARMH4 | 1.127281 | 0.000122144 | 0.000697388 |
| ENSG00000162654 | GBP4 | 1.098778 | 0.00012428 | 0.000707286 |
| ENSG00000179715 | PCED1B | -1.63716 | 0.000125119 | 0.000711429 |
| ENSG00000138376 | BARD1 | 1.043381 | 0.000126818 | 0.000719903 |
| ENSG00000185499 | MUC1 | -1.61262 | 0.000129033 | 0.000730075 |
| ENSG00000171931 | FBXW10 | -1.12286 | 0.000130363 | 0.000736997 |
| ENSG00000113739 | STC2 | -1.16146 | 0.000137467 | 0.000772727 |
| ENSG00000272196 | HIST2H2AA4 | 1.224587 | 0.000208327 | 0.00112126 |
| ENSG00000212724 | KRTAP2-3 | 2.511714 | 0.000263495 | 0.001381546 |
| ENSG00000118785 | SPP1 | -1.68101 | 0.000275504 | 0.001440683 |
| ENSG00000135678 | CPM | 1.398772 | 0.000294036 | 0.001523777 |
| ENSG00000187134 | AKR1C1 | -1.62372 | 0.000318806 | 0.001637945 |
| ENSG00000205923 | CEMP1 | 5.145677 | 0.000358642 | 0.001817578 |
| ENSG00000162062 | TEDC2 | 1.848447 | 0.000478343 | 0.002352504 |
| ENSG00000130475 | FCHO1 | -1.49158 | 0.000482452 | 0.002367651 |
| ENSG00000132207 | SLX1A | 4.406964 | 0.000518452 | 0.002522791 |
| ENSG00000226742 | HSBP1L1 | -1.14591 | 0.000530275 | 0.002574875 |
| ENSG00000273331 | TM4SF19-TCTEX1D2 | 1.226696 | 0.000545222 | 0.002640022 |
| ENSG00000110203 | FOLR3 | 4.274481 | 0.000548661 | 0.002655742 |
| ENSG00000213398 | LCAT | -1.28036 | 0.000558897 | 0.002699605 |
| ENSG00000100302 | RASD2 | 1.256062 | 0.000574259 | 0.002759321 |
| ENSG00000188064 | WNT7B | 1.106915 | 0.000583317 | 0.002800894 |
| ENSG00000158406 | HIST1H4H | 1.053467 | 0.000614258 | 0.002933135 |
| ENSG00000286190 | AC055839.2 | -1.17759 | 0.000669381 | 0.00316243 |
| ENSG00000168237 | GLYCTK | -1.30947 | 0.000684304 | 0.003216413 |
| ENSG00000255071 | SAA2-SAA4 | 1.676684 | 0.000707759 | 0.003315369 |
| ENSG00000007968 | E2F2 | 1.532874 | 0.000744842 | 0.003467888 |
| ENSG00000250722 | SELENOP | -2.65662 | 0.000774104 | 0.003577572 |
| ENSG00000175643 | RMI2 | 1.096002 | 0.000805858 | 0.003706947 |
| ENSG00000215126 | CBWD6 | -1.01788 | 0.000838508 | 0.003836674 |
| ENSG00000196826 | AC008758.1 | 6.643856 | 0.000867912 | 0.003948957 |
| ENSG00000182162 | P2RY8 | 2.547488 | 0.000891752 | 0.004044853 |
| ENSG00000163431 | LMOD1 | -1.7028 | 0.000891919 | 0.004044853 |
| ENSG00000138622 | HCN4 | 1.956931 | 0.000910285 | 0.004120019 |
| ENSG00000101115 | SALL4 | 1.683816 | 0.000935013 | 0.004219485 |
| ENSG00000186364 | NUDT17 | -1.00297 | 0.000936572 | 0.004223929 |
| ENSG00000267740 | AC024592.3 | -1.025 | 0.000945872 | 0.004257511 |
| ENSG00000286905 | AC108488.2 | -1.07912 | 0.000993427 | 0.004449625 |
| ENSG00000165474 | GJB2 | 1.091281 | 0.001079699 | 0.004789409 |
| ENSG00000134259 | NGF | -1.20778 | 0.001127183 | 0.004971278 |
| ENSG00000165197 | VEGFD | -2.71484 | 0.00118592 | 0.00519711 |
| ENSG00000260238 | PMF1-BGLAP | 1.064012 | 0.001210724 | 0.00529404 |
| ENSG00000080298 | RFX3 | -1.07966 | 0.001212514 | 0.005297643 |
| ENSG00000171219 | CDC42BPG | -1.78427 | 0.001236377 | 0.005385735 |
| ENSG00000182901 | RGS7 | -1.42884 | 0.001249987 | 0.005433013 |
| ENSG00000115226 | FNDC4 | 1.437267 | 0.001341915 | 0.005785202 |
| ENSG00000136960 | ENPP2 | 1.666492 | 0.001377269 | 0.005917227 |
| ENSG00000135750 | KCNK1 | 1.06463 | 0.00143789 | 0.006133811 |
| ENSG00000115844 | DLX2 | -1.28063 | 0.001463688 | 0.006230379 |
| ENSG00000112559 | MDFI | -1.28504 | 0.001555856 | 0.006580085 |
| ENSG00000196188 | CTSE | -2.45943 | 0.001595283 | 0.006722107 |
| ENSG00000110944 | IL23A | 1.833058 | 0.001702904 | 0.007111152 |
| ENSG00000253485 | PCDHGA5 | -1.23133 | 0.001703069 | 0.007111152 |
| ENSG00000038427 | VCAN | -2.17932 | 0.001759729 | 0.007310131 |
| ENSG00000000971 | CFH | -1.33842 | 0.001865671 | 0.007706154 |
| ENSG00000118513 | MYB | 1.954196 | 0.001870438 | 0.007721224 |
| ENSG00000275493 | AL627230.1 | -10.2167 | 0.002007569 | 0.008191736 |
| ENSG00000167272 | POP5 | 1.049162 | 0.00219862 | 0.008861262 |
| ENSG00000173702 | MUC13 | -5 | 0.002215175 | 0.008912365 |
| ENSG00000197253 | TPSB2 | -1.73697 | 0.002265556 | 0.009091212 |
| ENSG00000268975 | MIA-RAB4B | 1.930098 | 0.002278316 | 0.009139755 |
| ENSG00000133106 | EPSTI1 | -1.25904 | 0.00232061 | 0.009279748 |
| ENSG00000253846 | PCDHGA10 | -1.2076 | 0.002340625 | 0.009348949 |
| ENSG00000128422 | KRT17 | -3.64 | 0.002341453 | 0.009349391 |
| ENSG00000249624 | AP000295.1 | 1.156276 | 0.002388264 | 0.009503473 |
| ENSG00000228253 | MT-ATP8 | -1.65156 | 0.00255174 | 0.010038202 |
| ENSG00000180998 | GPR137C | 1.062464 | 0.002613233 | 0.010245061 |
| ENSG00000129757 | CDKN1C | -1.13235 | 0.002718341 | 0.010584962 |
| ENSG00000286088 | AC073585.2 | 7.321928 | 0.003043243 | 0.011672251 |
| ENSG00000214814 | FER1L6 | 2.807355 | 0.003044515 | 0.011673882 |
| ENSG00000243414 | TICAM2 | 3.044394 | 0.003206276 | 0.012192509 |
| ENSG00000128408 | RIBC2 | 1.924813 | 0.003366804 | 0.012708397 |
| ENSG00000149573 | MPZL2 | -1.35184 | 0.00344509 | 0.012969563 |
| ENSG00000168306 | ACOX2 | -1.5221 | 0.003452432 | 0.012992125 |
| ENSG00000177989 | ODF3B | -2.34031 | 0.003563006 | 0.01335039 |
| ENSG00000133101 | CCNA1 | -1.05956 | 0.00363474 | 0.013589654 |
| ENSG00000153404 | PLEKHG4B | 2.273018 | 0.003654554 | 0.013648943 |
| ENSG00000183128 | CALHM3 | -1.49399 | 0.003661081 | 0.013665922 |
| ENSG00000176020 | AMIGO3 | -3.39689 | 0.003889378 | 0.014391097 |
| ENSG00000285991 | AL355312.6 | 2.253757 | 0.003889768 | 0.014391097 |
| ENSG00000180616 | SSTR2 | 1.140481 | 0.004296032 | 0.015646673 |
| ENSG00000179627 | ZBTB42 | 1.258016 | 0.004557678 | 0.016447686 |
| ENSG00000183647 | ZNF530 | 1.270891 | 0.004575848 | 0.01650894 |
| ENSG00000267059 | AC005943.1 | -1.30068 | 0.0047111 | 0.016912898 |
| ENSG00000180509 | KCNE1 | -2.35252 | 0.004826707 | 0.017278484 |
| ENSG00000187824 | TMEM220 | -1.27417 | 0.005047716 | 0.01791167 |
| ENSG00000275778 | AC018630.2 | -1.00753 | 0.00505007 | 0.017915417 |
| ENSG00000168032 | ENTPD3 | 1.548893 | 0.00518528 | 0.018300989 |
| ENSG00000109472 | CPE | -1.37638 | 0.005206897 | 0.018358503 |
| ENSG00000170381 | SEMA3E | -1.13409 | 0.005235266 | 0.018444388 |
| ENSG00000057657 | PRDM1 | 1.220048 | 0.005358428 | 0.018806284 |
| ENSG00000175322 | ZNF519 | 1.394279 | 0.005602351 | 0.019523345 |
| ENSG00000161939 | RNASEK-C17orf49 | 1.223331 | 0.00573629 | 0.019924691 |
| ENSG00000204262 | COL5A2 | -1.1304 | 0.005782283 | 0.020064616 |
| ENSG00000138190 | EXOC6 | -1.18552 | 0.005803105 | 0.02012132 |
| ENSG00000275074 | NUDT18 | -1.50834 | 0.005949361 | 0.02053703 |
| ENSG00000179331 | RAB39A | 1.664133 | 0.006490818 | 0.022062386 |
| ENSG00000186469 | GNG2 | -1.40078 | 0.006551917 | 0.022242672 |
| ENSG00000135925 | WNT10A | -1.09289 | 0.006646379 | 0.022532345 |
| ENSG00000132837 | DMGDH | -1.63227 | 0.006727173 | 0.022753673 |
| ENSG00000138769 | CDKL2 | -1.09115 | 0.006754944 | 0.022830818 |
| ENSG00000285920 | AC087721.2 | -3.24793 | 0.006816533 | 0.023016433 |
| ENSG00000118137 | APOA1 | -4.30117 | 0.006977255 | 0.023467248 |
| ENSG00000131650 | KREMEN2 | 1.982722 | 0.0071977 | 0.024121726 |
| ENSG00000149418 | ST14 | -1.08246 | 0.00735008 | 0.024571489 |
| ENSG00000257446 | ZNF878 | 1.771376 | 0.007834577 | 0.025939818 |
| ENSG00000154493 | C10orf90 | 1.584963 | 0.008000858 | 0.026376456 |
| ENSG00000112837 | TBX18 | -3.24793 | 0.008050213 | 0.02649053 |
| ENSG00000197978 | GOLGA6L9 | -1.10813 | 0.008120091 | 0.026672235 |
| ENSG00000108771 | DHX58 | -1.31893 | 0.008274735 | 0.027136727 |
| ENSG00000165092 | ALDH1A1 | -6.71081 | 0.008355382 | 0.027355698 |
| ENSG00000198074 | AKR1B10 | -3.2363 | 0.008398402 | 0.027476992 |
| ENSG00000197568 | HHLA3 | -1.29078 | 0.008721821 | 0.028380364 |
| ENSG00000120328 | PCDHB12 | -1.11847 | 0.008829189 | 0.028682394 |
| ENSG00000284906 | ARHGAP11B | -1.0972 | 0.008990602 | 0.029122155 |
| ENSG00000122432 | SPATA1 | -2.51694 | 0.009216845 | 0.029717865 |
| ENSG00000167550 | RHEBL1 | 1.657475 | 0.00938438 | 0.030152374 |
| ENSG00000152580 | IGSF10 | 1.784271 | 0.009730082 | 0.031047529 |
| ENSG00000168955 | TM4SF20 | -4.32193 | 0.010130392 | 0.032152577 |
| ENSG00000270757 | HSPE1-MOB4 | 4.075949 | 0.010580901 | 0.033344698 |
| ENSG00000163689 | C3orf67 | -1.0091 | 0.010891872 | 0.034176405 |
| ENSG00000143919 | CAMKMT | -1.42369 | 0.010955495 | 0.034329207 |
| ENSG00000228570 | NUTM2E | 3.115477 | 0.011198397 | 0.034922345 |
| ENSG00000117425 | PTCH2 | -1.12199 | 0.011224046 | 0.03497219 |
| ENSG00000170442 | KRT86 | -1.21573 | 0.0116819 | 0.036105311 |
| ENSG00000180316 | PNPLA1 | -1.41871 | 0.011815811 | 0.036453877 |
| ENSG00000157890 | MEGF11 | -2.69561 | 0.011851684 | 0.036548211 |
| ENSG00000106560 | GIMAP2 | 4.017922 | 0.012779238 | 0.038955927 |
| ENSG00000117983 | MUC5B | -5.80735 | 0.012792329 | 0.038987221 |
| ENSG00000179841 | AKAP5 | 1.300395 | 0.013170668 | 0.039972556 |
| ENSG00000168273 | SMIM4 | 1.299489 | 0.013646535 | 0.041100433 |
| ENSG00000187595 | ZNF385C | -1.76275 | 0.013811003 | 0.041505191 |
| ENSG00000131095 | GFAP | -2.36457 | 0.013899966 | 0.04172711 |
| ENSG00000172476 | RAB40A | -1.35199 | 0.014265989 | 0.042603472 |
| ENSG00000105538 | RASIP1 | -1.82716 | 0.014583298 | 0.043326053 |
| ENSG00000133048 | CHI3L1 | -3.14296 | 0.014678291 | 0.043552013 |
| ENSG00000155792 | DEPTOR | 1.208317 | 0.015008035 | 0.044339731 |
| ENSG00000196730 | DAPK1 | -3.04439 | 0.01521923 | 0.044896403 |
| ENSG00000284969 | AL049629.2 | 1.415619 | 0.015415525 | 0.045368789 |
| ENSG00000080493 | SLC4A4 | 1.286881 | 0.015471101 | 0.045503238 |
| ENSG00000086548 | CEACAM6 | -11.7566 | 0.015747127 | 0.046206753 |
| ENSG00000088386 | SLC15A1 | 1.791413 | 0.016034742 | 0.046950868 |
| ENSG00000103154 | NECAB2 | -1.8009 | 0.016283272 | 0.047517266 |
| ENSG00000068615 | REEP1 | 1.013056 | 0.016294219 | 0.047539159 |
| ENSG00000185955 | C7orf61 | 2.30117 | 0.016356501 | 0.047670477 |
| ENSG00000004776 | HSPB6 | -2.72935 | 0.016512371 | 0.048053716 |
| ENSG00000109511 | ANXA10 | 1.682428 | 0.016643944 | 0.048375406 |
| ENSG00000142675 | CNKSR1 | -1.00978 | 0.017031364 | 0.049387018 |
