# Supplementary material for: FDI-6 inhibits the expression and function of FOXM1 to sensitize BRCA-proficient triple-negative breast cancer cells to Olaparib by regulating cell cycle progression and DNA damage repair
Source: Cell Death Dis. 2021 Dec 8;12(12):1138. doi: 10.1038/s41419-021-04434-9 (PMC8654856; doi:10.1038/s41419-021-04434-9)
Supplement: Supplementary file 23 — Author contribution [file 41419_2021_4434_MOESM23_ESM.doc]

**Attribution of Authorship**

for

FDI-6 inhibits the expression and function of FOXM1 to sensitize BRCA-proficient triple-negative breast cancer cells to Olaparib by regulating cell cycle progression and DNA damage repair

Shu-Ping Wang^1,§,*^, Shi-Qi Wu^1,§^, Shi-Hui Huang^1^, Yi-Xuan Tang^1^, Liu-Qiong Meng^1^, Feng Liu^1^, Qi-Hua Zhu^2^, Yun-Gen Xu^1,2,*^

^1^State Key Laboratory of Natural Medicines and Jiangsu Key Laboratory of Drug Design and Optimization, China Pharmaceutical University, 211198 Nanjing, China.

^2^Jiangsu Key Laboratory of Drug Design and Optimization, Department of Medicinal Chemistry, China Pharmaceutical University, Nanjing 210009, China.

^§^The authors contributed equally: Shu-Ping Wang, Shi-Qi Wu.

**Author contributions**

***Contribution to preparation of manuscript***

1. Dr. Shuping Wang was responsible for the RNA sequencing, animal care and treatment, bioinformatics analysis and design of this study, participated in all experiments, the analysis and interpretation of data, and wrote the manuscript.
2. Shiqi Wu was responsible for cell culture, the Q-PCR experiments, Western-blots experiments, colony formation assay, alkaline comet assay and immunofluorescence assay, participated in the analysis, interpretation of data and writing of the manuscript.
3. Shihui Huang was responsible for cell viability assay, drug combination assay, shRNA lentivirus infection and the detection of cell apoptosis and cell cycle, participated in interpretation of data.
4. Yixuan Tang was responsible for the synthesis of FDI-6 and the selectivity detection of FDI-6.
5. Liuqiong Meng was responsible for H&E staining and immunohistochemical staining.
6. Feng Liu was participated in Q-PCR and Western-blots experiments.
7. Dr. Qihua Zhu participated in writing of the manuscript.
8. Dr. Yungen Xu participated in the design of the study, the analysis and interpretation of data, and writing of the manuscript.
